# Conservative significance testing of tripartite statistical relations in multivariate neural data

**DOI:** 10.1162/netn_a_00259

**Published:** 2022-10-01

**Authors:** Aleksejs Fomins, Yaroslav Sych, Fritjof Helmchen

**Affiliations:** Brain Research Institute, University of Zurich, Zurich, Switzerland; Neuroscience Center Zurich, University of Zurich, Switzerland; Experimental Neurology Center, Department of Neurology, Inselspital University Hospital Bern, Bern, Switzerland; Present address: Institute of Cellular and Integrative Neurosciences, University of Strasbourg and CNRS, Strasbourg, France

**Keywords:** Significance testing, Partial information decomposition, Functional connectivity, Synergy, Redundancy, Multicollinearity

## Abstract

An important goal in systems neuroscience is to understand the structure of neuronal interactions, frequently approached by studying functional relations between recorded neuronal signals. Commonly used pairwise measures (e.g., correlation coefficient) offer limited insight, neither addressing the specificity of estimated neuronal interactions nor potential synergistic coupling between neuronal signals. Tripartite measures, such as partial correlation, variance partitioning, and partial information decomposition, address these questions by disentangling functional relations into interpretable information atoms (unique, redundant, and synergistic). Here, we apply these tripartite measures to simulated neuronal recordings to investigate their sensitivity to noise. We find that the considered measures are mostly accurate and specific for signals with noiseless sources but experience significant bias for noisy sources.We show that permutation testing of such measures results in high false positive rates even for small noise fractions and large data sizes. We present a conservative null hypothesis for significance testing of tripartite measures, which significantly decreases false positive rate at a tolerable expense of increasing false negative rate. We hope our study raises awareness about the potential pitfalls of significance testing and of interpretation of functional relations, offering both conceptual and practical advice.

## Author Summary

Tripartite functional relation measures enable the study of interesting effects in neural recordings, such as redundancy, functional connection specificity, and synergistic coupling. However, estimators of such relations are commonly validated using noiseless signals, whereas neural recordings typically contain noise. Here we systematically study the performance of tripartite estimators using simulated noisy neural signals. We demonstrate that permutation testing is not a robust procedure for inferring ground truth statistical relations from commonly used tripartite relation estimators. We develop an adjusted conservative testing procedure, reducing false positive rates of the studied estimators when applied to noisy data. Besides addressing significance testing, our results should aid in accurate interpretation of tripartite functional relations and functional connectivity.

## INTRODUCTION

Recent advances in brain recording techniques enable simultaneous acquisition of multiple neuronal signals. Examples are single-cell population recording techniques, such as multielectrode arrays (Stevenson & Kording, 2011) or two-photon calcium imaging (Chen et al., 2013), as well as multiregional population-average recording techniques, such as wide-field imaging (Gallero-Salas et al., 2021), multifiber photometry (Sych, Chernysheva, Sumanovski, & Helmchen, 2019), EEG (Michel & Brunet, 2019), MEG (Cheyne, 2013), or fMRI (Heeger & Ress, 2002). An important stepping stone to understand neural coding is the ability to robustly inferand interpret possible functional/statistical relations between multivariate signal components, be it single neurons or population-averaged regional signals. At first glance, the procedure may appear as simple as computing a standard relational measure, such as Pearson’s correlation coefficient, followed by reporting the pairs of signals with high or low coefficient values. However, a finer inspection reveals several pitfalls of such an approach. The aim of this paper is to illuminate one such pitfall, discuss its implications, and propose a solution. Specifically, we address the negative effects of additive noise on the robustness of functional relation estimates.

Functional relations can be defined via a model-based approach. A general model will attempt to explain one of the signals, known as the dependent variable (or simply the target), by means of other signals, known as the independent variables (or sources, or predictors). The special case of considering a single source is covered by the well-studied fields of pairwise functional connectivity (Friston, 1994) and effective connectivity (Greicius, Supekar, Menon, & Dougherty, 2008). Introduction of multiple sources enables the study of interesting higher order effects, such as confounding effects on pairwise connections as well as synergistic effects between sources. Here, we focus our attention on two source variables, that is, on tripartite measures. The use of tripartite functional relations in addition to functional connectivity may pave the way toward causal relation interpretations of neuronal recordings (Reid et al., 2019), albeit not without shortcomings (Mehler & Kording, 2018) or additional research. While considering a larger number of source variables is possible in principle (P. L. Williams & Beer, 2010), it is challenging in practice, since the number of possible types of higher order relations grows exponentially with the number of variables, as does the data size required for robust estimation of such relations.

**Figure 1.**
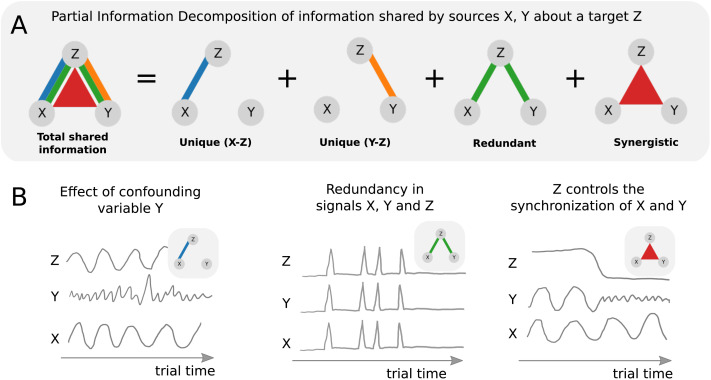
(A) Sketch of partial information decomposition. Sketches of this form will be employed throughout this paper. The colors will always denote the corresponding information atoms 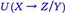, 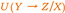, 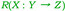, 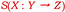. The width of individual lines or triangles qualitatively indicates the magnitude of the effect. In this plot, all information atoms are shown with maximal magnitude for reference. (B) Example questions about tripartite relations that may be of interest in neuroscience. Left: Is the functional connection between *X* and *Z* specific with respect to the confounding variable *Y*? Middle: Are *X*, *Y*, and *Z* redundantly encoding the same information? Right: Could *Z* control synchronization between *X* and *Y*? (for example, if *X* and *Y* control forelimbs and hind limbs, respectively, and *Z* determines if the animal is currently running or resting). Note: the three sketches are made as a function of time for illustrative purposes only. In principle, information atoms can be computed across any data dimension. Here, we compute information atoms across trials.

A pair of source variables *X* and *Y* may contain information about a target variable *Z* in four distinct ways (P. L. Williams & Beer, 2010), called information atoms (see Table 1 and Figure 1). We aim to reveal how well different measures framed in this formalism can recover ground truth information in simulated multivariate recordings. Two concepts that make such estimation challenging are redundancy and noise, which we introduce in the following.

**Table 1.** Four information atoms of partial information decomposition (P. L. Williams & Beer, 2010)

Type	Expression	Description
Unique information	*U*(*X* → *Z*|*Y*)	Source *X* may contain **unique** information about the target *Z*, not present in the source *Y*
Unique information	*U*(*Y* → *Z*|*X*)	Source *Y* may contain **unique** information about the target *Z*, not present in the source *X*
Redundant information	*R*(*X* : *Y* → *Z*)	Both sources may **redundantly** share some information about the target, available from either of the sources
Synergistic information	*S*(*X* : *Y* → *Z*)	Both sources may **synergistically** share some information about the target, available from synergy between the sources, but not from either source individually.

*Note*. *X*, *Y*, and *Z* are three recorded variables (e.g., neuronal signals). Here, *X* and *Y* are the independent (source) variables, and *Z* is the dependent (target) variable.

We first consider redundancy. A common method for studying linear relations between source and target variables is Multi-way ANalysis Of VAriance (ANOVA) (Gelman, 2005). It provides information about the overall goodness of fit of a model as well as about the expected magnitude and significance of individual coefficients. While ANOVA is known to provide robust estimates of coefficient significance when the source variables are mostly unrelated (Andrews, 1974), it fails to do so when the source variables are related. This phenomenon is known as multicollinearity (Farrar & Glauber, 1967) in statistics literature and as redundancy in neuroscience (Hennig et al., 2018). In case of redundancy, a broad range of parameter value combinations may result in an optimal model fit. Hence, multiple different parameter combinations may be indistinguishable to the fitting procedure. In such case, ANOVA will arbitrarily report some parameter values resulting in a good fit, with unreliable estimates of parameter significance (Farrar & Glauber, 1967). This effect is undesirable, as we ultimately want to know the importance and specificity of individual sources as predictors. Importantly, high redundancy is common in both single-neuron recordings (Fuster, 1973) and in multiregional population-average recordings (Gallero-Salas et al., 2021; Sych, Fomins, Novelli, & Helmchen, 2020), and thus needs to be accounted for.

Next we consider noise. Neuronal recordings frequently do not directly access the neuronal variables of interest. Apart from instrumental noise, observables may be corrupted by various other factors including imperfect knowledge of the properties of the signal proxy (e.g., calcium indicator or BOLD fMRI responses), contamination by neuropil fluorescence signals, or cross-talk, and heart-beat or movement-induced artifacts. Although such impurities are typically acknowledged in the experimental literature, they often are overlooked in statistical analyses such as functional connectivity estimation. Consider a simple linear model

$$Z=aX+bY+\nu_{z}$$ (1)

where *Z* is the target variable, *X* and *Y* are the source variables, *a* and *b* are the corresponding coefficients, and *ν*_*z*_ is the residual error. In this case, *Z* is corrupted by the additive error *ν*_*z*_. While part of it may be due to experimental limitations as described above, signal impurity may also arise due to other sources that have not been observed in the experiment. For example, the mood of a cat may be affected by weather and the quality of their meal, but also by the amount of petting they have received. An optimal model that includes all of these sources will have lower residual variance in explaining the cat’s mood than an optimal model that does not include petting. The unexplained variance in the latter model is also part of what is commonly called noise, even though it could have been accounted for by recording more observables such as petting. Such scenarios are common in neuroscience. For example, a population-average signal may represent multiple distinct neuronal subpopulations with different functional connectivity, such that only part of the observed signal correlates with the signal of interest (e.g., the activity in another brain area). Similarly, an individual neuron may integrate multiple inputs, of which not all are recorded. Impurity of observables in terms of residual variance thus does not solely reflect limitations of the measurement techniques, but also the incompleteness of observing all relevant sources.

**Figure 2.**
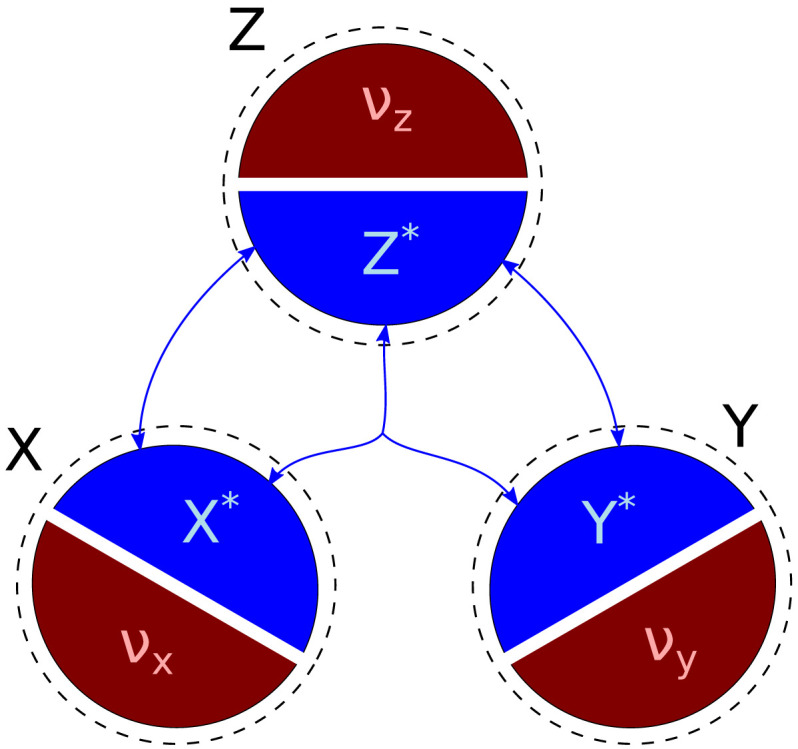
Noise in neuronal observables. A typical aim is the estimation of information atoms (blue arrows) between neuronal signals of interest (blue areas X*, Y*, and Z*) underlying the recorded data. However, the observables the experimenter has access to (black areas *X*, *Y*, and *Z*) typically are not the pure signals of interest. In the simplest case considered here, observables are corrupted by additive noise (red *ν*_*x*_, *ν*_*y*_, and *ν*_*z*_). Blue arrows in the middle indicate tripartite interaction effects between the signals of interest (i.e., synergy).

Direct access to source variables is also not a given. For example, the recorded observables of source variables may contain additive noise *ν*_*x*_ and *ν*_*y*_ of similar origins as described above for the target variable. In general, all three observables may be noisy (Figure 2). For simplicity, we will only consider additive errors, although in general the relation may be more complex. We will denote the underlying neuronal variables with an asterisk (e.g., X*) and the corresponding observables without one (e.g., *X*). The noise terms *ν*_*x*_, *ν*_*y*_, and *ν*_*z*_ are assumed to be statistically independent in this work.

$$X=X^{*}+\nu_{x}$$ (2)

$$Y=Y^{*}+\nu_{y}$$ (3)

$$Z=Z^{*}+\nu_{z}$$ (4)

We will quantify the noisiness of an observable by means of *noise fractions* (see Methods):

$${NF}_{X}=\frac{\sigma_{\nu }^{2}}{\sigma_{\nu }^{2}+\sigma_{x}^{2}}=\frac{1}{1+SNR}$$ (5)

Noise fractions have values between 0 and 1, where 0 denotes a signal with no residual errors, and 1 denotes a signal consisting only of residual errors. It is related to signal-to-noise ratio (SNR) that is commonly used in signal theory. However, SNR does not cover the case of 100% signal, which we find interesting to consider.

Many measures are designed to estimate functional relations (functional connectivity or information atoms) between noiseless variables (discrete-variable case), or variables with noiseless sources (continuous-variable case). The presence of noise, especially in source variables, frequently results in violation of the assumptions of these measures, and thus may produce spurious findings. In statistics and econometrics, models aware of potential source variable noise are known as errors-in-variables models (Greene, 2003). For example, the term *regression dilution* (Hausman, 2001) describes the effect that basic linear regression will increasingly underestimate the absolute value of the regression coefficient with increasing noise fraction in the source variables. We believe that in the neuroscience community the detrimental effects of noise on multivariate estimators are less well known, motivating us to attract attention to these effects here.

Having introduced redundancy and noise, we will now outline the scope of this study. Our specific aims are to present measures designed to disentangle individual functional relations between triplets of variables in the presence of redundancy, to computationally test whether these measures are robust to noise in source and target variables, and to propose and discuss potential improvements. We focus on three existing measures: partial correlation (PCorr) (Fisher, 1924), variance partitioning (VP) (Borcard, Legendre, & Drapeau, 1992), and partial information decomposition (PID) (P. L. Williams & Beer, 2010). Precise definitions of these measures are given in the Methods section. Partial correlation has been used in neuroscience to study the specificity of functional connections between neurons (Eichler, Dahlhaus, & Sandköhler, 2003) and fMRI voxels (Fransson & Marrelec, 2008; Marrelec et al., 2006). Harris (2021) proposed a test for PCorr taking signal autocorrelation into account, which is of high relevance for neuronal signal proxies such as calcium indicator or fMRI BOLD signals. Variance partitioning, previously introduced in ecological analysis (Bienhold, Boetius, & Ramette, 2011; Borcard et al., 1992; økland & Eilertsen, 1994), was recently used to study unique and redundant feature encoding in human fMRI recordings (de Heer, Huth, Griffiths, Gallant, & Theunissen, 2017; Lescroart, Stansbury, & Gallant, 2015). The original method is based on decomposing the variance explained by a combination of sources, obtaining unique and redundant explained variances. In this paper, we extend this methodology by also including quadratic synergistic terms, thus making VP comparable to PID described below. VP is strongly related to partial R-squared (also known as partial F-test), which is a popular measure because it allows for quantitative comparison of two linear models explaining the same target variable. In neuroscience, among other fields, it has been used to compare models of hemodynamic response in fMRI (Aguirre, Zarahn, & D’Esposito, 1998), shape-selectivity in cortical areas V4/IT (Brincat & Connor, 2004), reaction time in working-memory tasks (Finke, Ostendorf, Martus, Braun, & Ploner, 2008), fatigue in multiple sclerosis (Merkelbach, König, & Sittinger, 2003), and neuronal correlates of minimal conscious state (Perri et al., 2016). Partial information decomposition is the most recent of the measures. While it has been actively developed by the information-theoretic community for a decade (P. L. Williams & Beer, 2010), it has been rapidly gaining popularity in neuroscience in the last few years. For example, PID has been used to demonstrate a relationship between synergy and feedback information flow in mouse organotypic cultures (Sherrill, Timme, Beggs, & Newman, 2021), to show significant synergy between somatic and apical dendritic output of L5b pyramidal neurons and its relationship to activation of dendritic GABA_B receptors in rat S1 slices (Schulz, Kay, Bischofberger, & Larkum, 2021), to estimate unique contributions of acoustic features of speech to BOLD responses in humans (Daube, Giordano, Schyns, & Ince, 2019; Daube, Ince, & Gross, 2019), and to explain age-related dynamics of hubs in Ising models on human connectomes (Nuzzi, Pellicoro, Angelini, Marinazzo, & Stramaglia, 2020). Further, it has been used to explore the structure of simulated input-driven recurrent network models (Candadai & Izquierdo, 2020) and artificial generative neuronal networks (Tax, Mediano, & Shanahan, 2017). We believe that PID will be increasingly applied in coming years, especially in studies addressing nonlinear confounding effects, the specificity of functional relations, and synergistic encoding.

In the following, we ask whether these measures are sensitive and specific in detecting the presence of statistical relations in simulated data with known ground truth. We consider both discrete and continuous model data, correspondingly choosing discrete and continuous tripartite measures. For discrete data, the tested measures for the most part are significant and specific, given model data with noiseless source variables. However, addition of even small noise to the source variables damages the specificity of the measures when permutation tested. Further, continuous-valued PID measures produce infinite values for noiseless data, and thus we only test them using datasets where all variables have at least some noise. For such noisy data, continuous-variable measures result in false positives similarly to the discrete-variable case.

As a partial remedy for this problem, we propose a null hypothesis that corrects the bias introduced by noise. Compared to permutation testing, this approach significantly reduces the false positive rate at the expense of increasing the false negative rate. This approach should be beneficial in exploratory neuroscience research, aiming to preserve robustness of the stronger findings at the expense of losing some of the weaker ones.

## METHODS

**Figure 3.**
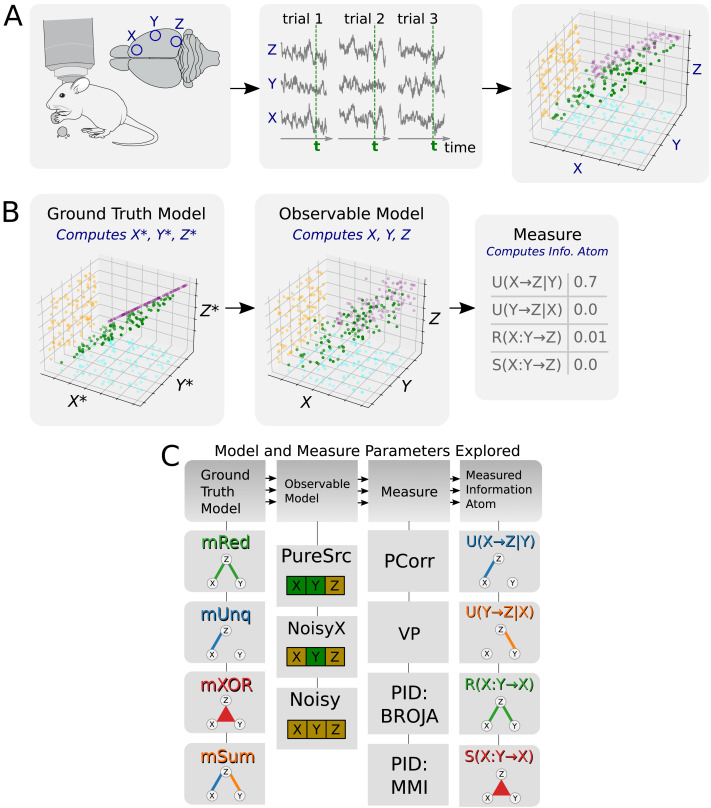
(A) A thought experiment setup. Left: Multivariate neuronal signals are recorded in a behaving test subject (courtesy to SciDraw). Middle: Neuronal signals *X*, *Y*, and *Z* are observed during *N*_*tr*_ trials with the same duration *T* and are plotted as a function of trial time for three example trials. Green vertical lines indicate a sample time step at which the analysis is performed. Right: 3D scatter plot of *X*, *Y*, and *Z* across trials sampled at the fixed time step *t* (green). 2D projections indicate that *X* correlates to *Z* (purple), while *Y* is uncorrelated to either *X* or *Z*. (B) A sketch of the simulation procedure. First the ground truth model is used to generate multiple samples of the ground truth variables X*, Y*, Z*. Then, the observable model adds noise to the data, producing observables *X*, *Y*, *Z*. Finally, the measure is used to compute information atoms for the given data sample. (C) We explored four ground truth models (mRed, mUnq, mXOR, mSum), three observable models (PureSrc, NoisyX, Noisy), four measures (PCorr, VP, BROJA PID, MMI PID), which each report four different information atoms (except PCorr, see below). In the observational model, green color denotes pure variables (no unexplained variance), and yellow denotes noisy variables. All models had discrete and continuous versions.

Let us consider the following scenario (Figure 3A): A test subject (e.g., a mouse or a human) performs a temporally structured behavioural task while brain activity is simultaneously recorded via three neuronal observables *X*, *Y*, and *Z*. Depending on the recording method, the observables may represent single-cell activity or regional bulk activity, pooled across multiple neurons. The test subject repeats the task over a set of trials, which are of equal duration and assumed to be independent and identically distributed (i.i.d.). In total, *N* = *N*_*trial*_*N*_*time*_ data points are recorded for each observable, where *N*_*trial*_ is the number of trials and *N*_*time*_ is the number of time steps in a single trial. We want to understand how the signals *X* and *Y* may be related to *Z*. More precisely, the aim is to quantify the functional relations between two source signals, *X* and *Y*, and the target signal *Z* (by means of information atoms) and to evaluate how they change over trial time. Here, we study information atoms across trials for a fixed time point. This approach satisfies the i.i.d. requirement of information atom estimators used in this study. The process can be repeated for every time step individually, which allows to build up the temporal dynamics of the information atoms. Given these assumptions, the problem of studying time-dependent evolution of functional relations between three neuronal observables is reduced to the problem of estimating the information atoms from *N*_*trial*_ i.i.d. simultaneous samples of the random variables *X*, *Y*, and *Z*. Possible extensions of the above assumptions are addressed in the Discussion.

In the following, we first present the measures that we used to estimate the tripartite functional relations. Second, we introduce three ground truth models that we used to simulate the ground truth variables at a fixed time step over trials. Third, we present observable models that we used to obtain the observable variables from the ground truth variables by adding noise. Finally, we explain the testing procedure used for testing the significance of individual information atoms. The summary of the simulation procedure and explored model and measure combinations is given in Figure 3B.

### Measures for Tripartite Analysis

#### Partial correlation (PCorr).

PCorr is the Pearson’s correlation coefficient between two random variables *X* and *Z*, controlling for the confounding variable *Y*. The control is performed by fitting *Y* to each of *X* and *Z* using linear least squares, subtracting the fits to obtain residuals,

$$X_{res}=X-fit\left(Y,X\right)$$ (6)

$$Z_{res}=Z-fit\left(Y,Z\right)$$ (7)

followed by computation of the Pearson’s correlation coefficient between the residuals:

$$\text{PCorr}\left(X,Z\right)=\text{Corr}\left(X_{res},Z_{res}\right)$$ (8)

Similarly, the partial correlation *PCorr*(*Y*, *Z*) between *Y* and *Z* can be computed by finding and correlating the residuals of both variables with respect to *X*. Here we apply PCorr to both discrete and continuous models.

PCorr is a linear version of conditional mutual information (CMI), where the latter is known to be the sum of unique and synergistic information atoms (P. L. Williams & Beer, 2010). To check if PCorr behaved similarly, we numerically compared PCorr and CMI using basic ground truth models (see Supporting Information Figure S1). We found that PCorr and CMI behave similarly in case of a sum operation *Z* = *X* + *Y*, which is known to have nonzero synergy. We also found that, unlike CMI, PCorr did not respond to the XOR operation. Nevertheless, it is clear that PCorr does conflate unique and synergistic information atoms, as defined by PID. Thus, specifically for PCorr, we focused on studying significance and specificity in redundant and unique ground truth models.

#### Variance partitioning.

Partial R-squared (*PR*^2^) is a measure generally used for quantifying the difference in performance of two linear regression models in explaining the same dependent variable. In practice, it is commonly used to evaluate the usefulness of individual independent variables. Using the three variable examples, we might want to estimate the usefulness of the source *X* as predictor of the target *Z*, given another source variable *Y*. To do so, we can construct a model *f* of two variables *Z*_*f*_ = *f*(*X*, *Y*) and another simpler model *g* without *X*, that is, *Z*_*g*_ = *g*(*Y*). After fitting both models, we can compute the residual sum of squares (SSR) for each model. SSR is the “unexplained” sum of squares, calculated after the model has been fitted to the target and the fit has been subtracted.

$${SSR}_{f}=\sum_{i}{\left|z_{i}-f\left(x_{i},y_{i}\right)\right|}^{2}$$ (9)

$${SSR}_{g}=\sum_{i}{\left|z_{i}-g\left(y_{i}\right)\right|}^{2}$$ (10)

*PR*^2^ is defined as the difference of these two residual terms. Here, backslash denotes set exclusion (i.e., /*X* denotes a model where *X* is excluded from the set of predictors; in this case only *Y* remains).

$${PR}_{X}^{2}={SSR}_{g}-{SSR}_{f}={SSR}_{/X}-{SSR}_{\text{full}}$$ (11)

*PR*^2^ can be used to define VP. First, a full model *F* with all of the predictors of interest is fitted to the target variable *Z*. The total sum of squares (*SST*_*Z*_) of the target variable can then be partitioned into the sum of squares explained by the model (*SSE*_*F*_) and the sum of squares of the residuals *SSR*_*F*_.

$${SST}_{Z}={SSE}_{F}+{SSR}_{F}$$ (12)

*SSE*_*F*_ can further be partitioned into nonnegative parts (unique *U*, redundant *R*, and synergistic *S*) similar to those defined in PID (see below). For consistency with PID, we refer to the parts of this decomposition as information atoms. We are aware that standard error does not directly measure information, and that this measure is only conceptually similar to PID.

$${SSE}_{F}=U\left(X\to Z|Y\right)+U\left(Y\to Z|X\right)+R\left(X:Y\to Z\right)+S\left(X:Y\to Z\right)$$ (13)

Here, VP is based on the application of *PR*^2^ to a simple quadratic interacting model with two independent variables.

$$Z_{\text{quad}}\left(X,Y\right)=aX+bY+cXY$$ (14)

where the last term is the coupling term between *X* and *Y*, modeling their synergistic effect on *Z*. Throughout this section, we assume that means have been subtracted from both source and target variables prior to fitting. In principle, this can also be done by additionally modeling a constant term, which we drop here for simplicity. Note that the term *XY* with the coefficient *c* is also a predictor distinct from *X* and *Y*. Even though it depends on *X* and *Y* in general, it can be shown to be linearly independent from *X* and *Y*, effectively resulting in a new predictor.

The original definition of VP (Borcard et al., 1992) includes only the first two terms, that is, modeling unique and redundant information atoms. While we are not aware of other publications using a quadratic term in this exact setting, it is commonplace to use quadratic terms to model coupling between sources in similar settings (see, e.g., Stephan et al., 2007). We can define unique and synergistic information atoms by the corresponding *PR*^2^, namely by the explained variance lost when excluding each of the terms in the model individually:

$$U\left(X\to Z|Y\right)={PR}_{/a}^{2}$$ (15)

$$U\left(Y\to Z|X\right)={PR}_{/b}^{2}$$ (16)

$$S\left(X:Y\to Z\right)={PR}_{/c}^{2}$$ (17)

For completeness, we augment the above model by also defining the redundant information atom.

$$R\left(X:Y\to Z\right)={SST}_{Z}-\left({SSR}_{lin,x}+{SSR}_{lin,y}-{SSR}_{lin,x,y}\right)$$ (18)

Here, *SSR*_*lin*,*x*_, *SSR*_*lin*,*y*_, and *SSR*_*lin*,*x*,*y*_ are the residual sums of squares corresponding to linear models containing only the source *X*, only source *Y*, and both sources *X* and *Y*, respectively. The derivation of the *R*(*X* : *Y* → *Z*) is more technical and is thus treated in the Supporting Information. In all plots, VP information atoms are normalized by *SST*_*Z*_ to obtain a dimensionless number between [0, 1]. Loosely, this number can be interpreted as the fraction of total variance explained by each information atom, although some authors have argued that this interpretation may be misleading (Achen, 1990). Normalization does not affect significance testing and is done for aesthetic purposes only. Thus, we only make statements about relative values of VP information atoms, and make no statements about the interpretation of the absolute values.

Besides studying unique information atoms similar to PCorr, VP can also estimate redundant and synergistic information atoms, similar to PID discussed below. However, VP is only an approximation for relations beyond linear, and the synergistic term is only sensitive to interactions that have a nonnegligible quadratic component. Here, we apply VP to both discrete and continuous model data.

#### Partial information decomposition (PID).

PID is a decomposition of the Shannon mutual information shared by a pair of source variables *X*, *Y*, and a target variable *Z* (given by the mutual information *I*(*X*, *Y* : *Z*)) into independent information atoms (P. L. Williams & Beer, 2010).

$$I\left(X,Y:Z\right)=U\left(X\to Z|Y\right)+U\left(Y\to Z|X\right)+R\left(X:Y\to Z\right)+S\left(X:Y\to Z\right)$$ (19)

Similar to the other measures described, unique information atoms (*U*(*X* → *Z*|*Y*) or *U*(*Y* → *Z*|*X*)) measure the information shared by the target and one of the source variables but not the other one, redundant information atoms *R*(*X* : *Y* → *Z*) measure the information shared by the target and either one of the source variables, and synergistic information atoms *S*(*X* : *Y* → *Z*) measure the information shared by the target and both the source variables but not shared by either of them independently. In theory, PID can resolve arbitrarily nonlinear statistical relations between random variables. In practice, the resolution of the measure is limited by the number of data points available. In its original formulation (P. L. Williams & Beer, 2010) PID is a nonnegative decomposition; however, this is not the case for more recent PID measures (C. Finn & Lizier, 2018a; Ince, 2017; Makkeh, Theis, & Vicente, 2018) that follow different interpretations. As for the other measures, the total shared information *I*(*X*, *Y* : *Z*) may be significantly less than its maximum (given by target entropy *H*(*Z*)) because the sources need not be able to perfectly explain the target.

Several different formulations of PID exist. While all of the formulations agree on information-theoretic equations constraining the information atoms (P. L. Williams & Beer, 2010), they generally disagree on the definition of the redundant information atom (Barrett, 2015; Griffith, Chong, James, Ellison, & Crutchfield, 2014; Harder, Salge, & Polani, 2013), on the operational interpretation (Bertschinger, Rauh, Olbrich, Jost, & Ay, 2014; Makkeh, Gutknecht, & Wibral, 2021), as well as on whether PID should be symmetric in sources and target (Pica, Piasini, Chicharro, & Panzeri, 2017), among other aspects (see Gutknecht, Wibral, & Makkeh, 2021, for an excellent review on this topic). PID formulations are available for both discrete (Bertschinger et al., 2014; Makkeh et al., 2018; P. L. Williams & Beer, 2010) and continuous-valued (Barrett, 2015; C. Finn & Lizier, 2018b; Ince, 2017; Kay & Ince, 2018; Pakman et al., 2021; Schick-Poland et al., 2021) random variables. In the latter case PID decomposes the differential mutual information, which is somewhat more difficult to interpret, as it reaches infinity for perfectly correlated observables. Application of discrete PID formulations to continuous data is theoretically possible by prior binning of the data. However, binning can incur significant biases in estimation of entropy and related quantities (Paninski, 2003), and therefore is avoided in this work.

Here, we use the continuous formulation of minimal mutual information (MMI) (Barrett, 2015) for continuous data. It must be noted that technically this estimator is only valid if the redundancy is a function purely of the marginal distributions of individual source-target pairs and not the joint distribution. This is the case in the tests employed in this work. For discrete data, we use the discrete formulation of MMI, as well as the BROJA estimator (Makkeh, Theis, & Vicente, 2017; Makkeh et al., 2018) for the Bertschinger interpretation (Bertschinger et al., 2014). Both MMI are implemented by hand with the help of the open-source information-theoretic library NPEET (Steeg, 2013), the BROJA estimator is provided by the open-source Python library IDTxl (Wollstadt, Martínez-Zarzuela, Vicente, Díaz-Pernas, & Wibral, 2014).

### Models

#### Ground truth models.

Here we present two linear models and one quadratic model simulating the target variable Z* as a function of two source variables X* and Y*. For nonsymmetric measures, X* denotes the primary predictor of Z* and Y* denotes the confounding predictor. Each model describes the ground truth variables X*, Y*, and Z* in terms of the latent variables *T*_*x*_, *T*_*y*_, and *T*_*z*_ (Table 2). Each model is designed to exhibit only one of the information atoms (redundant information model **mRed**, unique information model **mUnq**, and synergistic information model **mXOR** given by the XOR operation). The purpose of this choice is to estimate false positive rates in extreme cases. We have designed a continuous-variable and a discrete-variable version of each model. In the continuous case, the latent variables are modeled using standard normal variables, in the discrete case using standard Bernoulli random variables (balanced coin flips). The synergistic model for the continuous case is the sign-XOR function: in terms of magnitudes, all three variables are distributed as standard normal variables, but the sign of Z* is always the product of the signs of X* and Y*.

**Table 2.** Four ground truth models

Shorthand	**mRed**	**mUnq**	**mXOR**	**mSum**	Latent Variables
Continuous Equations	X* = *T*_*x*_	X* = *T*_*x*_	X* = *T*_*x*_	X* = *T*_*x*_	*T*_*x*_ ∼ 𝒩(0, 1)
	Y* = *T*_*x*_	Y* = *T*_*y*_	Y* = *T*_*y*_	Y* = *T*_*y*_	*T*_*y*_ ∼ 𝒩(0, 1)
	Z* = *T*_*x*_	Z* = *T*_*x*_	Z* = |*T*_*z*_|sign(*T*_*x*_)sign(*T*_*y*_)	Z* = *T*_*x*_ + *T*_*y*_	*T*_*z*_ ∼ 𝒩(0, 1)
Discrete Equations	X* = *T*_*x*_	X* = *T*_*x*_	X* = *T*_*x*_	X* = *T*_*x*_	*T*_*x*_ ∼ *Ber*(0.5)
	Y* = *T*_*x*_	Y* = *T*_*y*_	Y* = *T*_*y*_	Y* = *T*_*y*_	*T*_*y*_ ∼ *Ber*(0.5)
	Z* = *T*_*x*_	Z* = *T*_*x*_	Z* = *XOR*(*T*_*x*_, *T*_*y*_)	Z* = *T*_*x*_ + *T*_*y*_	
*U*(*X* → *Z*|*Y*)	0	1	0	?	
*U*(*Y* → *Z*|*X*)	0	0	0	?	
*R*(*X* : *Y* → *Z*)	1	0	0	?	
*S*(*X* : *Y* → *Z*)	0	0	1	?

*Note*. Ground truth variables X*, Y*, and Z* depend linearly on the latent variables *T*, *T*_*x*_, and *T*_*y*_. Each model has a continuous-variable and a discrete-variable version. XOR denotes the exclusive-or logical function. Information atom values of 0 and 1 are given for illustrative purposes, denoting the minimal and maximal values of the corresponding measure. 𝒩 denotes a Gaussian random variable, *Ber* denotes a Bernoulli random variable. Note that the measures disagree on the values of the information atoms in the mSum model.

We also present a composite model mSum, where the target variable Z* is a sum of the two source variables, available both for discrete and continuous variables. Causally, this model can be interpreted as having two unique connections *U*(*X* → *Z*|*Y*) and *U*(*Y* → *Z*|*X*) , which is consistent with the VP measure (Barrett, 2015). However, the PID framework in general also finds significant synergy in this model, and some PID measures also find significant redundancy (Barrett, 2015; Kay & Ince, 2018). Hence, we have only used this model for the validation of the VP measure, as the ground truth values of this model for PID are debatable.

#### Observable models.

The observable variables *X*, *Y*, and *Z* represent the variables actually observed by an experimenter. They are modeled as ground truth variables with added noise terms (Table 3). In the continuous-variable case, the noise terms are modeled as standard normal variables. The parameters *p*_*x*_, *p*_*y*_, and *p*_*z*_ are the noise fractions, which are used to control the fraction of unexplained signal in the observable variables in Equations 2 to 4. Noise fractions are real variables in the range [0, 1]. They linearly interpolate between a pure signal perfectly explained by the ground truth model (*p* = 0), and a 100% noisy signal completely unrelated to the ground truth model (*p* = 1).

**Table 3.** Continuous and discrete observable models

Model type	Observables	Noise fraction	Unexplained variance
Continuous	*X* = (1 − *p*_*x*_)X* + *p*_*x*_*ν*_*x*_	*p*_*x*_ = const	*ν*_*x*_ ∼ 𝒩(0, 1)
	*Y* = (1 − *p*_*y*_)Y* + *p*_*y*_*ν*_*y*_	*p*_*y*_ = const	*ν*_*y*_ ∼ 𝒩(0, 1)
	*Z* = (1 − *p*_*z*_)Z* + *p*_*z*_*ν*_*z*_	*p*_*z*_ = const	*ν*_*z*_ ∼ 𝒩(0, 1)
Discrete	*X* = (1 − *α*_*x*_)X* + *α*_*x*_*ν*_*x*_	*α*_*x*_ ∼ *Ber*(*p*_*x*_)	*ν*_*x*_ ∼ *Ber*(0.5)
	*Y* = (1 − *α*_*y*_)Y* + *α*_*y*_*ν*_*y*_	*α*_*y*_ ∼ *Ber*(*p*_*y*_)	*ν*_*y*_ ∼ *Ber*(0.5)
	*Z* = (1 − *α*_*z*_)Z* + *α*_*z*_*ν*_*z*_	*α*_*z*_ ∼ *Ber*(*p*_*z*_)	*ν*_*z*_ ∼ *Ber*(0.5)

The introduction of noise in the discrete-variable case is slightly more involved because simple addition of two binary variables does not result in a binary variable. We defined the noise terms *ν*_*x*_, *ν*_*y*_, *ν*_*z*_ as standard Bernoulli random variables. We then introduced switching variables *α*_*x*_, *α*_*y*_, *α*_*z*_ modeled by Bernoulli random variables, but this time with varying probability of heads and tails. The observables are obtained by randomly switching between the ground truth variables and the noise variables using the switching variables. The probabilities *p*_*x*_, *p*_*y*_, and *p*_*z*_ of the switching variables are the discrete analogue of noise fractions as they are equal to the mean values of the switching variables.

In the Results section we study the performance of the tripartite measures as function of noise fractions and data size. To do so, datasets of desired size are sampled from the observable models. Since there are three noise fractions, one for each of the three observables, we further reduce the number of parameters by designing three different noise strategies, all of which have only one parameter (Table 4). The noise fractions used in the plots of the main text will refer to this single parameter.

**Table 4.** Three observable models

Noise type	**PureSrc**	**NoisyX**	**Noisy**
Noise fraction	*p*_*x*_ = *p*_*y*_ = 0	*p*_*x*_ = *p*_*z*_ = *ν*	*p*_*x*_ = *p*_*y*_ = *p*_*z*_ = *ν*
	*p*_*z*_ = *ν*	*p*_*y*_ = 0	

*Note*. In the pure sources model (PureSrc), only the target observable *Z* has nonzero noise, the sources were equal to the underlying ground truth variables. In the noisy source *X* model (NoisyX), both the target *Z* and the source *X* observables have noise (equal noise fractions), whereas the source *Y* is kept pure. In the Noisy model, all three observables have added noise (equal noise fractions). Thus, each observable model is parameterized by a single noise fraction *ν*.

### Significance Testing

As a standard method, we employed permutation testing to assess significance of the estimated information atoms. The above-described observable models were used to produce datasets of three observable variables *X*, *Y*, and *Z*. Data size of *N*_*sample*_ = 10,000 was used everywhere, except when the dependence on data size was investigated. For each dataset, the model information atom was computed. The information atom was then recomputed after permuting the data along the target variable *Z*. This approach is more robust than permuting all three variables because the measure implementations in practice may be sensitive to source correlations even in cases where theoretically source correlations should have no impact on the result. This procedure was repeated multiple times (*N*_*test*_ = 10,000), obtaining the distributions of the information atom for original and permuted data. The critical value corresponding to the desired *p* value (0.01) was estimated as the corresponding quantile of the empirical shuffled distribution of the information atom. The critical value was then used to test significance of individual original data points, computing the fraction of significant information atoms. If the computed fraction significantly exceeds the permutation-test *p* value (based on a binomial test, *p* value 0.01), we say that the information atom is above shuffle. However, for clarity of presentation, we did not present the value of the binomial test in the main text figures, as the significance of this test was qualitatively evident from the distribution of sample points with respect to the critical value. The critical value was independently estimated for all experiments, as it may depend on noise fractions and data size.

To provide more conservative critical values in view of the bias that we detected for all measures (see Results), we developed an adjusted testing procedure. To produce conservative critical values, samples were drawn from the corresponding adversarial distribution under the adjusted null hypothesis (see Results), and the corresponding critical value was estimated from the empirical distribution as for the permutation test. The main difference is that the adjusted procedure does not employ data permutation, but directly tests against the worst case scenario model. Such approaches are a standard way of testing estimators over composite null hypothesis, for example, via a likelihood-ratio test (Bickel & Doksum, 2015). Similar procedures are commonly used for testing functional connectivity estimators (Novelli, Wollstadt, Mediano, Wibral, & Lizier, 2019).

## RESULTS

We studied the specificity of information atom estimation in simulated ground truth data, investigating the effect of varying multiple different parameters (see Figure 3C). We tested each of the measures introduced above (PCorr, VP, BROJA PID, and MMI PID) on each of the three ground truth models that were constructed as examples of exactly one underlying information atom (*R*(*X* : *Y* → *Z*), *U*(*X* → *Z*|*Y*), and *S*(*X* : *Y* → *Z*); respective models **mRed**, **mUnq**, and **mXOR**; see Methods). In addition, we tested VP on the **mSum** model. If the estimated information atom type matched the type exhibited by the model, we evaluated true positive and false negative rates. Otherwise, we evaluated false positive and true negative rates. Further, we explored three different observable models (pure source model **PureSrc**, noisy source *X* model **NoisyX**, and impure model for both sources **Noisy**). Finally, we considered both discrete and continuous-variable models, applying the corresponding measures as discussed in the Methods section.

In the following, we first show that the measures mostly perform as expected in the case of idealized PureSrc observables (noise fractions *p*_*x*_ = *p*_*y*_ = 0, *p*_*z*_ ≥ 0), except for the unique model for some measures. We then demonstrate that relaxation of this idealized assumption (*p*_*x*_ ≥ 0, *p*_*y*_ ≥ 0) in discrete data quickly leads to false positives in all measures. In continuous data, we assume a minimal nonzero noise fraction of 1% to avoid information-theoretic measures reaching infinity. We explore in how far the emergence of false positives depends on noise fraction and data size, and compare the results for discrete and continuous-variable estimators. Finally, to reduce the noise-related false positive rates, we propose to test the information atoms using an adjusted null hypothesis. We perform such tests on simulated data for all the above measures using both discrete and continuous data. We find that this testing approach helps to eliminate false positives at the expense of increasing false negatives in weaker results. While in the main text we present only selected model and parameter combinations, all model and parameter combinations are comprehensively shown in the Supporting Information.

### Low False Positive Rate for Pure Source Variables

First, we asked whether measures for estimating tripartite functional relations perform as expected in the idealized pure source scenario, that is, when they have access to the pure (noiseless) values of the source variables but noisy values of the target variable. Note that continuous information-theoretic measures such as MMI are theoretically infinite in case of redundant noiseless sources. Hence, to approximate the pure source scenario, we applied a noise fraction of 1% to the source signals for all continuous metrics.

**Figure 4.**
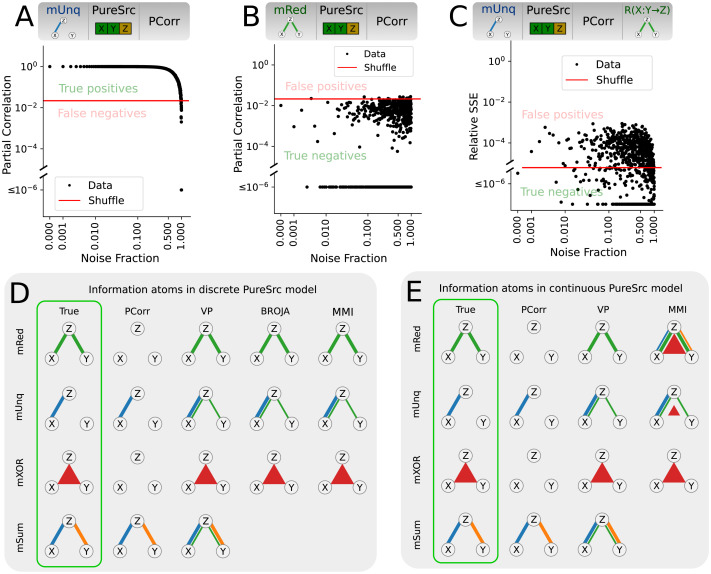
Performance of tripartite analysis measures on PureSrc model. (A) PCorr for the pure source mUnq model. Plotted is the PCorr magnitude as function of noise fraction of the model. Red line is the critical value corresponding to *p* value of 0.01 for permutation testing. For most noise fractions the information atom values are significant, correctly resulting in true positives. (B) Same as A, but for the mRed model. For all noise fractions, most of the estimated information atom values are not significant, correctly resulting in true negatives. (C) Variance partitioning redundant information atom for the pure source mUnq model. In this case, roughly 60% of false positive redundant information atoms are significant, much more than reasonable to expect by chance. (D and E) Sketch of the detected information atoms for noise fraction of 0.25 as function of measure (rows) and ground truth model (columns). Line thickness indicates fraction of significant information atoms (permutation test, *p* value 0.01). Emphasized in green are the theoretically expected results for the underlying ground truth model. All measures correctly identify true positives and true negatives in each model.

For each model and measure, we generated distributions of the information atoms for the model data and shuffled data and used the shuffled results to test the significance of the model results (see Methods). We explored the relation between model and shuffle distributions as a function of the target variable noise fraction. For example, in Figure 4A we plot PCorr for the discrete mUnq model. For most values of noise fractions, PCorr values for the model data (black) exceeds the permutation testing critical value (red), resulting in true positives. For very large noise fractions, the information atom values do not exceed shuffle, resulting in false negatives, which is expected because the functional relation becomes negligible compared to noise. In Figure 4B we plot the PCorr for the discrete mRed model. As *R*(*X* : *Y* → *Z*) is not present in the mUnq model, we expected most of the information atoms estimated from model data not to exceed the critical value, which is exactly what we observe. However, already in pure sources scenario there is one configuration where all measures result in false positives: the *R*(*X* : *Y* → *Z*) information atom for the discrete mUnq model. In Figure 4C we show an example of this effect for VP. Although small in magnitude, the distribution of redundant information atoms found by VP is significantly above the permuted distribution, resulting in a large false positive rate. All other cases are given in Supporting Information Figures S2–S39.

The summary of all test results is sketched in Figure 4D for discrete models and in Figure 4E for continuous models. We find that all measures result in false positive redundant atoms when using the mUnq model already in the pure source case, except for PCorr as it does not compute redundancy. A similar effect is observed with VP given the mSum model. This result is intuitive: whenever the second source correlates with the target by chance, this chance correlation automatically results in redundancy because the first source already correlates with the target; on average, this results in larger redundancy rather than in the case of purely random data. Additionally, continuous-variable MMI results in false positive synergistic information atoms given either the mRed or mUnq model. Our interpretation is that this effect is caused by source noise. As discussed in the Methods section, continuous-variable information-theoretic measures (i.e., MMI) only converge when all variables have some nonzero noise. Further, this effect is not observed in discrete-variable MMI or other measures, and is thus interpreted as false positive.

For other models and information atoms, all measures are significant and specific in discriminating between the different models for a broad range of target variable noise fraction *p*_*z*_. Thus, while some false positives emerge already in this scenario, most measures (except continuous-variable MMI) are largely robust and useful at detecting the true underlying relations.

### High False Positive Rate for Noisy Source Variables

Next, we investigated the scenario when the source variables are not pure (observable models NoisyX and Noisy; see Methods). Here we only present the results for the Noisy model, while the results for the NoisyX model can be found in Supporting Information Figures S2–S39. In summary, results for the NoisyX model are comparable to those for the Noisy model, except for the introduction of large spurious unique information terms in the redundant model, which we address in the Discussion section.

**Figure 5.**
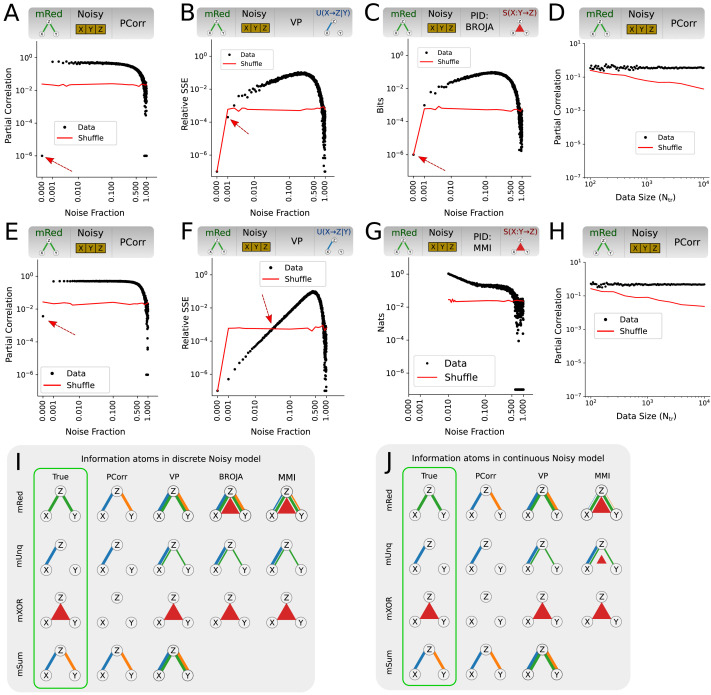
Performance of tripartite analysis measures on model data with noisy source variables. (A) PCorr values as function of the noise fraction using the Noisy discrete mRed model. Red line denotes critical value (*p* value 0.01) based on a permutation test (same in B–D). Red dashed arrow indicates transition from true negatives to false positives (same in B, C). (B) Same as A, but for VP *U*(*X* → *Z*|*Y*). (C) Same as A and B, but for BROJA PID *S*(*X* : *Y* → *Z*). (D) PCorr as function of the data size *N*_*tr*_ for a fixed noise fraction of 0.25 using the Noisy mRed model. (E–H) Same as A–D but for continuous variable models. (I) Sketch of the detected information atoms for Noisy discrete model at noise fraction of 0.25. Line thickness indicates the fraction of significant information atoms (permutation test, *p* value 0.01). (J) Same as I, but for continuous variable models.

In contrast to the PureSrc observable model, the Noisy model resulted in high false positive rates for several additional measures and information atoms (Figure 5), most notably in the mRed model. First, all measures produced spurious unique information atoms in the mRed model, both for discrete and continuous data. While in the Noisy model both unique information atoms had high false positive rates; in the NoisyX model this was the case only for *U*(*Y* → *Z*|*X*) . This suggests that for the unique information atom estimation, additive noise in the confounding (conditional) variable is significantly more dangerous than that in the target or in the primary source. Second, both discrete PID measures (MMI and BROJA) produced spurious synergistic information atoms in the mRed model for the Noisy model (but not the NoisyX model; see Supporting Information Figures S24 and S36). Notably, no significant false positives were observed in the mXOR model.

We thoroughly validated these results. First, we checked the dependence of the results on noise fraction (Figure 5A, B, C, E, F, and G, as well as Supporting Information Figures S2–S39). We found that false positives, such as in the Noisy mRed model, jumped up to 100% for low noise fraction values and remained at 100% for a broad range of noise fractions. For the PCorr measure and BROJA PID *S*(*X* : *Y* → *Z*) atom, noise fractions of already 0.001 were sufficient to cause false positives. For continuous VP *U*(*X* → *Z*|*Y*) the rise of false positive values was not as steep, requiring noise fractions of at least 0.02 to surpass the critical value. Importantly, the largest false positive information atom values were comparable with true positive values, suggesting that at least the weaker true positives cannot be discriminated from the false positives based on their magnitude. Note that the critical value may change with noise fraction, such as in Figure 5B, C, and F. We investigated this effect and found that the estimators for some measures, such as VP and BROJA PID, depend on source correlation for low noise fractions. While this arguably can be interpreted as a minor shortcoming of the individual estimators, it does not affect the results as long as the permutation test only permutes the target and not the sources, as we did here.

Secondly, we checked if the observed false positives were due to insufficient data by studying the asymptotic behaviour of the false positives with increasing data size (Figure 5D and H; Supporting Information Figures S2–S39). We found that the effect sizes of the false positive information atoms actually increased with data size, instead of decreasing, suggesting that the false positives were caused by measure bias, not variance. Note that, for example, in Figure 5D the permutation-based critical value expectedly decreased with data size, whereas the information atom values for model data were comparable for different data sizes. In other measures (see, e.g., redundant information atoms in Supporting Information Figure S15), both the critical value and the model data information atom decreased with data size, but the latter consistently remained above the former for all studied data sizes. This observation suggests that the false positives are due to a bias that cannot be fixed with increasing data size.

We conclude that all the considered measures possess biases in noisy source variable scenarios, emerging even for small noise fractions. Thus, if applied to experimental recordings, permutation testing of significance for all the considered measures can be highly misleading.

### Adjusted Null Hypothesis for Significance Testing of Tripartite Measures With Improved Specificity

To reduce the fraction of false positives in the tripartite measures caused by noise, we developed a testing procedure that accounts for biases in the above measures.

Let *S* be the set of all models for which the true value of the information atom of interest is zero. In this section, when the word “model” is used alone, we mean the combination of both the ground truth and the observable model. Let us first consider the original permutation test in greater detail. Any hypothesis test evaluates the probability that a random sample of a quantity of interest—the test statistic *T*—is as extreme or more extreme than the empirically observed value *T*_*Data*_, given that *T* is distributed according to the null hypothesis *H*_0_. This probability is known as the *p* value *p*.

$$P\left[T>T_{\text{Data}}|H_{0}\right]=p$$ (20)

The null hypothesis is rejected if the *p* value is lower or equal than the significance level *α* of the study, otherwise the test is inconclusive. For a given significance level *α*, there is a critical value of the empirically calculated test statistic Θ which determines if *H*_0_ will be rejected or not. It is computed by solving

$$P\left[T>\Theta |H_{0}\right]=\alpha$$ (21)

for Θ. Thus, if *T*_*Data*_ < Θ, then *p* > *α* and the test fails to reject *H*_0_. Otherwise, if *T*_*Data*_ ≥ Θ, then *p* ≤ *α* and *H*_0_ is rejected.

In a permutation test, the test statistic *T* is the information atom value. The null hypothesis *H*_0_ is that the information atom value comes from the distribution that is produced by a random permutation of the original data. Thus, the permutation test can be performed by computing the critical value Θ from the said *H*_0_ distribution, and then comparing the observed information atom to the critical value. The main problem with this approach is the choice of *H*_0_. It is implicitly assumed that the permutation-induced distribution of the estimated information atom is representative of that distribution for all models in *S*. As shown in the previous section, this assumption does not hold for the considered tripartite measures if the source variables are noisy. The conservative solution designed here is to select the null hypothesis representing the precise scientific question. The adjusted null hypothesis *H*_*adj*_ is that the model that produced the data comes from *S*.

$$H_{adj}:\text{Model}\in S$$ (22)

For simplicity, we used the information atom as a test statistic, although more sophisticated test statistics may yield even better results (see Discussion). If the estimated information atom value exceeds the critical value for *H*_*adj*_, we may reject all models from *S*. If we are to select only one model *M* ∈ *S* as a null hypothesis, we can obtain the critical value Θ_*M*_ for that specific null hypothesis. The critical value Θ_*adv*_ for *H*_*adj*_ is the largest critical value over all of the smaller null hypotheses. Thus, the aim is to find a model in *S* that produces the highest possible critical value, and use that critical value for testing the real data. We will call this model the *adversarial model*.

$$\Theta_{adv}=\max_{M\in S}\Theta_{M}$$ (23)

In summary, in order to determine Θ_*S*_ for a particular information atom of a particular measure, one first needs to do the following four steps:

1. Identify the models that constitute *S*,
2. Find the distribution of the information atom for each of these models,
3. Compute the corresponding critical values, and
4. Select the model with the largest critical value.

Addressing the first step in general would require identifying all linear and nonlinear models that constitute *S*. To the best of our knowledge, results identifying *S* for common PID measures are currently unavailable and may require deep theoretical work specific to each measure, which is beyond the scope of this study. Instead, we restricted our attention to the same model family that was used to create the data, namely, to linear ground truth models with a quadratic coupling term and to additive noise observational models. As a further simplification, we only studied corner case adversarial ground truth models, with only a single information atom present at a time (except for the mSum model). Considering only a single ground truth model and a single observational model for each PID atom at a time enables us to numerically find the noise fraction values that produce the highest adversarial information atom values (worst bias).

**Figure 6.**
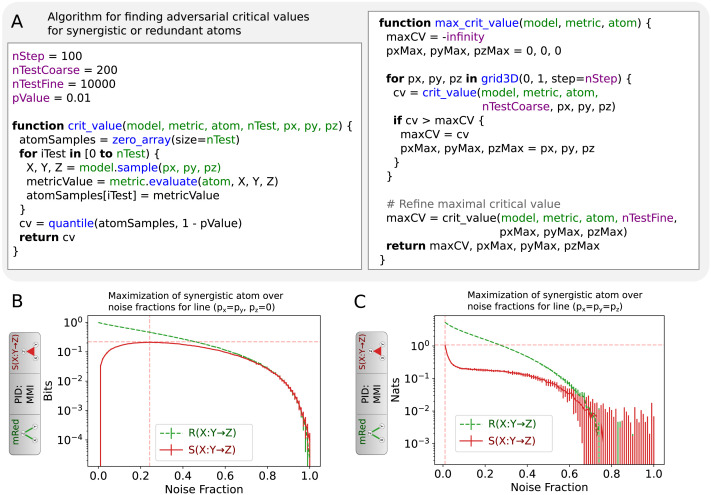
(A) Algorithm to determine the adjusted critical value for redundant and synergistic information atoms. The function *threshold* finds the critical value for a given ground truth and observable model. Function *max_threshold* maximizes the critical value over all observable models. For unique information atoms, the same algorithm would iterate over a line *p*_*x*_ = *p*_*y*_ = *p*_*z*_ instead of a 3D grid. (B) Distribution of false positive *S*(*X* : *Y* → *Z*) (red curve) for discrete MMI PID measure using mRed model as function of noise fraction along the line *p*_*x*_ = *p*_*y*_, *p*_*z*_ = 0. Corresponding true positive *R*(*X* : *Y* → *Z*) values (green) are plotted for comparison. Vertical dashed line denotes the noise fraction with maximal expected false positive *S*(*X* : *Y* → *Z*) value. Horizontal dashed line denotes the 1% upper percentile of *S*(*X* : *Y* → *Z*) at that noise fraction, corresponding to the *p* value 0.01 critical value for *H*_*adj*_. (C) Same as B, but for the continuous variable MMI PID measure.

The distribution of PID atoms under the null hypothesis can either be estimated analytically or numerically. Since analytical distributions for mutual information are available for Gaussian random variables and for asymptotically increasing data sizes (Barnett & Bossomaier, 2012), as well as for discrete distributions (see, e.g., supplementary information for Lizier, 2014), it may be possible to analytically derive the distributions for the atoms of simpler PID measures, such as a Gaussian approximation to MMI PID (Barrett, 2015). However, for more sophisticated measures such as non-Gaussian MMI, BROJA (Bertschinger et al., 2014) and dependence-based PID (Kay & Ince, 2018) analytic results are unlikely. Here, we decided to avoid derivation of analytic distributions and compute the corresponding atom distributions numerically (Figure 6A).

We estimated the information atom distribution under *H*_*adj*_ for each information atom type and each model where that atom is a false positive. For example, for *S*(*X* : *Y* → *Z*), we considered mRed and mUnq as adversarial models, but not mXOR. For each such distribution, we computed the critical value as the upper quantile of the empirical distribution corresponding to the selected *p* value (here 0.01). The resulting critical values for *N*_*tr*_ = 10,000 are plotted in Table 5. First, we observe that the highest false positive unique information atoms are produced using the mRed model, as opposed to mXOR, which does not result in false positives for the measures studied. False positive synergisitc atoms appear only in PID measures, but not in VP, and are also highest in the mRed model. False positive redundant atoms are highest when using the mRed model for all measures except for VP, for which results using mSum are higher than for mRed. The latter suggests that understanding the expected behaviour of PID measures when using mSum may be crucial for improving this testing procedure (see Discussion).

**Table 5.**
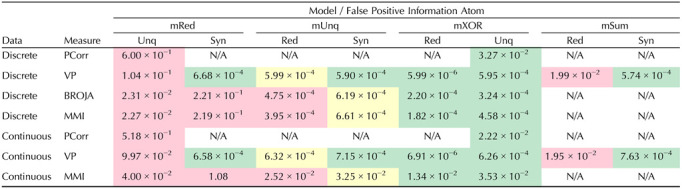
Estimated conservative critical values (CVs) for a given model, measure and information atom, maximized over all noise parameter values

*Note*. In all cases *N*_*tr*_ = 10,000 is assumed. The CVs correspond to the horizontal dashed line in Figure 6B and similar plots (see Supporting Information Figures S40–S45). Green color indicates CVs not significantly different from shuffle, as opposed to yellow and red color. Red color indicates the most conservative CVs across all models for a fixed measure and information atom. Thus, red CVs correspond to purple lines in Figure 7 and Supporting Information Figures S2–S39. For compactness, we used the shorthand notation Unq, Red, Syn for *U*(*X* → *Z*|*Y*), *R*(*X* : *Y* → *Z*), *S*(*X* : *Y* → *Z*) information atoms, respectively. Note that we only test PCorr for specificity to unique information atoms (see Methods). Note that we only use mSum model to test the VP measure (see Methods).

In order to obtain the above critical values, we needed to maximize them over all possible noise models (Figure 6A). First, we discuss the unique information atoms, as the approach is slightly different than for the other two information atoms. In principle, the unique information atom under the mRed model can become arbitrarily prominent if the noise fraction in one of the redundant source variables is arbitrarily larger than in the other. In such situations, the true information atom value is impossible to estimate unambiguously (see Discussion). Instead, here we addressed a subproblem in which all variables have the same noise fractions (*p*_*x*_ = *p*_*y*_ = *p*_*z*_), in other words, using the Noisy model as the adversarial model. This situation can emerge in neuroscience. For example, recordings of multiple neuronal variables may be corrupted by observational noise of the same distribution. Conceptually, the unique information atoms emerge here as false positives because noise corrupts the two redundant source variables in a different way, making them individually significant as predictors of the target variable. This collaborative effect between two noisy sources is useful for improving prediction accuracy of the target, but is certainly undesirable as an estimator of unique information atom significance. We found the maximum likelihood estimate for noise fractions that produced the highest expected information atom value for false positive unique information atoms via a grid search. Noise fraction values between 0 and 1 were split into 100 steps, then for each step the information atom value was resampled 200 times, computing the expected value and the 1% upper percentile critical value. Once the noise fraction resulting in highest critical value was found, the model was resampled 10,000 times for that noise fraction, finding a more precise estimate of the critical value. We refer to this value as the *adjusted critical value* for unique information atoms.

Second, we aimed to correct the bias in redundant and synergistic information atoms. Unlike unique information atoms, false positive synergistic and redundant information atoms did not exhibit unbounded growth with noise fraction asymmetry between source variables. Hence, it was necessary to find the maximum likelihood solution over all combinations of all three noise fraction parameters. We used a grid search with a coarse grid of 10 to 30 steps, discretizing the noise fraction values of each variable between 0 and 1. By visual inspection of this grid, we concluded that the noise fraction dependence of the critical value followed one of four patterns (not shown): noise independent, radially symmetric, dominated by the diagonal *p*_*x*_ = *p*_*y*_ = *p*_*z*_, or dominated by the diagonal with zero source noise *p*_*x*_ = *p*_*y*_, *p*_*z*_ = 0. For the former three, we restricted the search to the diagonal *p*_*x*_ = *p*_*y*_ = *p*_*z*_, whereas for the latter we restrained the search to *p*_*x*_ = *p*_*y*_, *p*_*z*_ = 0. We then proceeded to find the 1% critical values using the same procedure as for the unique information atom.

We found that the distribution of the false positive information atom values changes smoothly with noise fraction, suggesting that the loss from using an overly conservative critical value is minimal for a large range of noise fraction values (see Figure 6B and C and Supporting Information Figures S40–S45). This procedure was repeated for all measures. Further, we explored data sizes in the range of 100–10,000 data points. We found that the critical values experienced a steep decline for data sizes within 100–2,000 data points (up to 3 times), but continued declining rather slowly for values above 2,000, changing by about 3–4% within the range of 2,000–10,000 (Supporting Information Figures S40–S45). Note that the critical value for false positive synergistic information atoms under mRed is problematic for continuous MMI PID, compared to other measures. As can be seen in Figure 6C, it is maximal for the lowest tested noise fraction (0.01) and experiences unbounded growth for noise fractions below that. We address this issue further in the Discussion.

**Figure 7.**
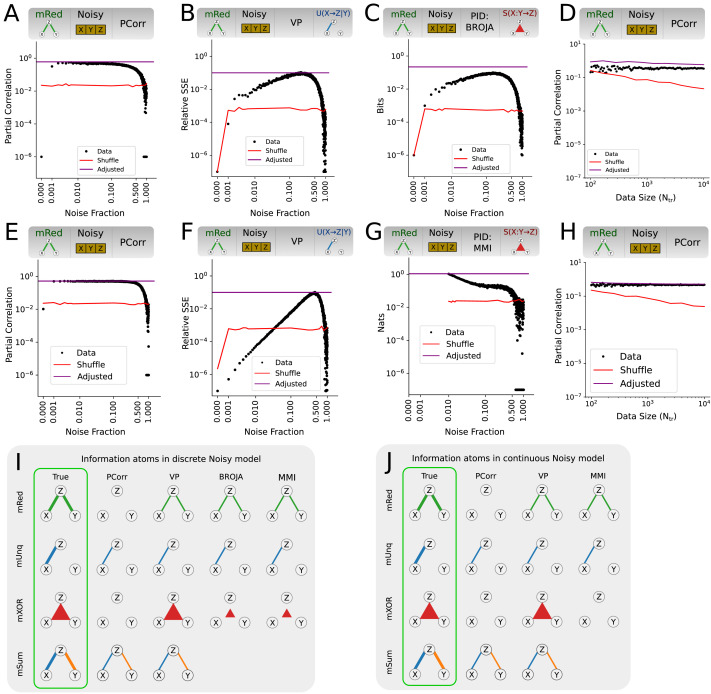
Performance of tripartite analysis measures on model data with noisy source variables (Noisy model), tested against *H*_*adj*_. The conservative test significantly reduces false positives in all measures and information atoms at the expense of increasing false negatives. (A–D) Discrete measure values as function of noise fraction, corresponding exactly to Figure 5A–D. Purple lines denotes the critical values due to *H*_*adj*_. (E–H) Continuous measure values as function of noise fraction, corresponding exactly to Figure 5E–H. Purple lines same as above. (I and J) Sketch of detected discrete-variable (I) and continuous variable (J) information atoms. Same as in Figure 5I–J, except that the fractions of significant information atoms are estimated using the conservative critical values.

We then used the obtained critical values to retest data from all measures and models (Figure 7). We found that our procedure eliminated false positives in all considered measures and models using the Noisy model (Figure 7I and J). Results were qualitatively similar when using the NoisyX model, with the exception of *U*(*Y* → *Z*|*X*) atoms, where the false positives remained for the reasons addressed in the Discussion. Here we present a selection of measures and information atom types (Figure 7A–H) for the Noisy model as function of noise fraction and data size. The plots are the same as the previously shown respective plots (Figure 5A–H), except for an additional horizontal line (purple) denoting the adjusted critical value. All other model and information atom combinations are presented in Supporting Information Figures S2–S39. As a limitation of our approach, stricter critical values also resulted in an increase of false negatives. For example, false negatives in PCorr using the mRed model only appeared for noise fractions above 0.8 when using permutation testing, but started to appear already for noise fraction of 0.5 when using the adjusted testing procedure (Supporting Information Figure S2A). The qualitative behaviour was the same for all true positives when tested against *H*_*adj*_, but transition noise fractions varied. We observed the worst performance in the synergistic information atoms of the continuous MMI PID measure, where true positives were completely eliminated by the adjusted testing procedure. Finally, we inspected the adjusted testing procedure as a function of data size. We plot PCorr using the mRed model and a noise fraction of 0.25 in (Figure 7D and H). The adjusted critical value (purple) changed marginally with data size, decreasing for larger values.

## DISCUSSION

In this work, we studied whether permutation testing of tripartite functional relation estimators is a robust approach for estimating ground truth statistical relations from simulated data in the presence of noise. Several discrete-variable and continuous-variable measures commonly used for such analysis were studied. While such measures are typically assumed to be significant and specific at least in the absence of source noise, we found that this was not always the case, for example, demonstrating that false positive redundant information atoms emerge in a unique-specific model in multiple measures. Furthermore, addition of even small noise fractions to the source signals resulted in dramatic loss of specificity in all measures considered, producing up to 100% false positives. We also demonstrated that false positives become even more significant with increasing data sizes, concluding that this problem cannot be fixed by acquiring more data. As a consequence, if applied to experimental data, permutation testing of these measures could, for example, result in falsely detecting pairwise-specific functional connections in a purely redundant system, which is undesirable and misleading. To address this problem, we designed an alternate testing procedure that accounts for model biases in the presence of noise. Compared to permutation testing, our conservative test consistently eliminated false positives in the studied measures, albeit at the expense of introducing more false negatives with increasing noise fraction. This testing procedure is applicable to any tripartite measure estimating information atoms or related quantities. Researchers are invited to run the simulations in the python code provided (for a given measure and data size) to find the corresponding conservative critical values that then can be applied to testing experimental data.

While we refer to underlying multivariate interactions extensively in this work, we emphasize that our main focus is on the estimation of statistical relations from neuronal data as opposed to the estimation of causal interactions. The set of ground truth models employed in this study is used for illustrative purposes only, as we actually focus on the statistical distributions induced by these ground truth models. Information theory, and thus PID measures, are by design a set of tools for statistical inference and not causal inference. They may be used to narrow down the possible set of causal explanations (Reid et al., 2019), but they are not intended to be tested against specific causal designs. Clearly, joint tripartite statistics does not contain sufficient information to distinguish between all possible causal explanations (Pearl, 2000), especially when unaccounted confounding factors exist that are likely impossible to perfectly control in *in vivo* neuroscience experiments. Instead, we see exploratory analysis as the main application of this work: we propose to use tripartite measures to scan large connectomes for significant unique, redundant, and synergistic effects and to mark interesting emergent relations for future detailed interventional studies.

**Figure 8.**
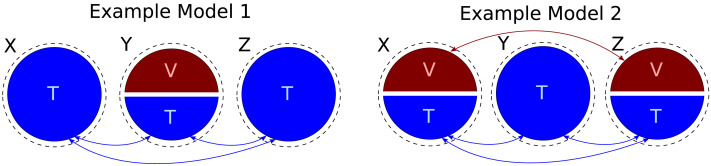
Two different ground truth designs that can produce indistinguishable data. Three populations *X*, *Y*, and *Z* redundantly encode a latent variable *T*. In model 1, the population *Y* additionally encodes another latent variable *V*, whereas in model 2 the second latent variable is additionally encoded by *X* and *Z*.

It is interesting to analyze why the false positives highlighted in this work emerge. Importantly, some of the false positives are not due to shortcomings of individual measures, but rather due to a fundamental ambiguity in data recorded from undercontrolled and/or noisy complex systems. For example, consider the two following scenarios (Figure 8). In the first scenario, population-average observables *X*, *Y*, and *Z* redundantly encode some latent variable *T*. Further, *Y* averages over two different populations of neurons, one that is redundant with *X* and *Z* (encoding *T*) and another one unrelated to *X* or *Z* (called *V*). The constant *α* ∈ [0, 1] determines the relative signal strength of the two neuronal populations in *Y*.

X=T (24)

$$Y=\alpha T+\left(1-\alpha \right)V$$ (25)

Z=T (26)

For *α* between (0, 1) (e.g., *α* = 0.5), redundancy is partially destroyed due to averaging over the two populations in *Y*. In this situation, our analysis will find a nonnegligible *R*(*X*, *Y* → *Z*), as well as a nonnegligible *U*(*X* → *Z*|*Y*). In the second scenario, both *X* and *Z* are averages over two populations of neurons, whereas the population of neurons in *Y* is uniform. The first population in *X* is redundant to the first population in *Z* and to the only population in *Y* (given by the latent variable *T*, same as above). The second population in *X* will be correlated to the second population in *Z*, but unrelated to *Y* (given by the latent variable *V*). Here, the constant *β* ∈ [0, 1] will determine the relative strength of the two neuronal populations in both *X* and *Y*.

$$X=\beta T+\left(1-\beta \right)V$$ (27)

Y=T (28)

$$Z=\beta T+\left(1-\beta \right)V$$ (29)

For appropriate values of the constants, the data distribution sampled from the second model can be statistically indistinguishable from the one sampled from the first model. The difference, however, is that in the second scenario both *U*(*X* → *Z*|*Y*) and *R*(*X*, *Y* → *Z*) meaningfully relate to the underlying neuronal interactions, whereas in the first scenario *U*(*X* → *Z*|*Y*) may be misleading, since *X* and *Z* do not share a stronger connection than, for example, *X* and *Y*. To summarize, this example shows that redundant and unique information atoms can become indistinguishable in cases where the additive noise has a different magnitude in *X*, *Y*, and *Z*. We recommend to take this fact into consideration for future experimental design and interpretation.

Next, we discuss related research regarding functional connectivity (FC) (Friston, 1994) and effective connectivity (EC) (Greicius et al., 2008) and highlight potential implications of our results on estimation of these related measures. Measures of FC and EC aim to estimate a matrix of pairwise connections between variables (also known as functional connectome; E. S. Finn et al., 2015), to test if individual connections are significant, to describe the connectivity matrix by means of integral measures of network neuroscience (Bassett & Sporns, 2017), and to study changes in network connectivity associated, for example, with learning (Bassett et al., 2011; Sych et al., 2019, 2020) or disease (Bullmore & Sporns, 2012). Redundancy is a well-known problem in this field as well. Bayesian approaches (Friston, Harrison, & Penny, 2003) model the posterior distribution of all parameter value combinations and typically bypass the redundancy problem by comparing the relative evidence of a few biologically motivated parameter combinations (Penny, Mattout, & Trujillo-Barreto, 2007). A Frequentist approach to address the problem is to introduce a strict additional criterion on the specificity of inferred connectivity (such as optimal information transfer; Lizier & Rubinov, 2012) and to iteratively prune connections according to such criterion. Comparison of pairwise and pruned connectivity matrices can be used to approximate the range of possible functional networks (Sych et al., 2020).

We conjecture that source noise can negatively affect estimates of time-directed functional connectivity measures, such as transfer entropy. Such measures estimate functional connectivity between a past time point of the source signal and the current time point of the target signal, conditioned on the past time point of the target signal. It relies on the measures similar to those studied here (partial correlation and conditional mutual information) and thus will likely be subject to false positives in the presence of noise. More precisely, a frequent application is the estimation of transfer entropy between two autocorrelated signals that are also correlated at zero lag. The user may be interested in checking if there is significant functional connectivity at small but nonzero lag, independently from the apparent zero-lag functional connectivity. In this case, the activity values of the past of the source, the past of the target, and the current time point of the target will be redundant, and we expect the measure to find no significant lagged functional connections, which may not be the case in the presence of noise. Nevertheless, the worst case scenario for transfer entropy is less dire than that for a general tripartite measure. As past and present of the target come from the same signal, the noise fractions of both of these variables in real data are equal or almost equal, significantly reducing the possible magnitude of false positive unique information atoms. In another study (Sych et al., 2020), we validated the performance of transfer entropy in the presence of noise for simulated neuronal recordings. We found that the measure was able to correctly reject false positives within a range of low noise fractions.

During the last two decades, evidence has accumulated in support of the presence of higher order interactions (tripartite and above) in neuronal populations, including in vivo and in vitro experiments, as well as simulations (for a review see Yu et al., 2011). Two prominent analysis frameworks are Information Geometry (Amari, Nakahara, Wu, & Sakai, 2003) and Maximum Entropy (Schneidman, Berry, Segev, & Bialek, 2006; Shlens et al., 2006). Both frameworks require fitting the data to a multivariate probability distribution from an exponential family. Comparison of models of different complexity (e.g., via maximum likelihood) is used to determine whether the more complex models involving higher order terms are better at explaining the observed data. While we did not explicitly investigate the effects of noise on these frameworks, our current results suggest that these frameworks could be vulnerable to noise, similar to the simpler models studied in our work. Further, synergy and redundancy have been extensively studied in neuroscience by means of the predecessor of PID, namely the Interaction Information (II) and similar measures (see Mediano, Seth, & Barrett, 2018; Timme, Alford, Flecker, & Beggs, 2014, for review). Very recently, this measure has been used to demonstrate synergistic encoding of spatial position by neuron-astrocyte pairs (Curreli, Bonato, Romanzi, Panzeri, & Fellin, 2022). Since II is strongly related to PID and also does not explicitly correct for noise, we would expect the noise-induced false positives to be just as relevant. Finally, we note the emergence of novel approaches to computation of synergistic relations, such as, for example, Intermediate Stochastic Variables (Quax, Har-Shemesh, & Sloot, 2017). Practical application and significance testing of such approaches is a natural extension of this work.

Despite focusing this paper on functional relations between triplets of neuronal signals, our statistical results are general and can see applications outside the scope of neuroscience. Studies of confounding effects, especially by means of partial correlation or partial *r*-squared are common in econometrics (Kenett, Huang, Vodenska, Havlin, & Stanley, 2015; Wang, Xie, & Stanley, 2016), medicine (Buonocore, Zani, Perrone, Caciotti, & Bracci, 1998), genetics (de la Fuente, Bing, Hoeschele, & Mendes, 2004; Reverter & Chan, 2008), neurochemistry (Babinski, Lê, & Séguéla, 1999), psychology (Epskamp & Fried, 2018; D. R. Williams & Rast, 2019), and many other fields. Synergistic effects, among others, have been studied in physical systems (Battiston et al., 2021), ecology (Mayfield & Stouffer, 2017), and sociology (Centola, Becker, Brackbill, & Baronchelli, 2018). Further, earlier in this work we provided an example application where all three variables were of neuronal origin. This choice is purely an interpretation of our statistical results and is done for clarity of presentation purposes. All of our findings are equally applicable to scenarios where all or some of the source and/or target variables are nonneuronal, such as behavioral or sensory variables. For example, see the following:

▪  Functional/effective connectivity between neurons may be investigated as function of an exogenous variable (e.g., treatment, stimulus or behavior) in a mixed behavioral-neuronal experiment with one exogenous source.
▪  Multisensory integration in a cortical or subcortical brain area (Driver & Noesselt, 2008) could be studied as function of auditory and visual stimuli in a mixed behavioral-neuronal experiment with two exogenous sources.
▪  The performance of a participant may be analyzed as function of learning time and reward size in a purely behavioral experiment.

We are aware of a few conceptual difficulties with our approach, which we hope are addressed in future work. First, continuous-variable information-theoretic measures are commonly infinite for zero noise. For all discrete-variable measures and for variance-based continuous-variable metrics (PCorr, VP), it is possible to make a distinction between zero-noise and noisy regimes and demonstrate the emergence of false positives due to this transition. Continuous-variable information-theoretic metrics, such as MMI PID, are only finite for nonzero noise in all variables. Therefore, such a distinction is not possible. Second, PID disagrees with VP on the very concept of synergy. This is well-illustrated by the mSum model. As noted in Equation 22 of Barrett (2015), entropy (and thus mutual information) depends on the logarithm of variance, and thus has different additive properties than variance itself. Namely, variance of the sum $\sigma_{xy}^{2}$ of independent variables is exactly equal to the sum of variances $\sigma_{x}^{2}$ + $\sigma_{y}^{2}$, suggesting two purely unique relations, whereas the joint mutual information *I*(*XY* : *Z*) is greater than the sum of two marginal mutual informations *I*(*X* : *Z*) + *I*(*Y* : *Z*), suggesting the existence of extra synergy. Further, different PID measures disagree on what information atoms should theoretically be present in the sum model and in what quantity. We decided against testing mSum using PID, as we could not converge on a single ground truth in this model. Thus, in this work, we did not consider any of the information atoms emerging under that model as false positives. Future studies disagreeing with this assertion with respect to a given PID measure should be aware that this decision may affect the conservative critical values, which would need to be recomputed taking false positives in the mSum model into account when determining the conservative critical values.

Our results also rely on several simplifying assumptions, some of which are worth improving upon in future studies. First, we computed information atoms using data distribution across trials for a fixed time step. A related question is the study of information atoms across time, for example, in long recordings of resting-state activity. Compared to the former, across-time analysis is complicated by autocorrelation in data. We refer the reader to related recent work addressing autocorrelation effects in functional connectivity estimation (Cliff, Novelli, Fulcher, Shine, & Lizier, 2020; Harris, 2020). Second, we described estimating information atoms using simultaneous source and target data (zero lag). The tripartite measures can be estimated with source signals lagged compared to the target, yielding time-directed information atom estimates (Wibral, Vicente, & Lizier, 2014). Zero-lag estimates can also be thought of as time directed, under the assumption that the timescale of signal propagation in the system is faster than a single time step. Importantly, our results apply equivalently to any choice of lag, as selection of arbitrary lags would still result in a three-variable empirical distribution. For further reference on interpretation of lagged estimators, see Wibral et al. (2014). Third, we used a linear model with a quadratic coupling term and a Gaussian additive noise term. It will be interesting to verify if our results hold for more complex nonlinear ground truth models, nonadditive (e.g., multiplicative) noise, and non-Gaussian (e.g., log-normal) noise distributions. Fourth, our testing procedure relies on several assumptions and simplifications. We assume that false positives are worse than false negatives in exploratory neuroscientific research, since a false detection of a functional relation presumably is more misleading than missing a weaker real relation. Our testing procedure can be made more robust by considering other potential adversarial models, such as nonlinear models of higher order or quadratic models with mixed terms. Sensitivity of our testing procedure can also be improved, reducing the number of false negatives while preserving sensitivity. This is due to the observation that not all of the combinations of information atoms are possible, as they generally depend on each other. For example, the maximal value of the false positive *S*(*X* : *Y* → *Z*) for discrete MMI PID using the mRed model depends on the true value of the *R*(*X* : *Y* → *Z*), as seen in Figure 6B. Instead of testing one information atom at a time, it may be possible to take advantage of the multivariate distribution of all information atoms simultaneously. It would be especially beneficial to apply such corrections to continuous-valued PID measures (see Figure 6C), as there the current version of conservative testing can completely eliminate the true positives. Finally, application of our validation approach to more advanced measures, such as higher order decompositions (P. L. Williams & Beer, 2010), other continuous information-theoretic estimators (C. Finn & Lizier, 2018a; Ince, 2017; Kay & Ince, 2018; Pakman et al., 2021; Schick-Poland et al., 2021), and symmetric information-theoretic estimators (Pica et al., 2017) should provide insight into practical advantages and challenges of these measures in application to noisy neuronal data.

In this work, we presented several applications of tripartite measures to simulated data and demonstrated their usefulness in inferring more advanced network features than those provided by pairwise functional connectivity estimators. We conclude that statistical concerns of testing such measures can mostly be resolved; hence, we recommend the use of such measures in future experimental and computational literature. Moreover, our work presents an example of how permutation testing of a novel measure can produce misleading results. Given the popularity of permutation testing in neuroscience, we recommend extensive theoretical and numerical validation of novel measures prior to use on experimental data.

## ACKNOWLEDGMENTS

We thank Joseph Lizier, Patricia Wollstadt, and Leonardo Novelli for initial support in using the library IDTxl. We are grateful to Michael Wibral and Abdullah Makkeh for extensive support on theory underlying partial information decomposition, especially in terms of interpretation of results. We thank Peter Rupprecht, Adrian Hoffmann, Christopher Lewis, and many other members of the Helmchen Lab for suggestions on improving the manuscript. Finally, we thank William Huber, Ruben van Bergen, and Frank Harrell for useful suggestions with respect to our questions on the state of the art in statistical analysis.

## SUPPORTING INFORMATION

Supporting information for this article is available at https://doi.org/10.1162/netn_a_00259. All code used for this project is available in the open source GitHub repository at https://github.com/aleksejs-fomins/conservative-tripartite-testing (Fomins, 2022a). Note that this project makes extensive use of another library for general purpose multivariate statistical analysis in neuroscience, developed by the authors during this project: https://github.com/HelmchenLabSoftware/mesostat-dev (Fomins, 2022b).

## AUTHOR CONTRIBUTIONS

Aleksejs Fomins: Conceptualization; Formal analysis; Methodology; Software; Validation; Writing – original draft; Writing – review & editing. Yaroslav Sych: Conceptualization; Supervision; Writing – review & editing. Fritjof Helmchen: Funding acquisition; Project administration; Supervision; Writing – review & editing.

## FUNDING INFORMATION

Fritjof Helmchen, Schweizerischer Nationalfonds zur Förderung der Wissenschaftlichen Forschung (https://dx.doi.org/10.13039/501100001711), Award ID: 310030B_170269. Fritjof Helmchen, H2020 European Research Council (https://dx.doi.org/10.13039/100010663), Award ID: 670757.

## Supplementary Material



## TECHNICAL TERMS

Functional/statistical relation: A relation between two or more variables established solely based on the observed statistics (not to be confused with causal relation).
Tripartite functional relation: A relation involving three parties, e.g., three variables, neurons, or brain areas.
Causal relation: A relation between two or more variables where some of the variables have a direct causal effect on the others.
Unique information: An effect where one predictor shares some information with the target that is not shared by any other predictor or predictor combination.
Multicollinearity/redundancy: An effect where multiple predictors share the same information about the target variable.
Partial correlation: Pearson’s correlation coefficient, which controls for one or more confounding variables.
Variance partitioning: A decomposition of variance of a target variable into parts explained by different predictors.
Partial information decomposition: A decomposition of mutual information between multiple sources and a single target into fundamental information atoms (unique, redundant, and synergistic).
Partial R-squared: Coefficient of partial determination, the amount of variance explained uniquely by a (linear) predictor.
Synergy: An effect where multiple predictors share some information with the target that is not shared by any subset of those predictors.

## Supporting Information

https://github.com/aleksejs-fomins/conservative-tripartite-testing

https://github.com/HelmchenLabSoftware/mesostat-dev

## References

[bib1] Achen, C. H. (1990). What does “explained variance” explain?: Reply. Political Analysis, 2, 173–184. 10.1093/pan/2.1.173

[bib2] Aguirre, G. K., Zarahn, E., & D’Esposito, M. (1998). The variability of human, BOLD hemodynamic responses. NeuroImage, 8(4), 360–369. 10.1006/nimg.1998.0369, 9811554

[bib3] Amari, S., Nakahara, H., Wu, S., & Sakai, Y. (2003). Synchronous firing and higher-order interactions in neuron pool. Neural Computation, 15(1), 127–142. 10.1162/089976603321043720, 12590822

[bib4] Andrews, D. F. (1974). A robust method for multiple linear regression. Technometrics, 16(4), 523–531. 10.1080/00401706.1974.10489233

[bib5] Babinski, K., Lê, K.-T., & Séguéla, P. (1999). Molecular cloning and regional distribution of a human proton receptor subunit with biphasic functional properties. Journal of Neurochemistry, 72(1), 51–57. 10.1046/j.1471-4159.1999.0720051.x, 9886053

[bib6] Barnett, L., & Bossomaier, T. (2012). Transfer entropy as a log-likelihood ratio. Physical Review Letters, 109(13), 138105. 10.1103/PhysRevLett.109.138105, 23030125

[bib7] Barrett, A. B. (2015). Exploration of synergistic and redundant information sharing in static and dynamical gaussian systems. Physical Review E, 91(5), 052802. 10.1103/PhysRevE.91.052802, 26066207

[bib8] Bassett, D. S., & Sporns, O. (2017). Network neuroscience. Nature Neuroscience, 20, 353–364. 10.1038/nn.4502, 28230844 PMC5485642

[bib9] Bassett, D. S., Wymbs, N. F., Porter, M. A., Mucha, P. J., Carlson, J. M., & Grafton, S. T. (2011). Dynamic reconfiguration of human brain networks during learning. Proceedings of the National Academy of Sciences, 108(18), 7641–7646. 10.1073/pnas.1018985108, 21502525 PMC3088578

[bib10] Battiston, F., Amico, E., Barrat, A., Bianconi, G., de Arruda, G. F., Franceschiello, B., … Petri, G. (2021). The physics of higher-order interactions in complex systems. Nature Physics, 17(10), 1093–1098. 10.1038/s41567-021-01371-4

[bib11] Bertschinger, N., Rauh, J., Olbrich, E., Jost, J., & Ay, N. (2014). Quantifying unique information. Entropy, 16(4), 2161–2183. 10.3390/e16042161

[bib12] Bickel, P., & Doksum, K. A. (2015). Mathematical statistics: Basic ideas and selected topics. Boca Raton, FL: CRC Press. 10.1201/b19822

[bib13] Bienhold, C., Boetius, A., & Ramette, A. (2011). The energy–diversity relationship of complex bacterial communities in Arctic deep-sea sediments. The ISME Journal, 6(4), 724–732. 10.1038/ismej.2011.140, 22071347 PMC3309351

[bib14] Borcard, D., Legendre, P., & Drapeau, P. (1992). Partialling out the spatial component of ecological variation. Ecology, 73(3), 1045–1055. 10.2307/1940179

[bib15] Brincat, S. L., & Connor, C. E. (2004). Underlying principles of visual shape selectivity in posterior inferotemporal cortex. Nature Neuroscience, 7(8), 880–886. 10.1038/nn1278, 15235606

[bib16] Bullmore, E., & Sporns, O. (2012). The economy of brain network organization. Nature Reviews Neuroscience, 13(5), 336–349. 10.1038/nrn3214, 22498897

[bib17] Buonocore, G., Zani, S., Perrone, S., Caciotti, B., & Bracci, R. (1998). Intraerythrocyte nonprotein-bound iron and plasma malondialdehyde in the hypoxic newborn. Free Radical Biology and Medicine, 25(7), 766–770. 10.1016/S0891-5849(98)00126-9, 9823541

[bib18] Candadai, M., & Izquierdo, E. J. (2020). Sources of predictive information in dynamical neural networks. Scientific Reports, 10(1), 16901. 10.1038/s41598-020-73380-x, 33037274 PMC7547683

[bib19] Centola, D., Becker, J., Brackbill, D., & Baronchelli, A. (2018). Experimental evidence for tipping points in social convention. Science, 360(6393), 1116–1119. 10.1126/science.aas8827, 29880688

[bib20] Chen, T.-W., Wardill, T. J., Sun, Y., Pulver, S. R., Renninger, S. L., Baohan, A., … Kim, D. S. (2013). Ultrasensitive fluorescent proteins for imaging neuronal activity. Nature, 499(7458), 295–300. 10.1038/nature12354, 23868258 PMC3777791

[bib21] Cheyne, D. O. (2013). MEG studies of sensorimotor rhythms: A review. Experimental Neurology, 245, 27–39. 10.1016/j.expneurol.2012.08.030, 22981841

[bib22] Cliff, O. M., Novelli, L., Fulcher, B. D., Shine, J. M., & Lizier, J. T. (2020). Exact inference of linear dependence between multiple autocorrelated time series. arXiv:2003.03887. 10.48550/arXiv.2003.03887

[bib23] Curreli, S., Bonato, J., Romanzi, S., Panzeri, S., & Fellin, T. (2022). Complementary encoding of spatial information in hippocampal astrocytes. PLoS Biology, 20(3), e3001530. 10.1371/journal.pbio.3001530, 35239646 PMC8893713

[bib24] Daube, C., Giordano, B., Schyns, P., & Ince, R. (2019). Quantitatively comparing predictive models with the partial information decomposition. In 2019 conference on cognitive computational neuroscience. Cognitive Computational Neuroscience. 10.32470/CCN.2019.1142-0

[bib25] Daube, C., Ince, R. A. A., & Gross, J. (2019). Simple acoustic features can explain phoneme-based predictions of cortical responses to speech. Current Biology, 29(12), 1924–1937. 10.1016/j.cub.2019.04.067, 31130454 PMC6584359

[bib26] de Heer, W. A., Huth, A. G., Griffiths, T. L., Gallant, J. L., & Theunissen, F. E. (2017). The hierarchical cortical organization of human speech processing. Journal of Neuroscience, 37(27), 6539–6557. 10.1523/JNEUROSCI.3267-16.2017, 28588065 PMC5511884

[bib27] de la Fuente, A., Bing, N., Hoeschele, I., & Mendes, P. (2004). Discovery of meaningful associations in genomic data using partial correlation coefficients. Bioinformatics, 20(18), 3565–3574. 10.1093/bioinformatics/bth445, 15284096

[bib28] Driver, J., & Noesselt, T. (2008). Multisensory interplay reveals crossmodal influences on ‘sensory-specific’ brain regions, neural responses, and judgments. Neuron, 57(1), 11–23. 10.1016/j.neuron.2007.12.013, 18184561 PMC2427054

[bib29] Eichler, M., Dahlhaus, R., & Sandköhler, J. (2003). Partial correlation analysis for the identification of synaptic connections. Biological Cybernetics, 89(4), 289–302. 10.1007/s00422-003-0400-3, 14605893

[bib30] Epskamp, S., & Fried, E. I. (2018). A tutorial on regularized partial correlation networks. Psychological Methods, 23(4), 617–634. 10.1037/met0000167, 29595293

[bib31] Farrar, D. E., & Glauber, R. R. (1967). Multicollinearity in regression analysis: The problem revisited. The Review of Economics and Statistics, 49(1), 92–107. 10.2307/1937887

[bib32] Finke, C., Ostendorf, F., Martus, P., Braun, M., & Ploner, C. (2008). Inhibition of orienting during a memory-guided saccade task shows a Mexican-hat distribution. Neuroscience, 153(1), 189–195. 10.1016/j.neuroscience.2008.01.053, 18358628

[bib33] Finn, C., & Lizier, J. T. (2018a). Pointwise partial information decomposition using the specificity and ambiguity lattices. Entropy, 20(4), 297. 10.3390/e20040297, 33265388 PMC7512814

[bib34] Finn, C., & Lizier, J. T. (2018b). Probability mass exclusions and the directed components of mutual information. Entropy, 20(11), 826. 10.3390/e20110826, 33266550 PMC7512388

[bib35] Finn, E. S., Shen, X., Scheinost, D., Rosenberg, M. D., Huang, J., Chun, M. M., … Constable, R. T. (2015). Functional connectome fingerprinting: Identifying individuals using patterns of brain connectivity. Nature Neuroscience, 18(11), 1664–1671. 10.1038/nn.4135, 26457551 PMC5008686

[bib36] Fisher, R. (1924). The distribution of the partial correlation coefficient. Metron, 3, 329–332.

[bib37] Fomins, A. (2022a). Conservative-tripartite-testing, GitHub, https://github.com/aleksejs-fomins/conservative-tripartite-testing.

[bib38] Fomins, A. (2022b). Mesostat-dev, GitHub, https://github.com/HelmchenLabSoftware/mesostat-dev.

[bib39] Fransson, P., & Marrelec, G. (2008). The precuneus/posterior cingulate cortex plays a pivotal role in the default mode network: Evidence from a partial correlation network analysis. NeuroImage, 42(3), 1178–1184. 10.1016/j.neuroimage.2008.05.059, 18598773

[bib40] Friston, K. J. (1994). Functional and effective connectivity in neuroimaging: A synthesis. Human Brain Mapping, 2(1–2), 56–78. 10.1002/hbm.460020107

[bib41] Friston, K. J., Harrison, L., & Penny, W. (2003). Dynamic causal modelling. NeuroImage, 19(4), 1273–1302. 10.1016/S1053-8119(03)00202-7, 12948688

[bib42] Fuster, J. M. (1973). Unit activity in prefrontal cortex during delayed-response performance: Neuronal correlates of transient memory. Journal of Neurophysiology, 36(1), 61–78. 10.1152/jn.1973.36.1.61, 4196203

[bib43] Gallero-Salas, Y., Han, S., Sych, Y., Voigt, F. F., Laurenczy, B., Gilad, A., & Helmchen, F. (2021). Sensory and behavioral components of neocortical signal flow in discrimination tasks with short-term memory. Neuron, 109(1), 135–148. 10.1016/j.neuron.2020.10.017, 33159842

[bib44] Gelman, A. (2005). Analysis of variance—Why it is more important than ever. The Annals of Statistics, 33(1), 1–53. 10.1214/009053604000001048

[bib45] Greene, W. (2003). Econometric analysis. Upper Saddle River, NJ: Prentice Hall.

[bib46] Greicius, M. D., Supekar, K., Menon, V., & Dougherty, R. F. (2008). Resting-state functional connectivity reflects structural connectivity in the default mode network. Cerebral Cortex, 19(1), 72–78. 10.1093/cercor/bhn059, 18403396 PMC2605172

[bib47] Griffith, V., Chong, E., James, R., Ellison, C., & Crutchfield, J. (2014). Intersection information based on common randomness. Entropy, 16(4), 1985–2000. 10.3390/e16041985

[bib48] Gutknecht, A. J., Wibral, M., & Makkeh, A. (2021). Bits and pieces: Understanding information decomposition from part-whole relationships and formal logic. Proceedings of the Royal Society A: Mathematical, Physical and Engineering Sciences, 477(2251), 20210110. 10.1098/rspa.2021.0110, 35197799 PMC8261229

[bib49] Harder, M., Salge, C., & Polani, D. (2013). Bivariate measure of redundant information. Physical Review E, 87(1), 012130. 10.1103/PhysRevE.87.012130, 23410306

[bib50] Harris, K. D. (2020). Nonsense correlations in neuroscience. Cold Spring Harbor Laboratory. bioRxiv. 10.1101/2020.11.29.402719

[bib51] Harris, K. D. (2021). A test for partial correlation between repeatedly observed nonstationary nonlinear timeseries. arXiv:2106.07096. 10.48550/arXiv.2106.07096

[bib52] Hausman, J. (2001). Mismeasured variables in econometric analysis: Problems from the right and problems from the left. Journal of Economic Perspectives, 15(4), 57–67. 10.1257/jep.15.4.57

[bib53] Heeger, D. J., & Ress, D. (2002). What does fMRI tell us about neuronal activity? Nature Reviews Neuroscience, 3(2), 142–151. 10.1038/nrn730, 11836522

[bib54] Hennig, J. A., Golub, M. D., Lund, P. J., Sadtler, P. T., Oby, E. R., Quick, K. M., … Chase, S. M. (2018). Constraints on neural redundancy. eLife, 7, e36774. 10.7554/eLife.36774, 30109848 PMC6130976

[bib55] Ince, R. A. A. (2017). The partial entropy decomposition: Decomposing multivariate entropy and mutual information via pointwise common surprisal. arXiv:1702.01591. 10.48550/arXiv.1702.01591

[bib56] Kay, J., & Ince, R. (2018). Exact partial information decompositions for Gaussian systems based on dependency constraints. Entropy, 20(4), 240. 10.3390/e20040240, 33265331 PMC7512755

[bib57] Kenett, D. Y., Huang, X., Vodenska, I., Havlin, S., & Stanley, H. E. (2015). Partial correlation analysis: Applications for financial markets. Quantitative Finance, 15(4), 569–578. 10.1080/14697688.2014.946660

[bib58] Lescroart, M. D., Stansbury, D. E., & Gallant, J. L. (2015). Fourier power, subjective distance, and object categories all provide plausible models of BOLD responses in scene-selective visual areas. Frontiers in Computational Neuroscience, 9, 135. 10.3389/fncom.2015.00135, 26594164 PMC4633487

[bib59] Lizier, J. T. (2014). JIDT: An information-theoretic toolkit for studying the dynamics of complex systems. Frontiers in Robotics and AI, 1, 11. 10.3389/frobt.2014.00011

[bib60] Lizier, J. T., & Rubinov, M. (2012). Multivariate construction of effective computational networks from observational data (Tech. Rep.). Max Planck Institute for Mathematics in the Sciences. https://www.mis.mpg.de/preprints/2012/preprint2012_25.pdf

[bib61] Makkeh, A., Gutknecht, A. J., & Wibral, M. (2021). Introducing a differentiable measure of pointwise shared information. Physical Review E, 103(3), 032149. 10.1103/PhysRevE.103.032149, 33862718

[bib62] Makkeh, A., Theis, D., & Vicente, R. (2017). Bivariate partial information decomposition: The optimization perspective. Entropy, 19(10), 530. 10.3390/e19100530 PMC7512785 33265362

[bib63] Makkeh, A., Theis, D., & Vicente, R. (2018). BROJA-2pid: A robust estimator for bivariate partial information decomposition. Entropy, 20(4), 271. 10.3390/e20040271, 33265362 PMC7512785

[bib64] Marrelec, G., Krainik, A., Duffau, H., Pélégrini-Issac, M., Lehéricy, S., Doyon, J., & Benali, H. (2006). Partial correlation for functional brain interactivity investigation in functional MRI. NeuroImage, 32(1), 228–237. 10.1016/j.neuroimage.2005.12.057, 16777436

[bib65] Mayfield, M. M., & Stouffer, D. B. (2017). Higher-order interactions capture unexplained complexity in diverse communities. Nature Ecology & Evolution, 1(3), 62. 10.1038/s41559-016-0062, 28812740

[bib66] Mediano, P. A. M., Seth, A. B., & Barrett, A. B. (2018). Measuring integrated information: Comparison of candidate measures in theory and simulation. Entropy, 21(1), 17. 10.3390/e21010017, 33266733 PMC7514120

[bib67] Mehler, D. M. A., & Kording, K. P. (2018). The lure of misleading causal statements in functional connectivity research. arXiv:1812.03363. 10.48550/arXiv.1812.03363

[bib68] Merkelbach, S., König, J., & Sittinger, H. (2003). Personality traits in multiple sclerosis (MS) patients with and without fatigue experience. Acta Neurologica Scandinavica, 107(3), 195–201. 10.1034/j.1600-0404.2003.02037.x, 12614312

[bib69] Michel, C. M., & Brunet, D. (2019). EEG source imaging: A practical review of the analysis steps. Frontiers in Neurology, 10, 325. 10.3389/fneur.2019.00325, 31019487 PMC6458265

[bib70] Novelli, L., Wollstadt, P., Mediano, P., Wibral, M., & Lizier, J. T. (2019). Large-scale directed network inference with multivariate transfer entropy and hierarchical statistical testing. Network Neuroscience, 3(3), 827–847. 10.1162/netn_a_00092, 31410382 PMC6663300

[bib71] Nuzzi, D., Pellicoro, M., Angelini, L., Marinazzo, D., & Stramaglia, S. (2020). Synergistic information in a dynamical model implemented on the human structural connectome reveals spatially distinct associations with age. Network Neuroscience, 4(3), 910–924. 10.1162/netn_a_00146, 33615096 PMC7888489

[bib72] økland, R. H., & Eilertsen, O. (1994). Canonical correspondence analysis with variation partitioning: Some comments and an application. Journal of Vegetation Science, 5(1), 117–126. 10.2307/3235645

[bib73] Pakman, A., Nejatbakhsh, A., Gilboa, D., Makkeh, A., Mazzucato, L., Wibral, M., & Schneidman, E. (2021). Estimating the unique information of continuous variables. NeurIPS 2021. PMC9137417 35645551

[bib74] Paninski, L. (2003). Estimation of entropy and mutual information. Neural Computation, 15(6), 1191–1253. 10.1162/089976603321780272

[bib75] Pearl, J. (2000). Causality: Models, reasoning, and inference. Cambridge, UK: Cambridge University Press.

[bib76] Penny, W., Mattout, J., & Trujillo-Barreto, N. (2007). Bayesian model selection and averaging. In K. Friston, J. Ashburner, & W. Penny (Eds.), Statistical parametric mapping: The analysis of functional brain images (pp. 454–467). Amsterdam, the Netherlands: Elsevier. 10.1016/B978-012372560-8/50035-8

[bib77] Perri, C. D., Bahri, M. A., Amico, E., Thibaut, A., Heine, L., Antonopoulos, G., … Laureys, S. (2016). Neural correlates of consciousness in patients who have emerged from a minimally conscious state: A cross-sectional multimodal imaging study. The Lancet Neurology, 15(8), 830–842. 10.1016/S1474-4422(16)00111-3, 27131917

[bib78] Pica, G., Piasini, E., Chicharro, D., & Panzeri, S. (2017). Invariant components of synergy, redundancy, and unique information among three variables. Entropy, 19(9), 451. 10.3390/e19090451

[bib79] Quax, R., Har-Shemesh, O., & Sloot, P. (2017). Quantifying synergistic information using intermediate stochastic variables. Entropy, 19(2), 85. 10.3390/e19020085

[bib80] Reid, A. T., Headley, D. B., Mill, R. D., Sanchez-Romero, R., Uddin, L. Q., Marinazzo, D., … Cole, M. W. (2019). Advancing functional connectivity research from association to causation. Nature Neuroscience, 22(11), 1751–1760. 10.1038/s41593-019-0510-4, 31611705 PMC7289187

[bib81] Reverter, A., & Chan, E. K. F. (2008). Combining partial correlation and an information theory approach to the reversed engineering of gene co-expression networks. Bioinformatics, 24(21), 2491–2497. 10.1093/bioinformatics/btn482, 18784117

[bib82] Schick-Poland, K., Makkeh, A., Gutknecht, A. J., Wollstadt, P., Sturm, A., & Wibral, M. (2021). A partial information decomposition for discrete and continuous variables. arXiv:2106.12393. 10.48550/arXiv.2106.12393

[bib83] Schneidman, E., Berry, M. J., Segev, R., & Bialek, W. (2006). Weak pairwise correlations imply strongly correlated network states in a neural population. Nature, 440(7087), 1007–1012. 10.1038/nature04701, 16625187 PMC1785327

[bib84] Schulz, J. M., Kay, J. W., Bischofberger, J., & Larkum, M. E. (2021). GABA_*B*_ receptor-mediated regulation of dendro-somatic synergy in layer 5 pyramidal neurons. Frontiers in Cellular Neuroscience, 15, 718413. 10.3389/fncel.2021.718413, 34512268 PMC8425515

[bib85] Sherrill, S. P., Timme, N. M., Beggs, J. M., & Newman, E. L. (2021). Partial information decomposition reveals that synergistic neural integration is greater downstream of recurrent information flow in organotypic cortical cultures. PLoS Computational Biology, 17(7), e1009196. 10.1371/journal.pcbi.1009196, 34252081 PMC8297941

[bib86] Shlens, J., Field, G. D., Gauthier, J. L., Grivich, M. I., Petrusca, D., Sher, A., … Chichilnisky, E. J. (2006). The structure of multi-neuron firing patterns in primate retina. Journal of Neuroscience, 26(32), 8254–8266. 10.1523/JNEUROSCI.1282-06.2006, 16899720 PMC6673811

[bib87] Steeg, G. V. (2013). NPEET: Non-parametric entropy estimation toolbox, GitHub, https://github.com/gregversteeg/NPEET.

[bib88] Stephan, K. E., Harrison, L. M., Kiebel, S. J., David, O., Penny, W. D., & Friston, K. J. (2007). Dynamic causal models of neural system dynamics: Current state and future extensions. Journal of Biosciences, 32(1), 129–144. 10.1007/s12038-007-0012-5, 17426386 PMC2636905

[bib89] Stevenson, I. H., & Kording, K. P. (2011). How advances in neural recording affect data analysis. Nature Neuroscience, 14(2), 139–142. 10.1038/nn.2731, 21270781 PMC3410539

[bib90] Sych, Y., Chernysheva, M., Sumanovski, L. T., & Helmchen, F. (2019). High-density multi-fiber photometry for studying large-scale brain circuit dynamics. Nature Methods, 16(6), 553–560. 10.1038/s41592-019-0400-4, 31086339

[bib91] Sych, Y., Fomins, A., Novelli, L., & Helmchen, F. (2020). Mesoscale brain dynamics reorganizes and stabilizes during learning. bioRxiv. 10.1101/2020.07.08.193334

[bib92] Tax, T., Mediano, P., & Shanahan, M. (2017). The partial information decomposition of generative neural network models. Entropy, 19(9), 474. 10.3390/e19090474

[bib93] Timme, N., Alford, W., Flecker, B., & Beggs, J. M. (2014). Synergy, redundancy, and multivariate information measures: An experimentalist’s perspective. Journal of Computational Neuroscience, 36(2), 119–140. 10.1007/s10827-013-0458-4, 23820856

[bib94] Wang, G.-J., Xie, C., & Stanley, H. E. (2016). Correlation structure and evolution of world stock markets: Evidence from pearson and partial correlation-based networks. Computational Economics, 51(3), 607–635. 10.1007/s10614-016-9627-7

[bib95] Wibral, M., Vicente, R., & Lizier, J. T. (Eds.). (2014). Directed information measures in neuroscience. Berlin, Germany: Springer. 10.1007/978-3-642-54474-3

[bib96] Williams, D. R., & Rast, P. (2019). Back to the basics: Rethinking partial correlation network methodology. British Journal of Mathematical and Statistical Psychology, 73(2), 187–212. 10.1111/bmsp.12173, 31206621 PMC8572131

[bib97] Williams, P. L., & Beer, R. D. (2010). Nonnegative decomposition of multivariate information. arXiv:1004.2515. 10.48550/arXiv.1004.2515

[bib98] Wollstadt, P., Martínez-Zarzuela, M., Vicente, R., Díaz-Pernas, F. J., & Wibral, M. (2014). Efficient transfer entropy analysis of non-stationary neural time series. PLoS One, 9(7), e102833. 10.1371/journal.pone.0102833, 25068489 PMC4113280

[bib99] Yu, S., Yang, H., Nakahara, H., Santos, G. S., Nikolic, D., & Plenz, D. (2011). Higher-order interactions characterized in cortical activity. Journal of Neuroscience, 31(48), 17514–17526. 10.1523/JNEUROSCI.3127-11.2011, 22131413 PMC6623824

