# Supplementary material for: Conservative significance testing of tripartite statistical relations in multivariate neural data
Source: Netw Neurosci. 2022 Oct 1;6(4):1243–74. doi: 10.1162/netn_a_00259 (PMC11117094; doi:10.1162/netn_a_00259)
Supplement: Supplementary file 1 [file netn-6-4-1243-s001.pdf]

Conservative Significance Testing of Tripartite Statistical Relations in Multivariate Neural Data
Supplementary Material

Aleksejs Fomins, Yaroslav Sych, Fritjof Helmchen

1 Proofs

1.1 Derivation of the Variance Partitioning Redundancy Atom

While it is possible to construct the redundant atom directly from the quadratic model used in the main text, we found it more numerically robust to use the linear model, ignoring the quadratic term. Consider the following 3 linear models

$$Z_{lin,x}(X) = aX \tag{1}$$
$$Z_{lin,y}(Y) = bY \tag{2}$$
$$Z_{lin,x,y}(X,Y) = aX + bY \tag{3}$$

The sum of squared errors for each of the above models can be written in terms of the information atoms. First, we use the full linear model $Z_{lin,x,y}(X,Y)$ to decompose the total sum of squares SST_Z.

$$SST_Z = SSE_{lin,x,y} + SSR_{lin,x,y} \tag{4}$$

The sum of squares explained by this model is the sum of squares explained uniquely by X, plus the sum of squares explained uniquely by Y, plus the sum of squares explained redundantly by X and Y (redundant atom $R(X:Y:Z)$).

$$SSE_{lin,x,y} = U(X \to Z|Y) + U(Y \to Z|X) + R(X:Y \to Z) + SSR_{lin,x,y} \tag{5}$$

Secondly, we can decompose the total sum of squares using the other two models

$$SST_Z = SSE_{lin,x} + SSR_{lin,x} \tag{6}$$
$$SST_Z = SSE_{lin,y} + SSR_{lin,y} \tag{7}$$

As an aside, note that while the explained and residual sum of squares depend on the model function, the total sum of squares SST_Z is only a property of the target data.

$$SST_Z = \sum_i |z_i|^2 \tag{8}$$

The explained sums of squares will contain the redundant atom and the unique atom corresponding to the parameter variable, but will not contain the unique part not present among the parameters

$$SSE_{lin,x} = U(X \to Z|Y) + R(X:Y \to Z) + SSR_{lin,x,y} \tag{9}$$
$$SSE_{lin,y} = U(Y \to Z|X) + R(X:Y \to Z) + SSR_{lin,x,y} \tag{10}$$

Subtracting eq. (6) and eq. (7) from eq. (4), and solving for the redundant atom yields the following definition

$$R(X:Y \to Z) = SST_Z - (SSR_{lin,x} + SSR_{lin,y} - SSR_{lin,x,y}) \tag{11}$$

2 Figures

Note that the magnitudes of all information atoms in all log-scale plots are cropped to the minimum value of 10^{-6} or 10^{-7}, as can be inferred from the individual plots. The purpose of this cropping is to focus the logarithmic plot on the more important part of larger information atoms, as well as to avoid over-interpretation of numerical noise.

metric<br>model	corr	pcorr	mi	cmi
**red**	1.000	0.027	1.000	0.000
**unq**	1.000	1.000	0.994	0.999
**xor**	0.007	0.001	0.000	0.999
**sum**	0.704	1.000	0.499	0.998

Figure 1: Comparison of Partial Correlation and Conditional Mutual Information. We compute Corr, PCorr, Mutual Information (MI) and Conditional Mutual Information (CMI) for 4 different discrete models: red ($Z = X = Y$), unq ($Z = X$), xor ($Z = X$ XOR Y), sum ($Z = X + Y$). PCorr behaves similarly to CMI for redundant and unique models, suggesting that it is sensitive to unique information atoms and not sensitive to redundant information atoms. PCorr also behaves similarly to CMI for the sum model, namely, PCorr ¿ Corr similarly to CMI ¿ MI, the latter implying that the synergistic information is larger than redundant, further implying positive synergy in this model. The only difference between PCorr and CMI is that PCorr is not sensitive to synergy in XOR model. Thus PCorr is consistent with CMI in measuring the sum of unique and synergistic information atoms, although it is only sensitive to some of the synergistic relations but not all.

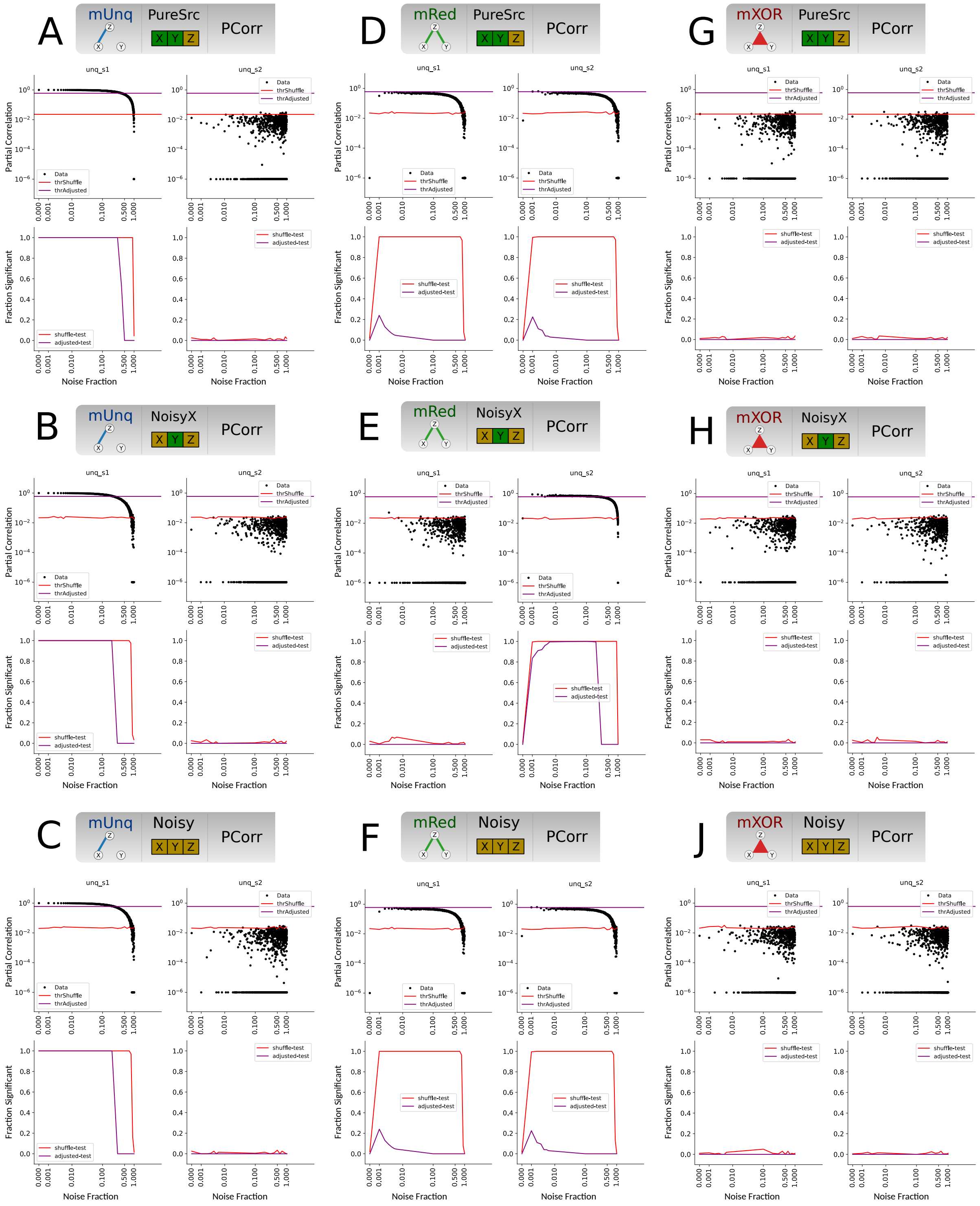

Figure 2: Partial Correlation magnitude (top) and fraction of significant values (bottom) for **discrete** ground truth and observable models, as function of **noise fraction** for $N_{tr} = 10000$. Red line denotes permutation testing critical value (top), and corresponding fraction of significant information atoms (bottom). Purple line denotes the same for the adjusted conservative test. Columns in each figure denote two unique information atoms $U(X \rightarrow Z|Y)$ and $U(Y \rightarrow Z|X)$ respectively.

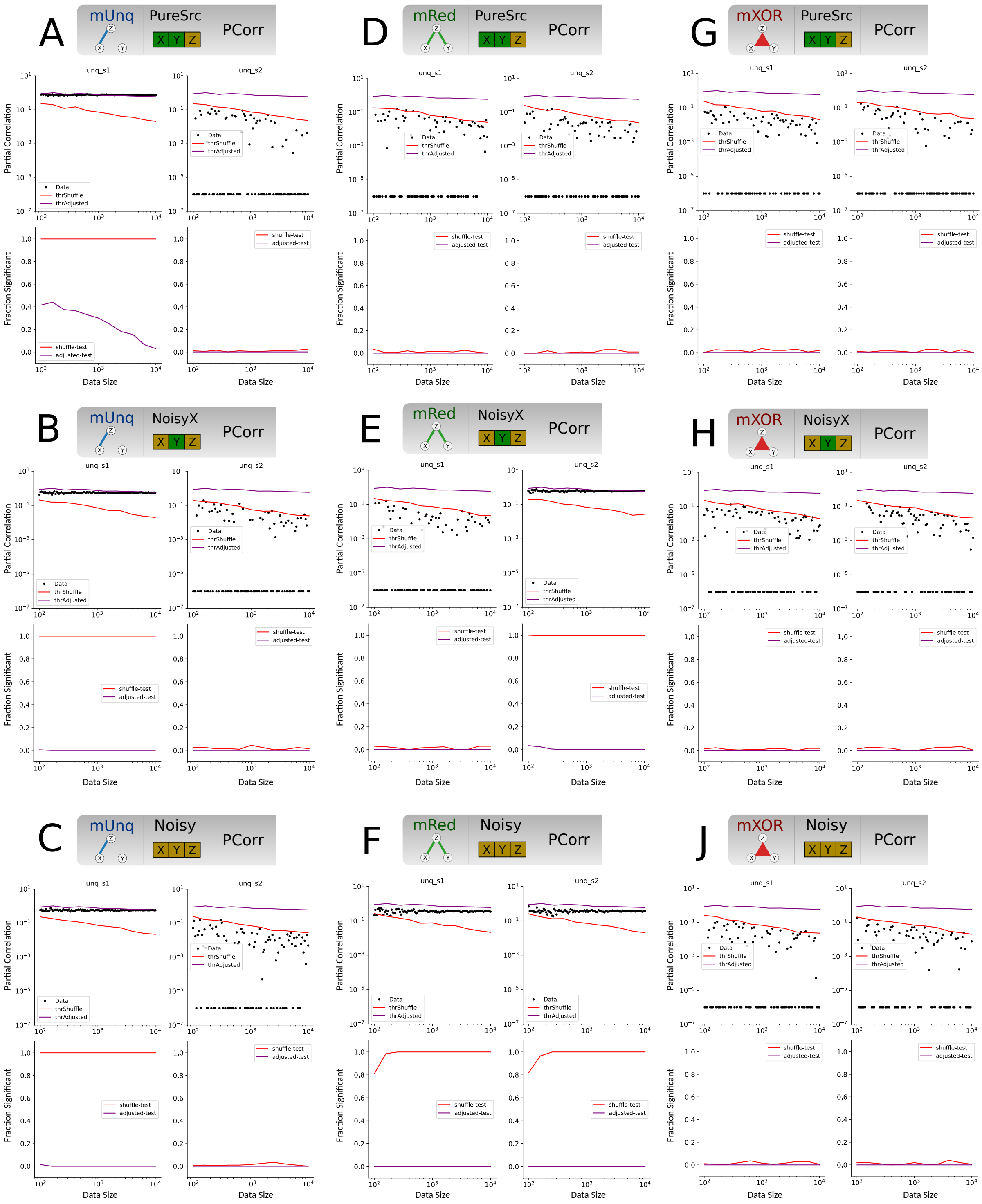

Figure 3: Partial Correlation magnitude (top) and fraction of significant values (bottom) for **discrete** ground truth and observable models, as function of **data size** for fixed noise fraction 0.25. Red line denotes permutation testing critical value (top), and corresponding fraction of significant information atoms (bottom). Purple line denotes the same for the adjusted conservative test. Columns in each figure denote two unique information atoms $U(X \to Z|Y)$ and $U(Y \to Z|X)$ respectively.

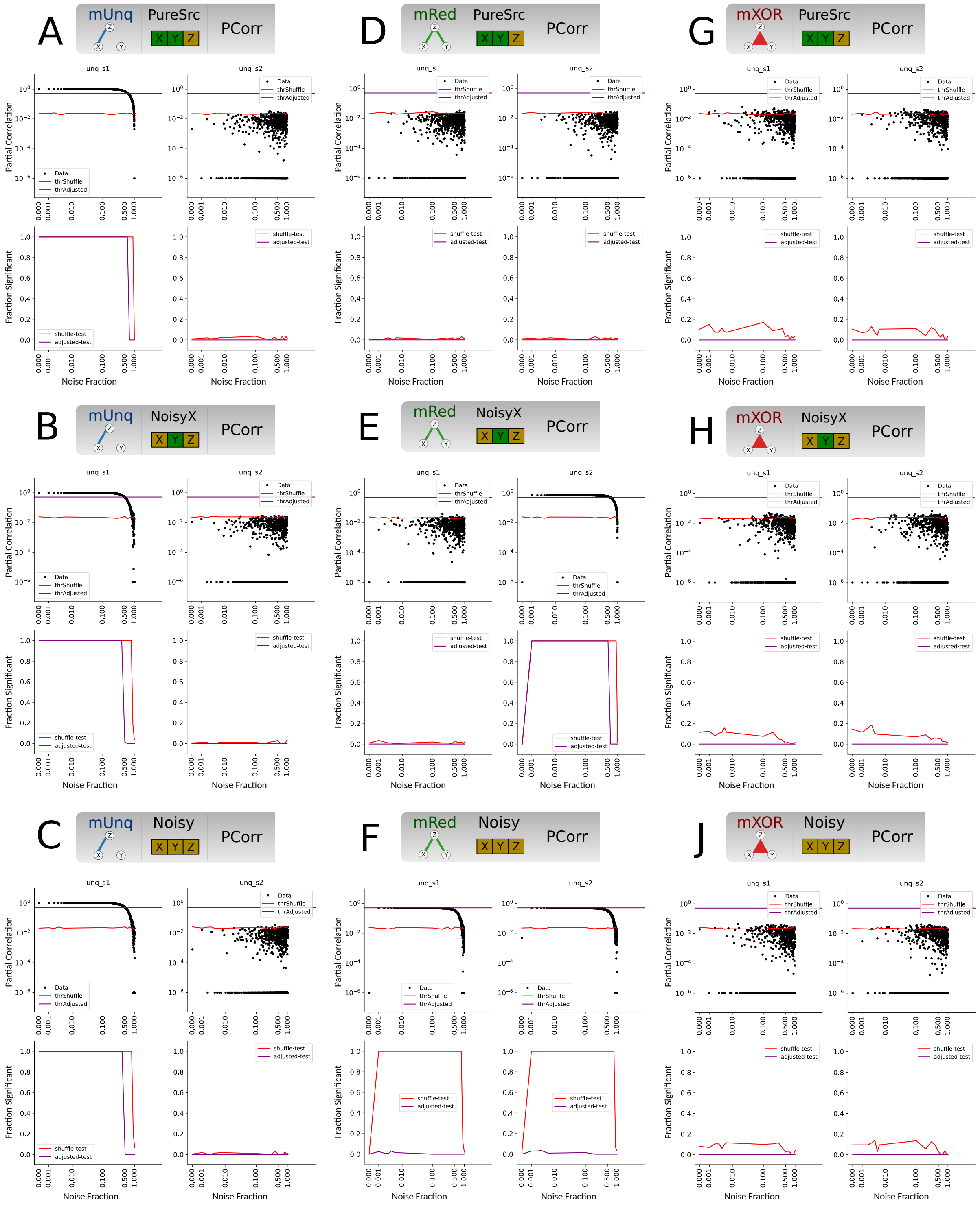

Figure 4: Partial Correlation magnitude (top) and fraction of significant values (bottom) for **continuous** ground truth and observable models, as function of **noise fraction** for $N_{tr} = 10000$. Red line denotes permutation testing critical value (top), and corresponding fraction of significant information atoms (bottom). Purple line denotes the same for the adjusted conservative test. Columns in each figure denote two unique information atoms $U(X \to Z|Y)$ and $U(Y \to Z|X)$ respectively.

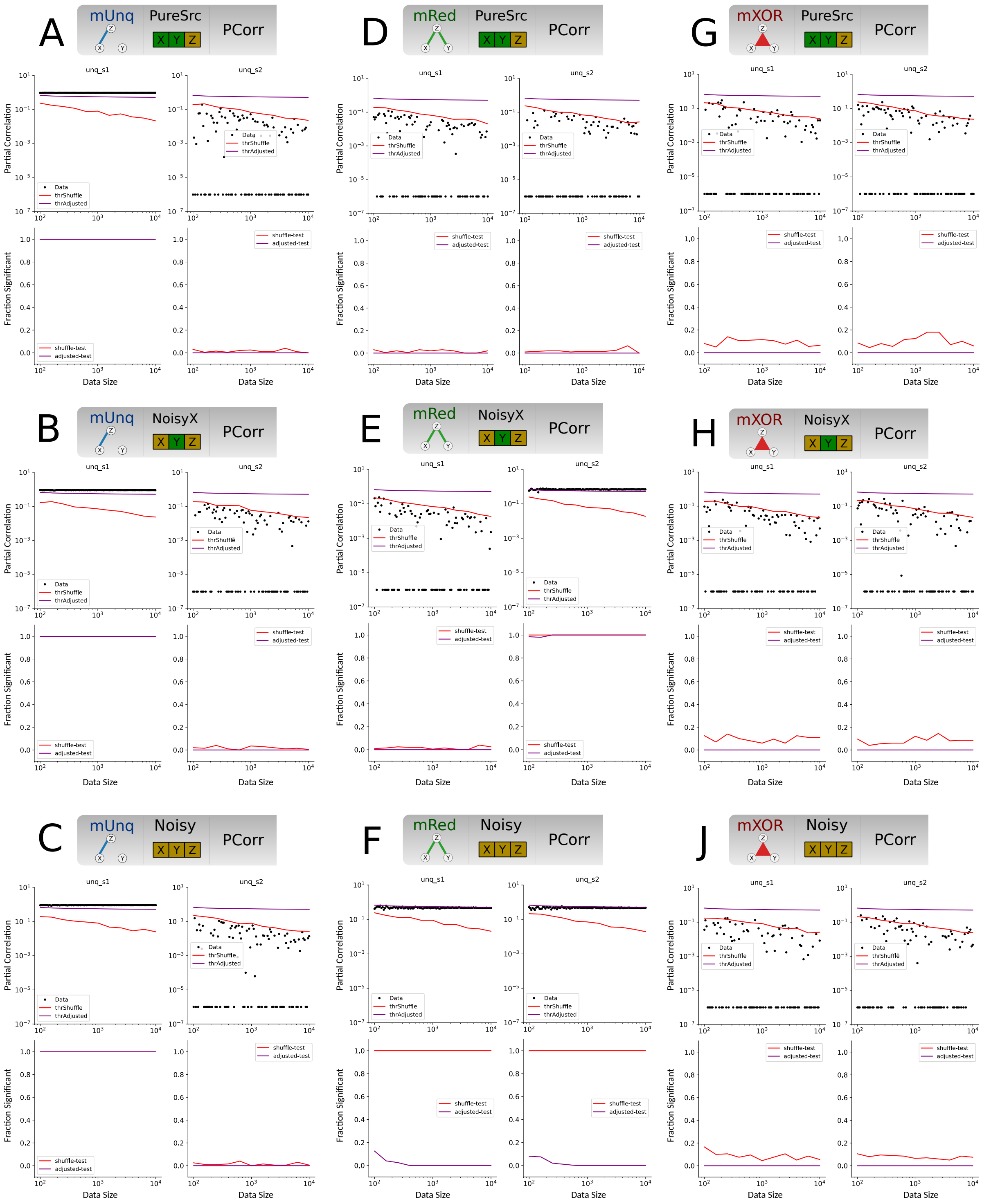

Figure 5: Partial Correlation magnitude (top) and fraction of significant values (bottom) for **continuous** ground truth and observable models, as function of **data size** for fixed noise fraction 0.25. Red line denotes permutation testing critical value (top), and corresponding fraction of significant information atoms (bottom). Purple line denotes the same for the adjusted conservative test. Columns in each figure denote two unique information atoms $U(X \to Z|Y)$ and $U(Y \to Z|X)$ respectively.

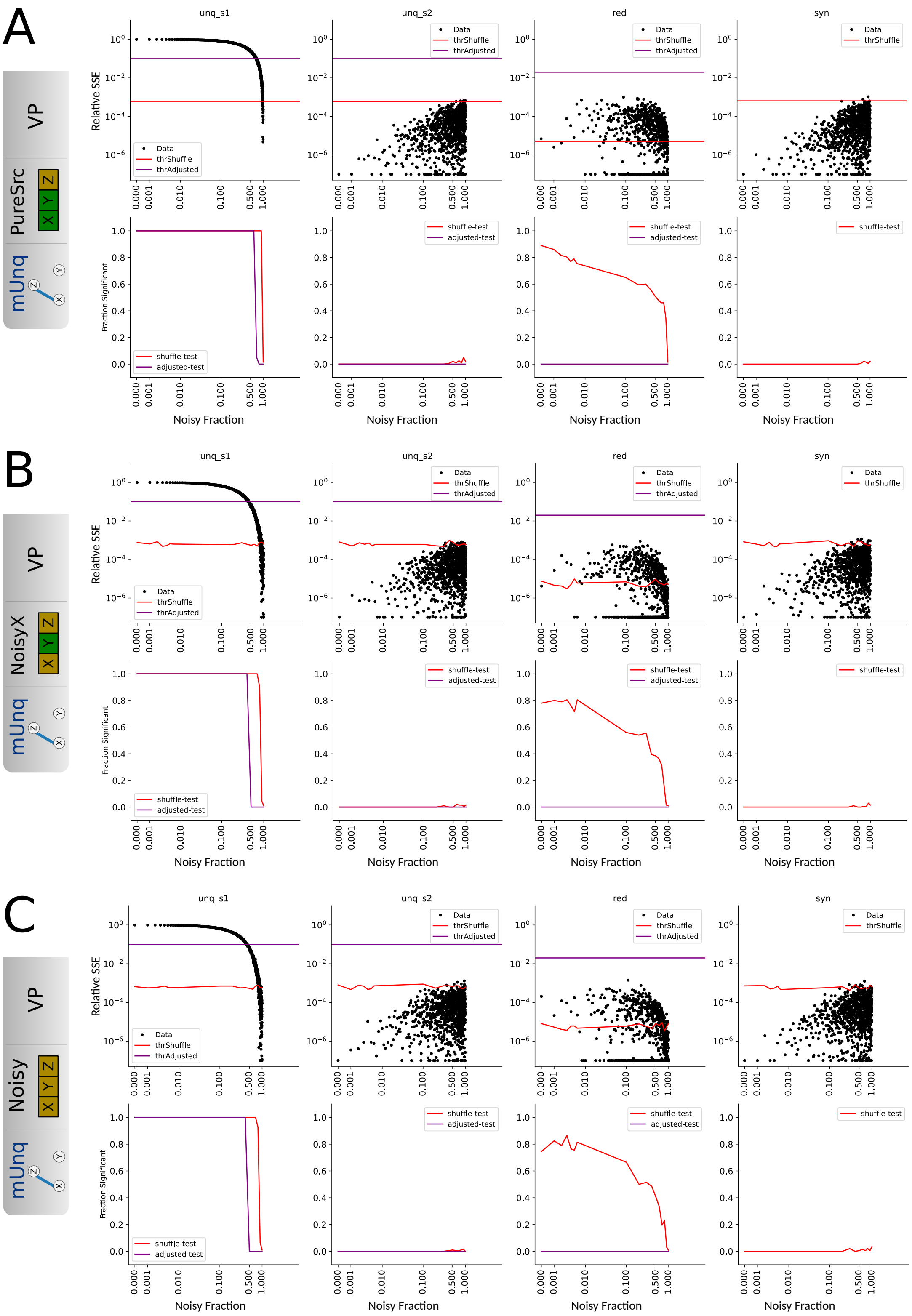

Figure 6: Variance Partitioning magnitude (top) and fraction of significant values (bottom) for **discrete** mUnq and different observable models, as function of **noise fraction** for $N_{tr} = 10000$. Red line denotes permutation testing critical value (top), and corresponding fraction of significant information atoms (bottom). Purple line denotes the same for the adjusted conservative test. Columns in each figure denote information atoms $U(X \to Z|Y)$ and $U(Y \to Z|X)$, $R(X, Y \to Z)$ and $S(X, Y \to Z)$ respectively.

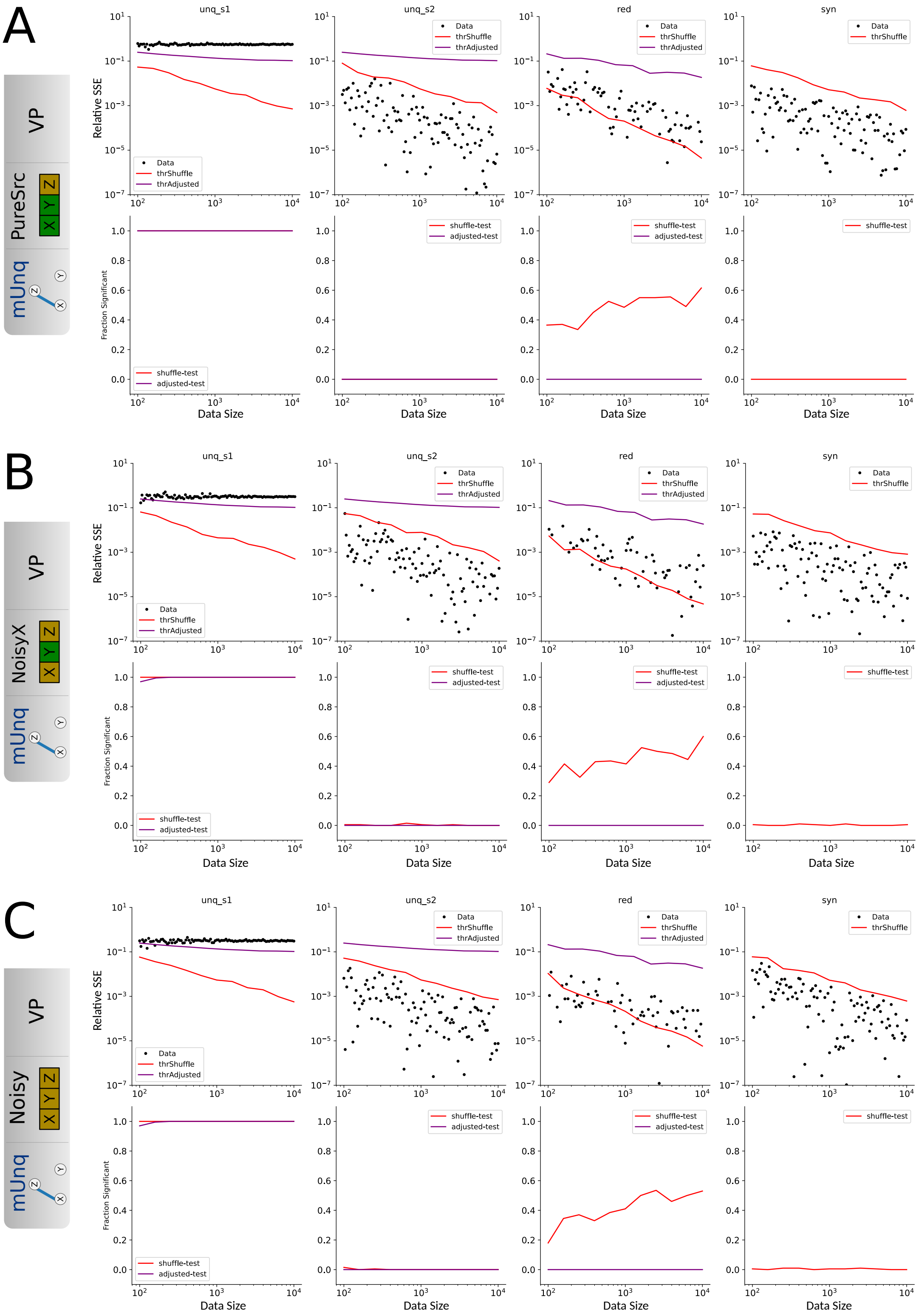

Figure 7: Variance Partitioning magnitude (top) and fraction of significant values (bottom) for **discrete** mUnq and different observable models, as function of **data size** for fixed noise fraction 0.25. Red line denotes permutation testing critical value (top), and corresponding fraction of significant information atoms (bottom). Purple line denotes the same for the adjusted conservative test. Columns in each figure denote information atoms $U(X \to Z|Y)$ and $U(Y \to Z|X)$, $R(X, Y \to Z)$ and $S(X, Y \to Z)$ respectively.

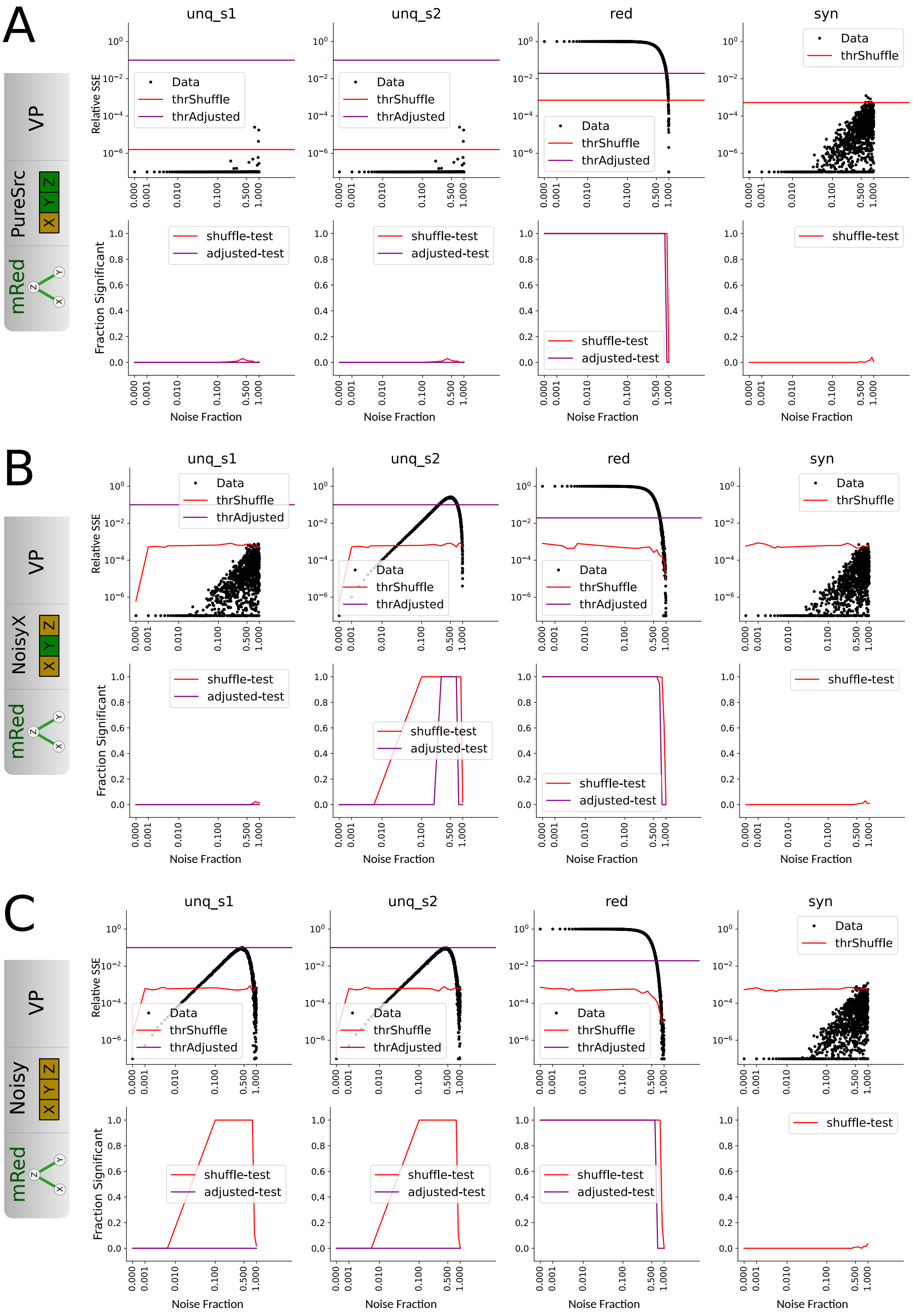

Figure 8: Variance Partitioning magnitude (top) and fraction of significant values (bottom) for **discrete** mRed and different observable models, as function of **noise fraction** for $N_{tr} = 10000$. Red line denotes permutation testing critical value (top), and corresponding fraction of significant information atoms (bottom). Purple line denotes the same for the adjusted conservative test. Columns in each figure denote information atoms $U(X \rightarrow Z|Y)$ and $U(Y \rightarrow Z|X)$, $R(X, Y \rightarrow Z)$ and $S(X, Y \rightarrow Z)$ respectively.

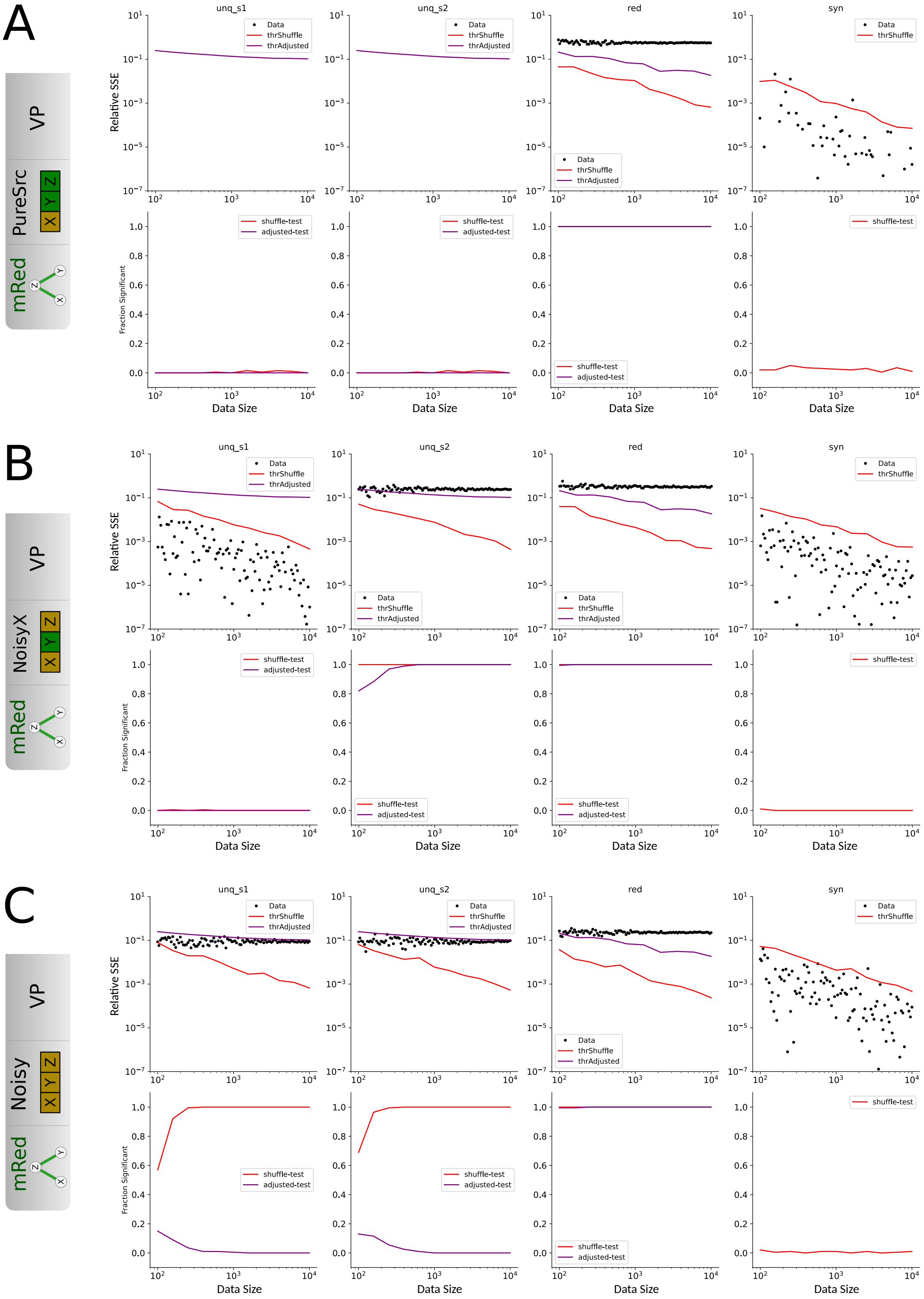

Figure 9: Variance Partitioning magnitude (top) and fraction of significant values (bottom) for **discrete** mRed and different observable models, as function of **data size** for fixed noise fraction 0.25. Red line denotes permutation testing critical value (top), and corresponding fraction of significant information atoms (bottom). Purple line denotes the same for the adjusted conservative test. Columns in each figure denote information atoms $U(X \to Z|Y)$ and $U(Y \to Z|X)$, $R(X, Y \to Z)$ and $S(X, Y \to Z)$ respectively.

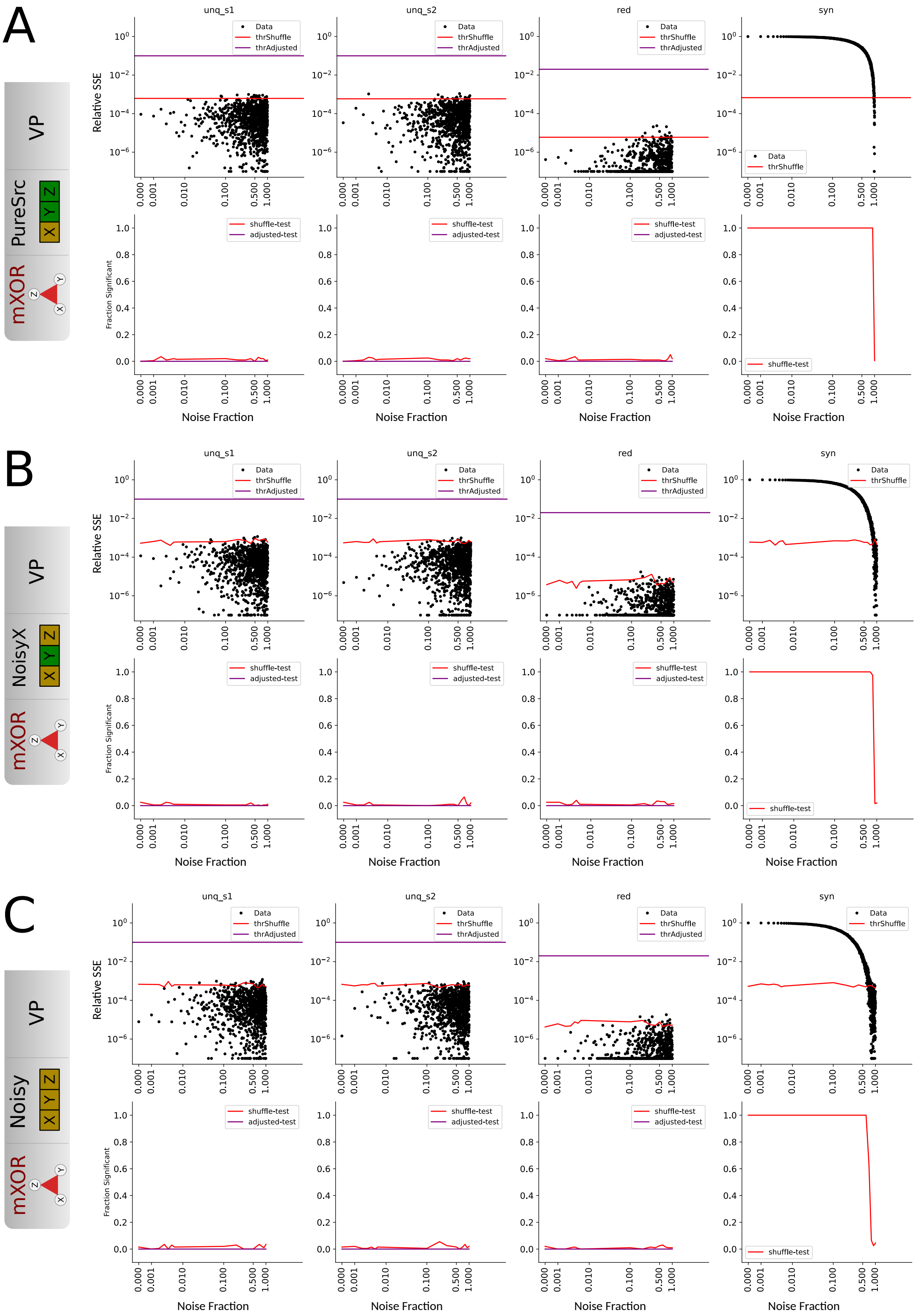

Figure 10: Variance Partitioning magnitude (top) and fraction of significant values (bottom) for **discrete** mXOR and different observable models, as function of **noise fraction** for $N_{tr} = 10000$. Red line denotes permutation testing critical value (top), and corresponding fraction of significant information atoms (bottom). Purple line denotes the same for the adjusted conservative test. Columns in each figure denote information atoms $U(X \rightarrow Z|Y)$ and $U(Y \rightarrow Z|X)$, $R(X, Y \rightarrow Z)$ and $S(X, Y \rightarrow Z)$ respectively.

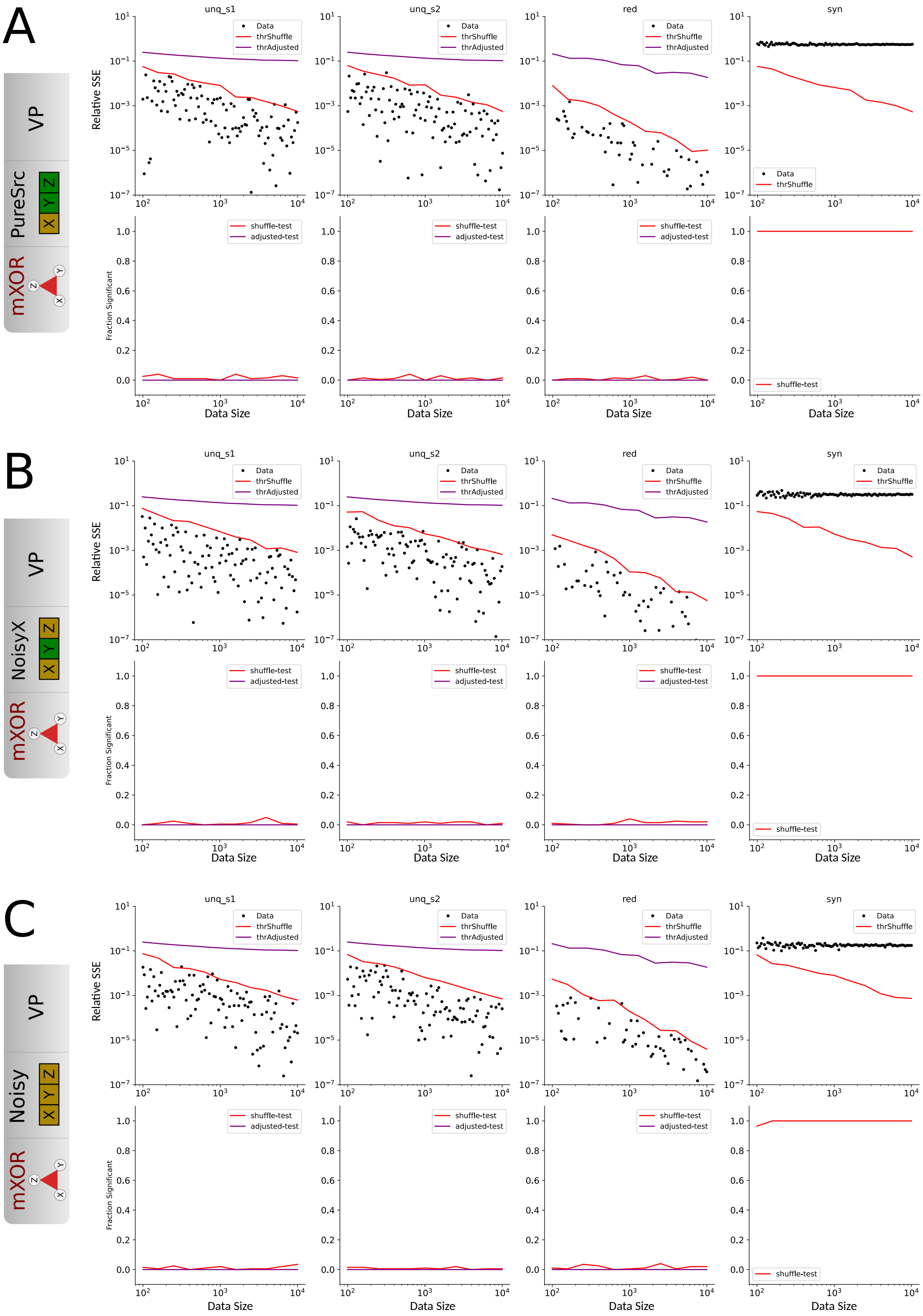

Figure 11: Variance Partitioning magnitude (top) and fraction of significant values (bottom) for **discrete** mXOR and different observable models, as function of **data size** for fixed noise fraction 0.25. Red line denotes permutation testing critical value (top), and corresponding fraction of significant information atoms (bottom). Purple line denotes the same for the adjusted conservative test. Columns in each figure denote information atoms $U(X \to Z|Y)$ and $U(Y \to Z|X)$, $R(X, Y \to Z)$ and $S(X, Y \to Z)$ respectively.

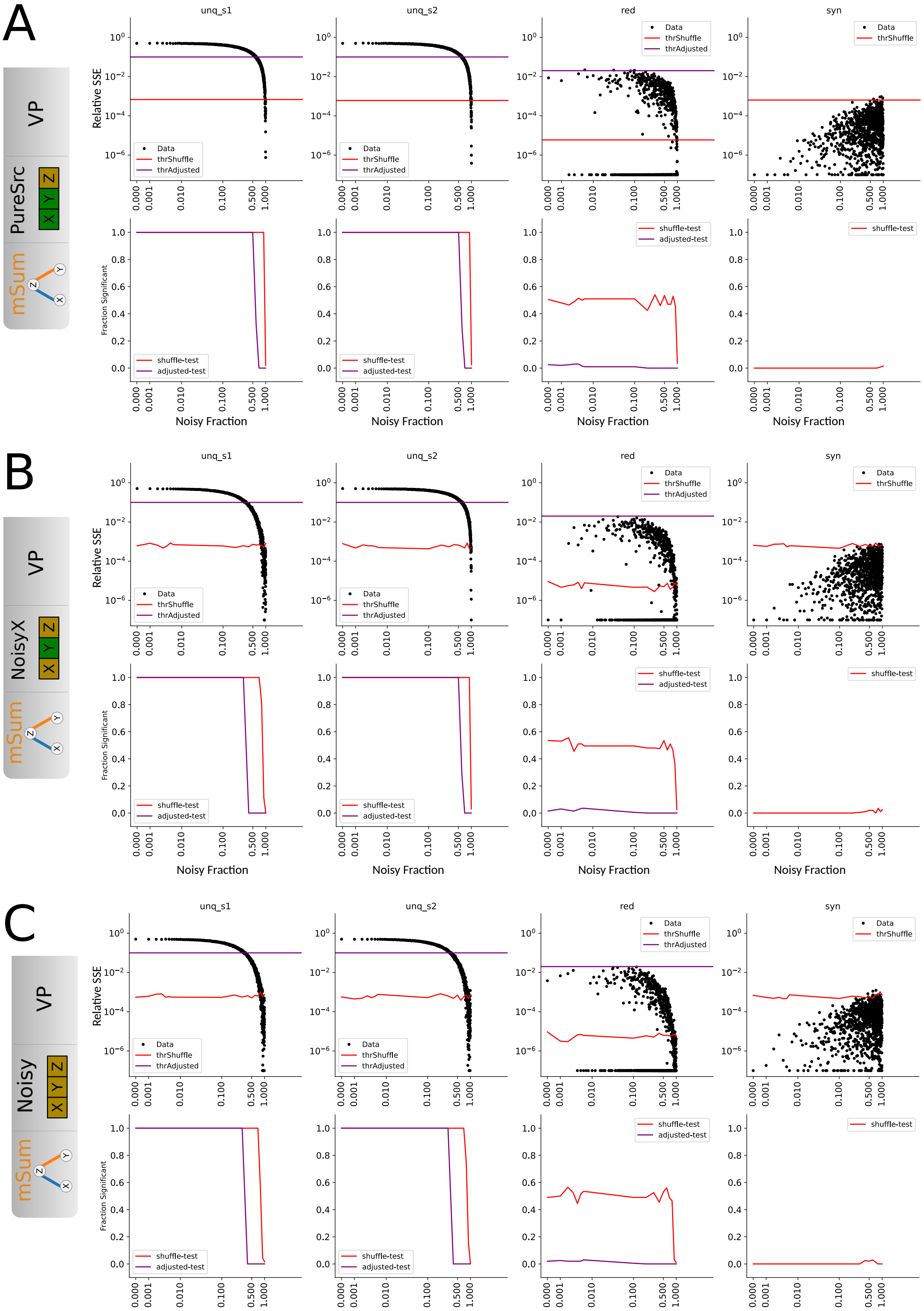

Figure 12: Variance Partitioning magnitude (top) and fraction of significant values (bottom) for **discrete** mSUM and different observable models, as function of **noise fraction** for $N_{tr} = 10000$. Red line denotes permutation testing critical value (top), and corresponding fraction of significant information atoms (bottom). Purple line denotes the same for the adjusted conservative test. Columns in each figure denote information atoms $U(X \rightarrow Z|Y)$ and $U(Y \rightarrow Z|X)$, $R(X, Y \rightarrow Z)$ and $S(X, Y \rightarrow Z)$ respectively.

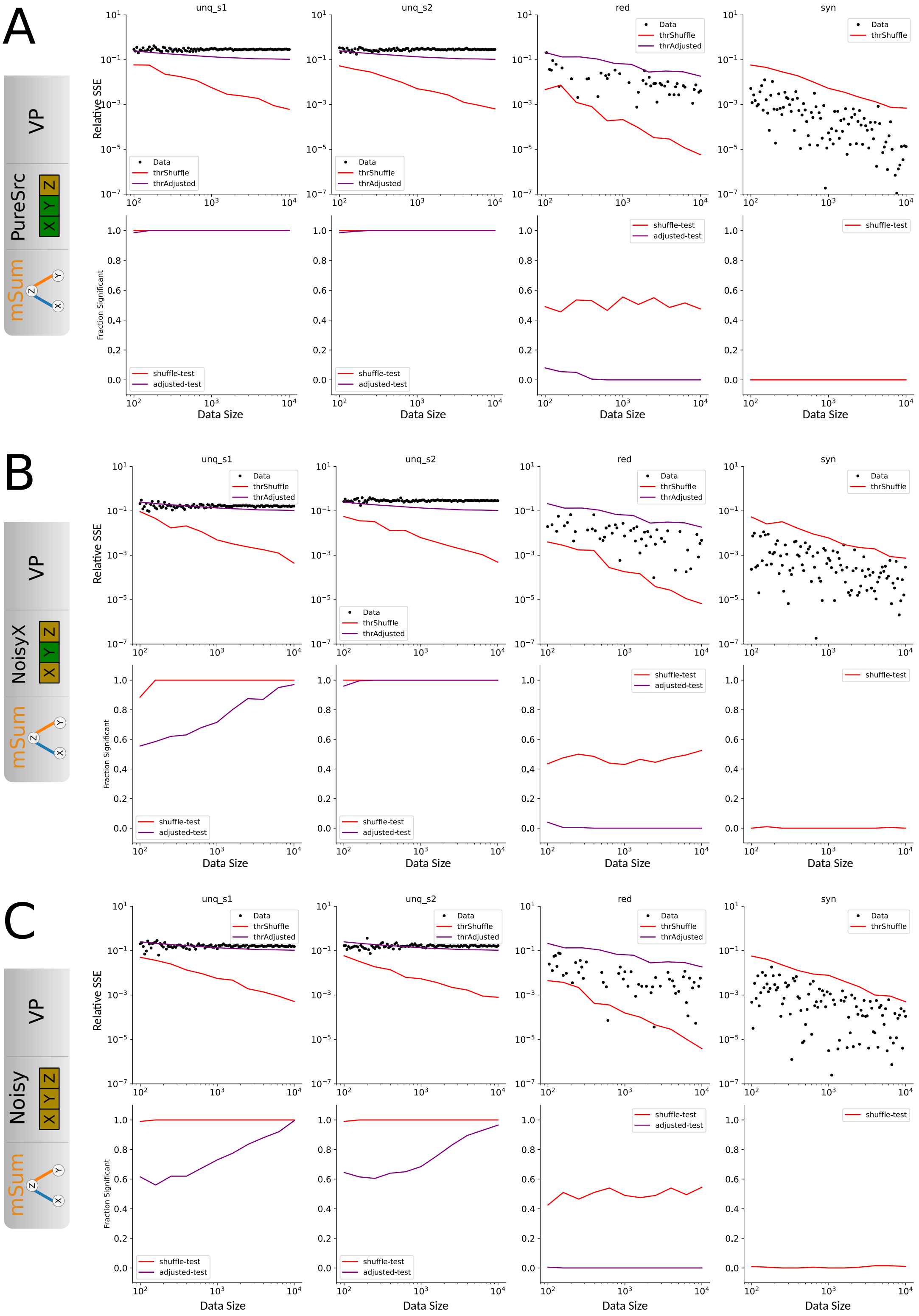

Figure 13: Variance Partitioning magnitude (top) and fraction of significant values (bottom) for **discrete** mSUM and different observable models, as function of **data size for** fixed noise fraction 0.25. Red line denotes permutation testing critical value (top), and corresponding fraction of significant information atoms (bottom). Purple line denotes the same for the adjusted conservative test. Columns in each figure denote information atoms $U(X \to Z|Y)$ and $U(Y \to Z|X)$, $R(X, Y \to Z)$ and $S(X, Y \to Z)$ respectively.

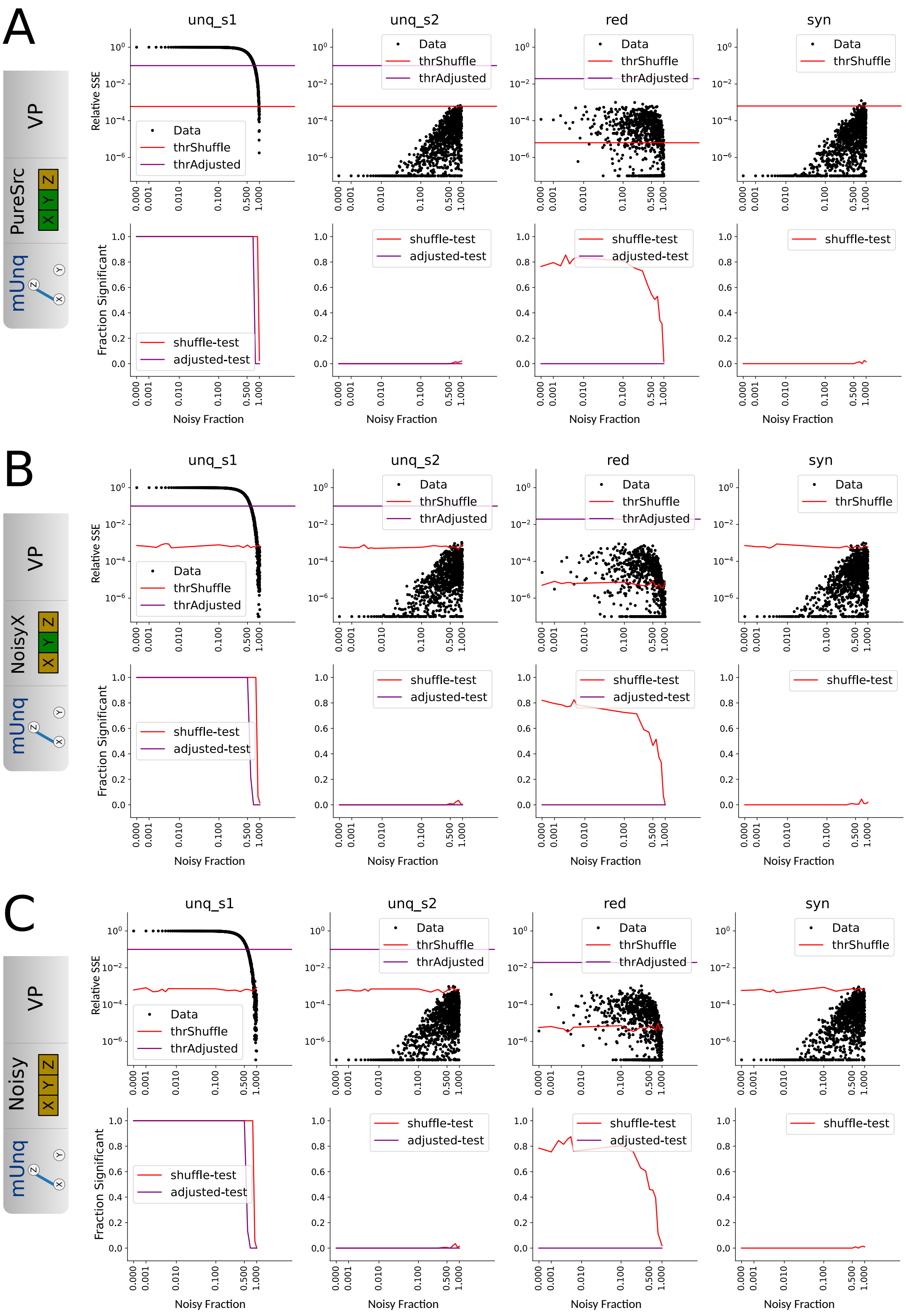

Figure 14: Variance Partitioning magnitude (top) and fraction of significant values (bottom) for **continuous** mUnq and different observable models, as function of **noise fraction** for $N_{tr} = 10000$. Red line denotes permutation testing critical value (top), and corresponding fraction of significant information atoms (bottom). Purple line denotes the same for the adjusted conservative test. Columns in each figure denote information atoms $U(X \to Z|Y)$ and $U(Y \to Z|X)$, $R(X,Y \to Z)$ and $S(X,Y \to Z)$ respectively.

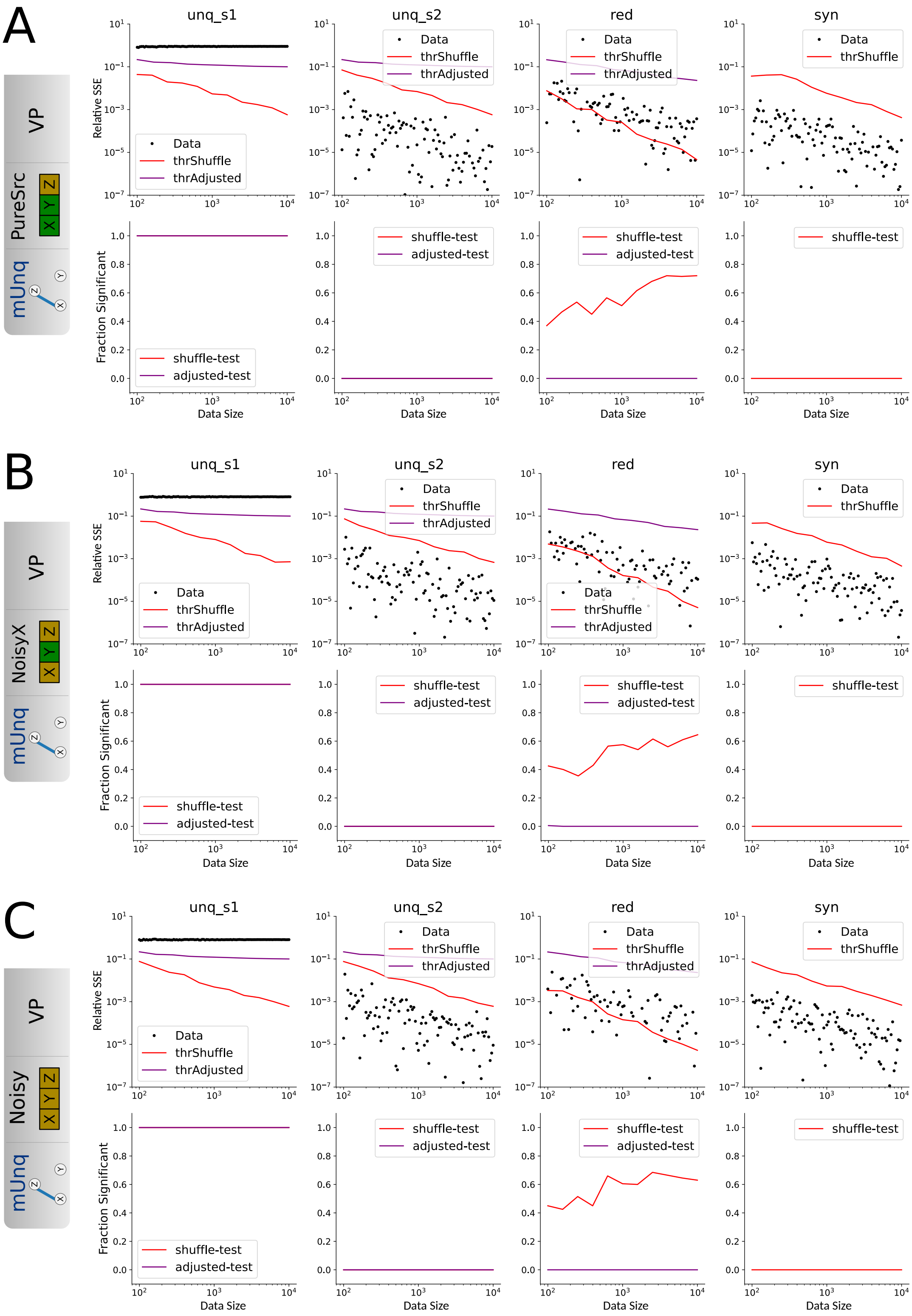

Figure 15: Variance Partitioning magnitude (top) and fraction of significant values (bottom) for **continuous** mUnq and different observable models, as function of **data size** for fixed noise fraction 0.25. Red line denotes permutation testing critical value (top), and corresponding fraction of significant information atoms (bottom). Purple line denotes the same for the adjusted conservative test. Columns in each figure denote information atoms $U(X \to Z|Y)$ and $U(Y \to Z|X)$, $R(X, Y \to Z)$ and $S(X, Y \to Z)$ respectively.

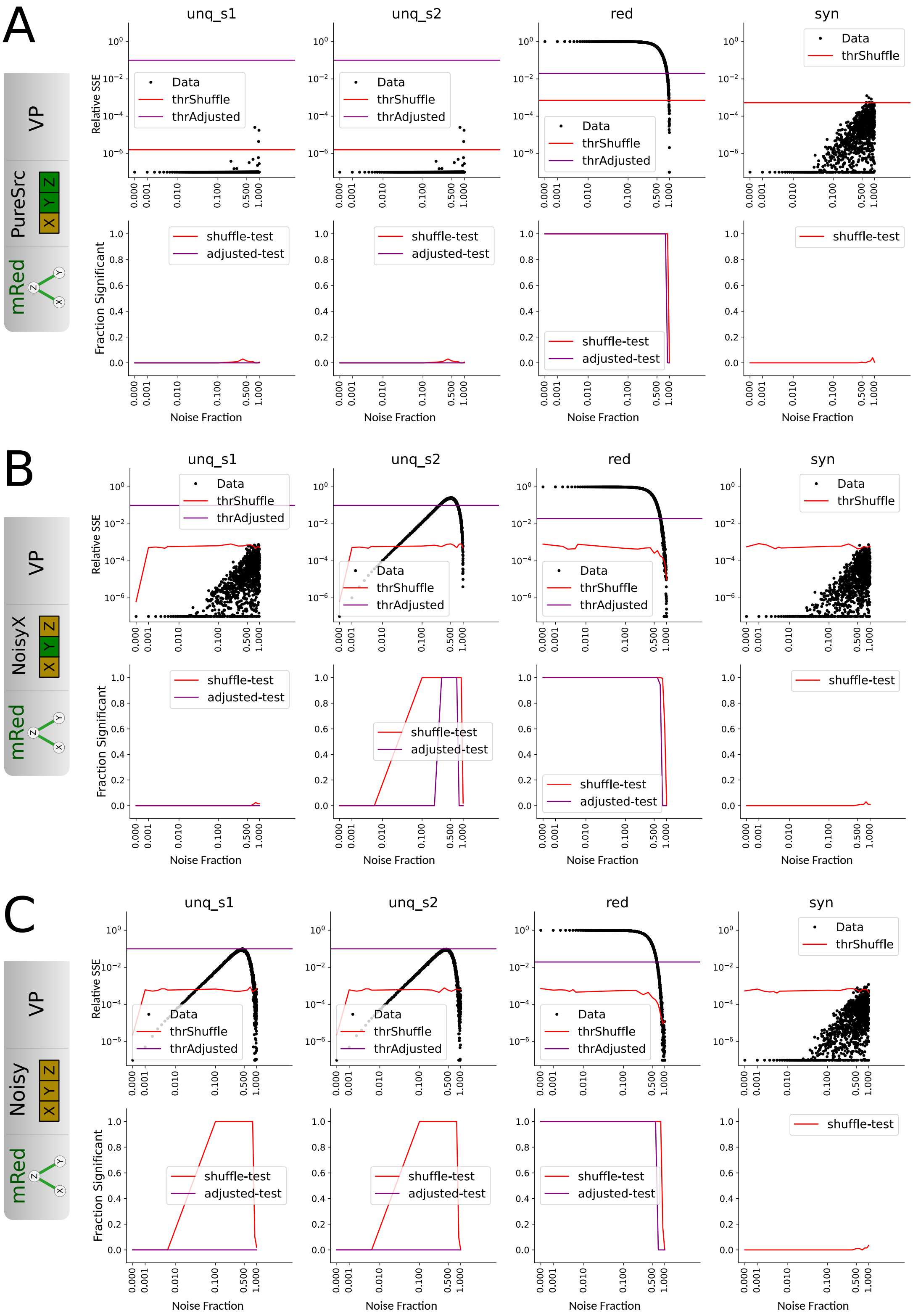

Figure 16: Variance Partitioning magnitude (top) and fraction of significant values (bottom) for **continuous** mRed and different observable models, as function of **noise fraction** for $N_{tr} = 10000$. Red line denotes permutation testing critical value (top), and corresponding fraction of significant information atoms (bottom). Purple line denotes the same for the adjusted conservative test. Columns in each figure denote information atoms $U(X \to Z|Y)$ and $U(Y \to Z|X)$, $R(X, Y \to Z)$ and $S(X, Y \to Z)$ respectively.

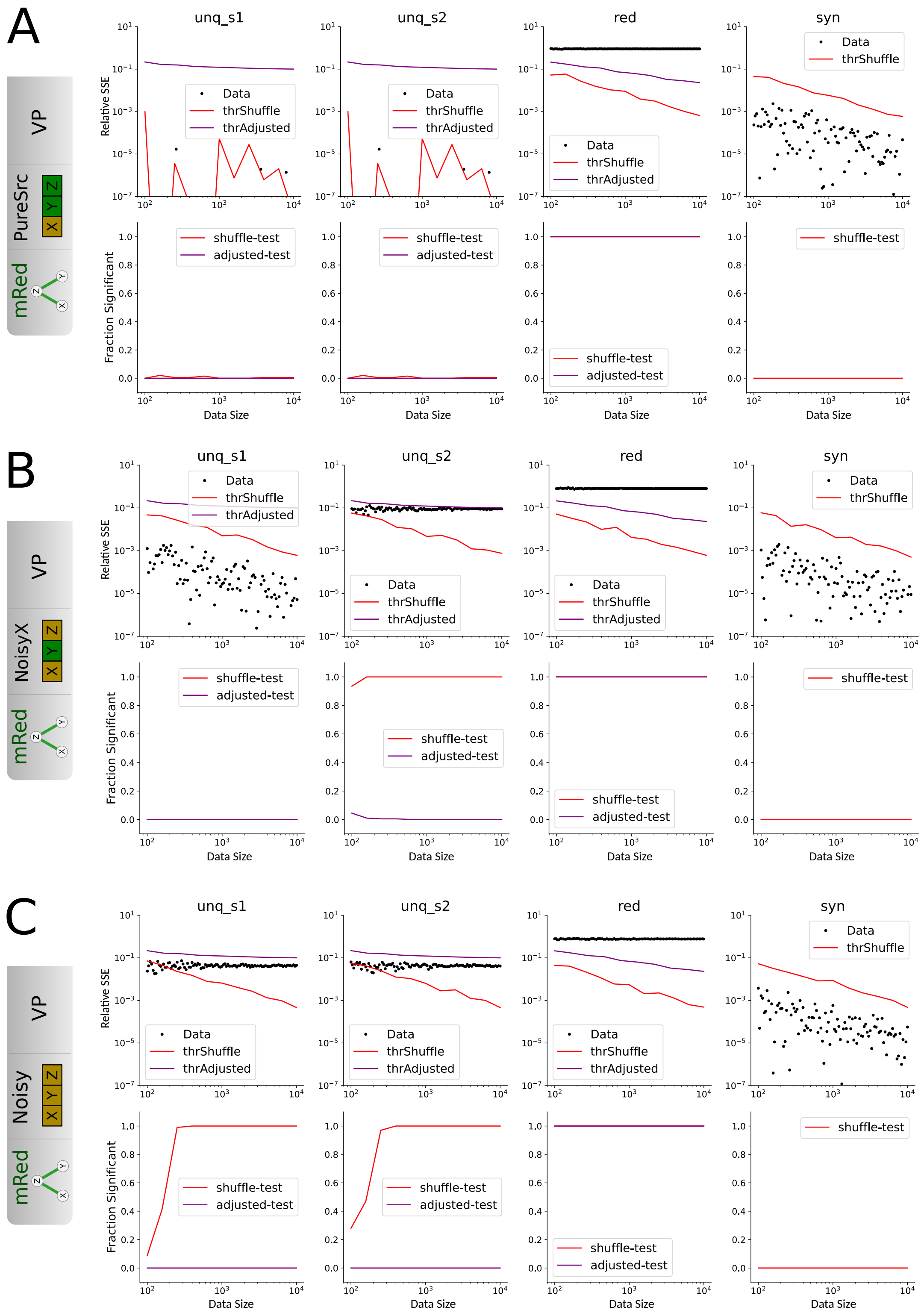

Figure 17: Variance Partitioning magnitude (top) and fraction of significant values (bottom) for **continuous** mRed and different observable models, as function of **data size** for fixed noise fraction 0.25. Red line denotes permutation testing critical value (top), and corresponding fraction of significant information atoms (bottom). Purple line denotes the same for the adjusted conservative test. Columns in each figure denote information atoms $U(X \to Z|Y)$ and $U(Y \to Z|X)$, $R(X, Y \to Z)$ and $S(X, Y \to Z)$ respectively.

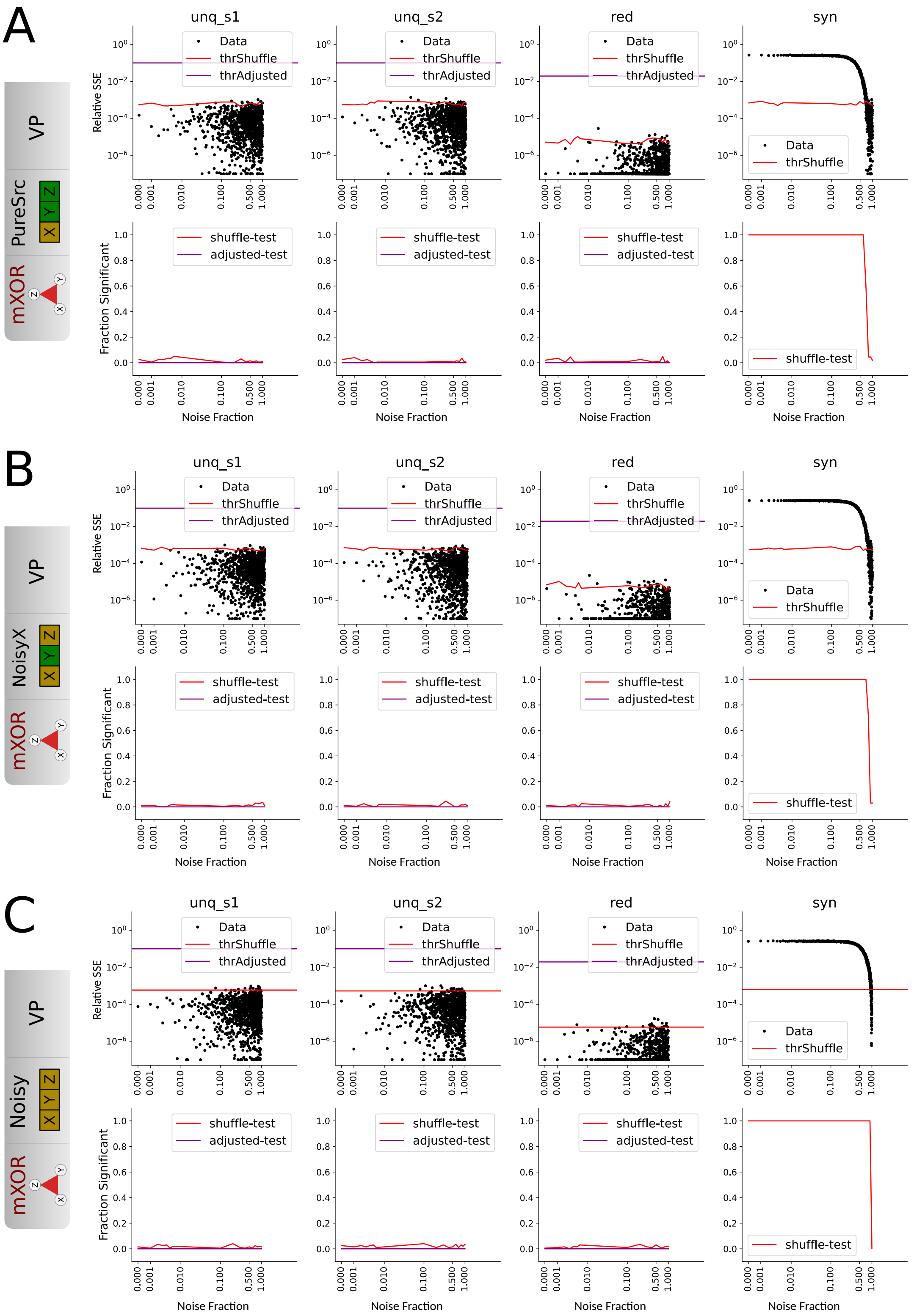

Figure 18: Variance Partitioning magnitude (top) and fraction of significant values (bottom) for **continuous** mXOR and different observable models, as function of **noise fraction** for $N_{tr} = 10000$. Red line denotes permutation testing critical value (top), and corresponding fraction of significant information atoms (bottom). Purple line denotes the same for the adjusted conservative test. Columns in each figure denote information atoms $U(X \to Z|Y)$ and $U(Y \to Z|X)$, $R(X, Y \to Z)$ and $S(X, Y \to Z)$ respectively.

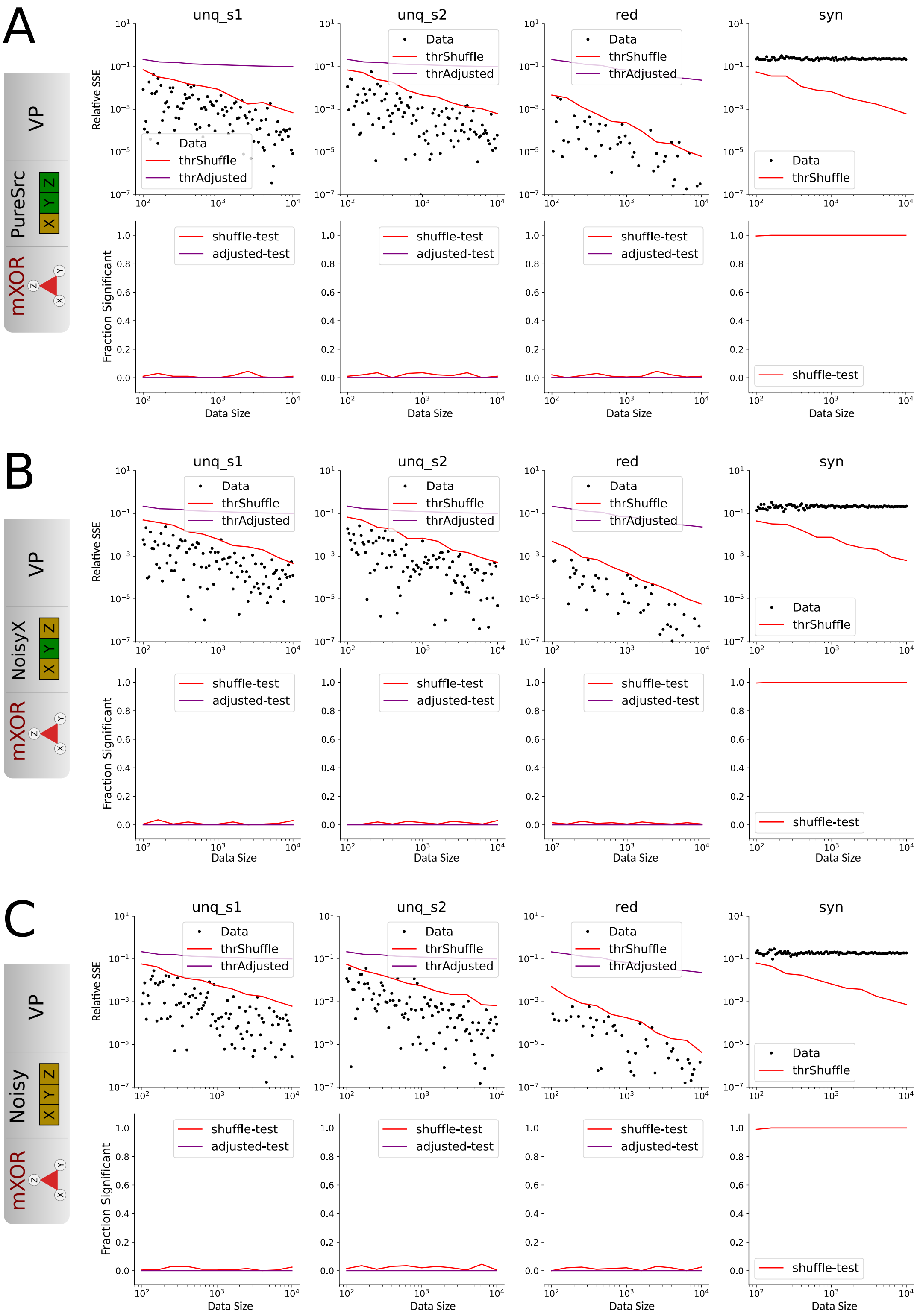

Figure 19: Variance Partitioning magnitude (top) and fraction of significant values (bottom) for **continuous** mXOR and different observable models, as function of **data size** for fixed noise fraction 0.25. Red line denotes permutation testing critical value (top), and corresponding fraction of significant information atoms (bottom). Purple line denotes the same for the adjusted conservative test. Columns in each figure denote information atoms $U(X \rightarrow Z|Y)$ and $U(Y \rightarrow Z|X)$, $R(X, Y \rightarrow Z)$ and $S(X, Y \rightarrow Z)$ respectively.

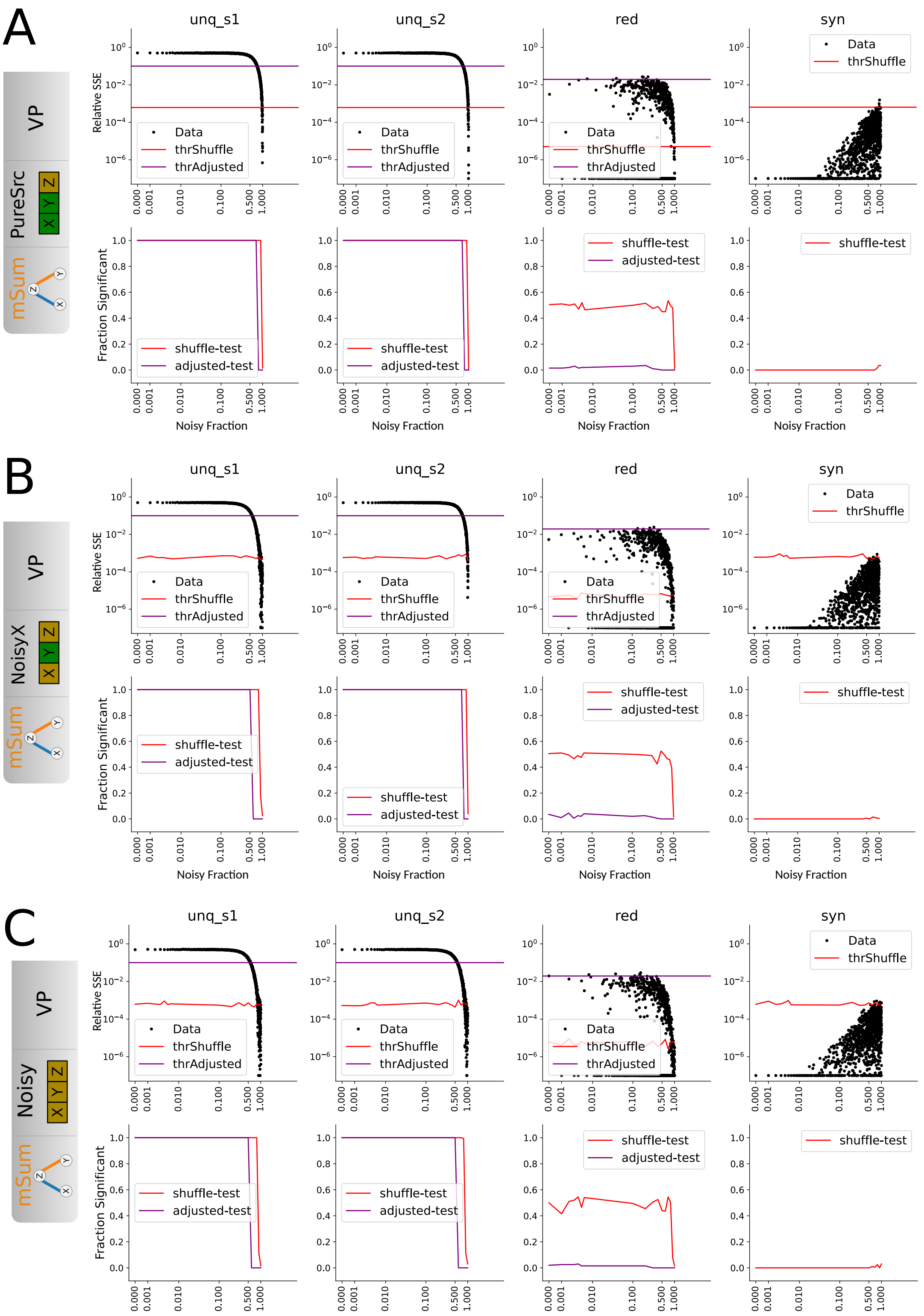

Figure 20: Variance Partitioning magnitude (top) and fraction of significant values (bottom) for **continuous** mSUM and different observable models, as function of **noise fraction** for $N_{tr} = 10000$. Red line denotes permutation testing critical value (top), and corresponding fraction of significant information atoms (bottom). Purple line denotes the same for the adjusted conservative test. Columns in each figure denote information atoms $U(X \to Z|Y)$ and $U(Y \to Z|X)$, $R(X,Y \to Z)$ and $S(X,Y \to Z)$ respectively.

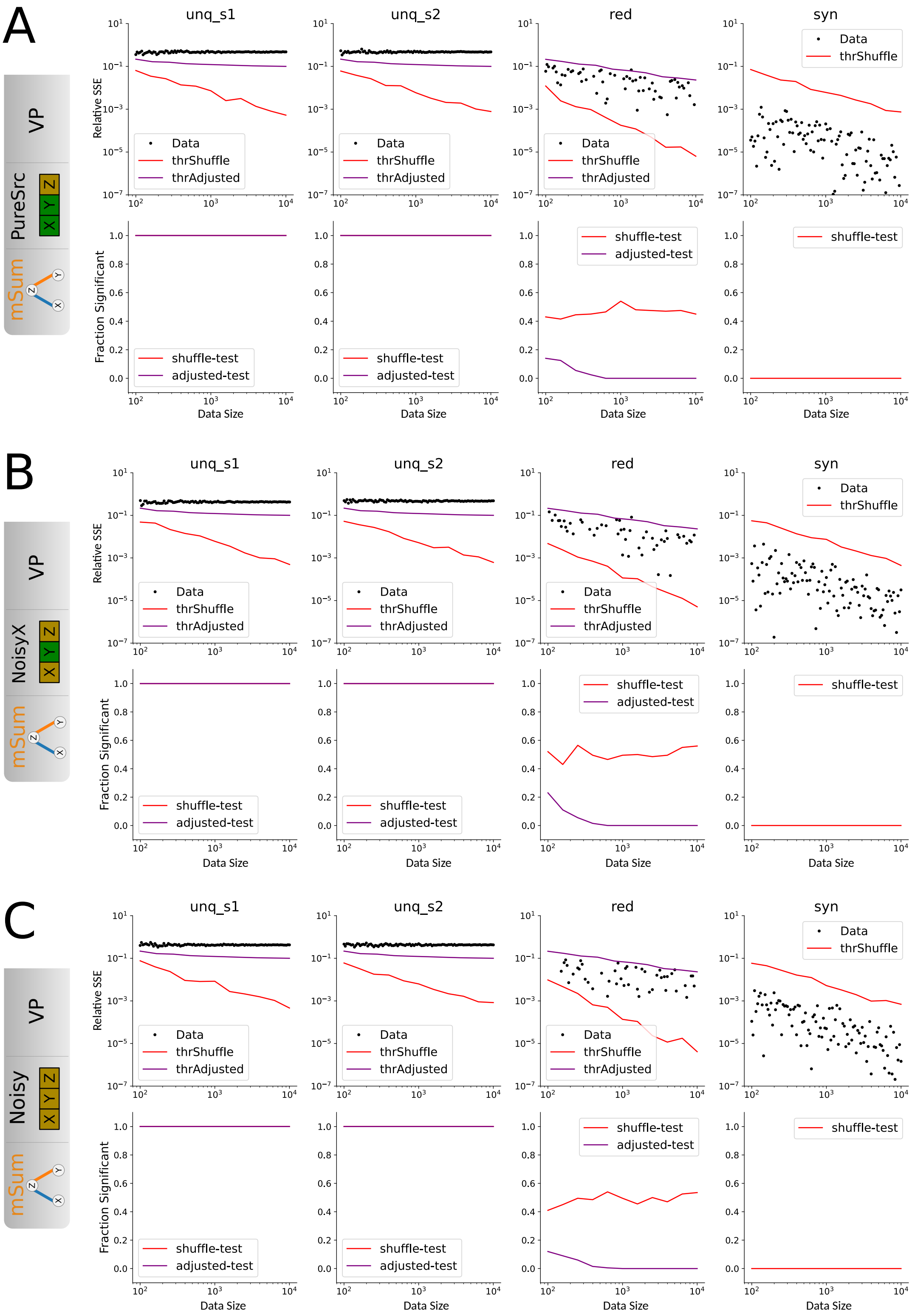

Figure 21: Variance Partitioning magnitude (top) and fraction of significant values (bottom) for **continuous** mSUM and different observable models, as function of **data size for** fixed noise fraction 0.25. Red line denotes permutation testing critical value (top), and corresponding fraction of significant information atoms (bottom). Purple line denotes the same for the adjusted conservative test. Columns in each figure denote information atoms $U(X \to Z|Y)$ and $U(Y \to Z|X)$, $R(X, Y \to Z)$ and $S(X, Y \to Z)$ respectively.

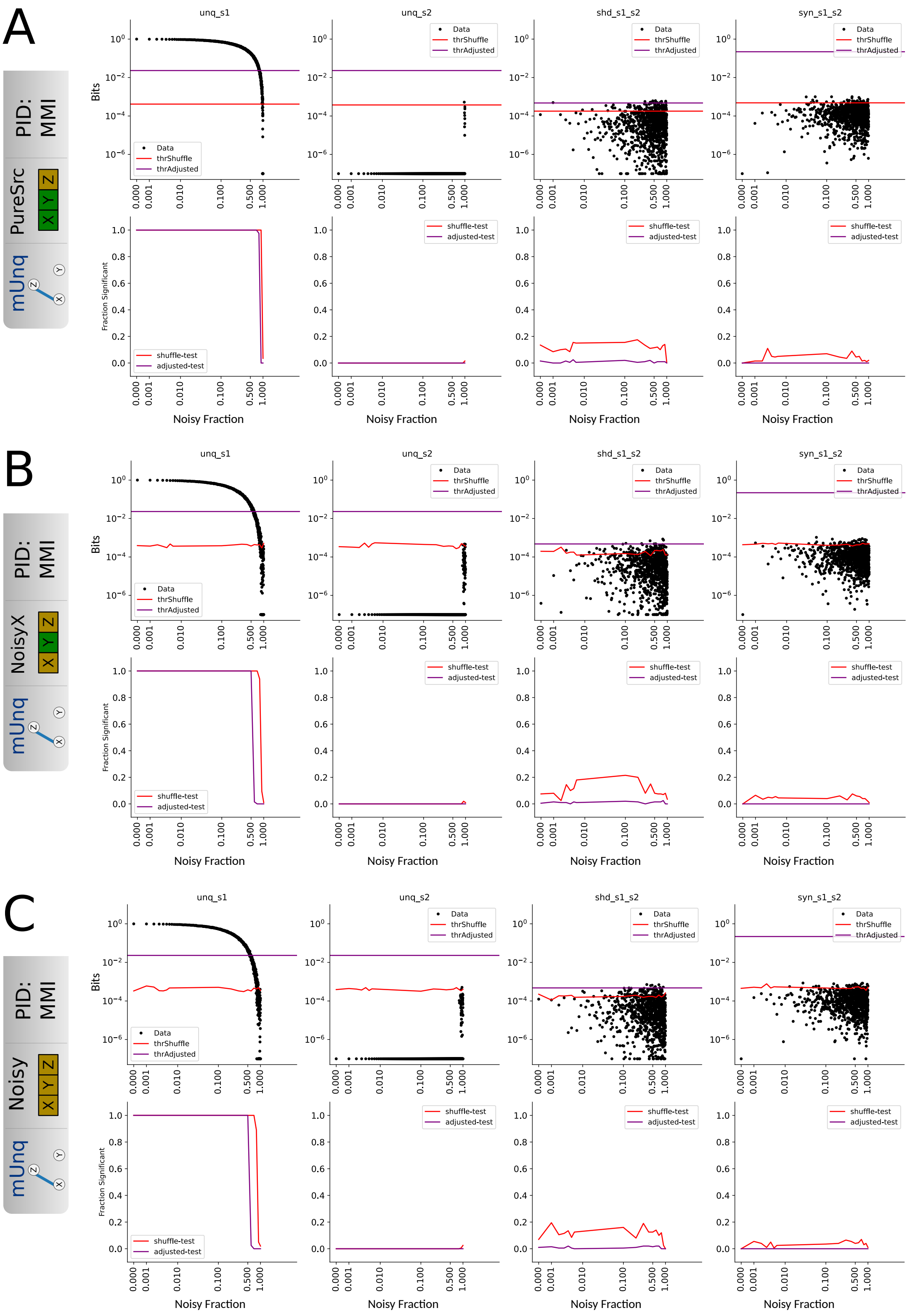

Figure 22: MMI PID magnitude (top) and fraction of significant values (bottom) for **discrete** mUnq and different observable models, as function of **noise fraction** for $N_{tr} = 10000$. Red line denotes permutation testing critical value (top), and corresponding fraction of significant information atoms (bottom). Purple line denotes the same for the adjusted conservative test. Columns in each figure denote information atoms $U(X \rightarrow Z|Y)$ and $U(Y \rightarrow Z|X)$, $R(X,Y \rightarrow Z)$ and $S(X,Y \rightarrow Z)$ respectively.

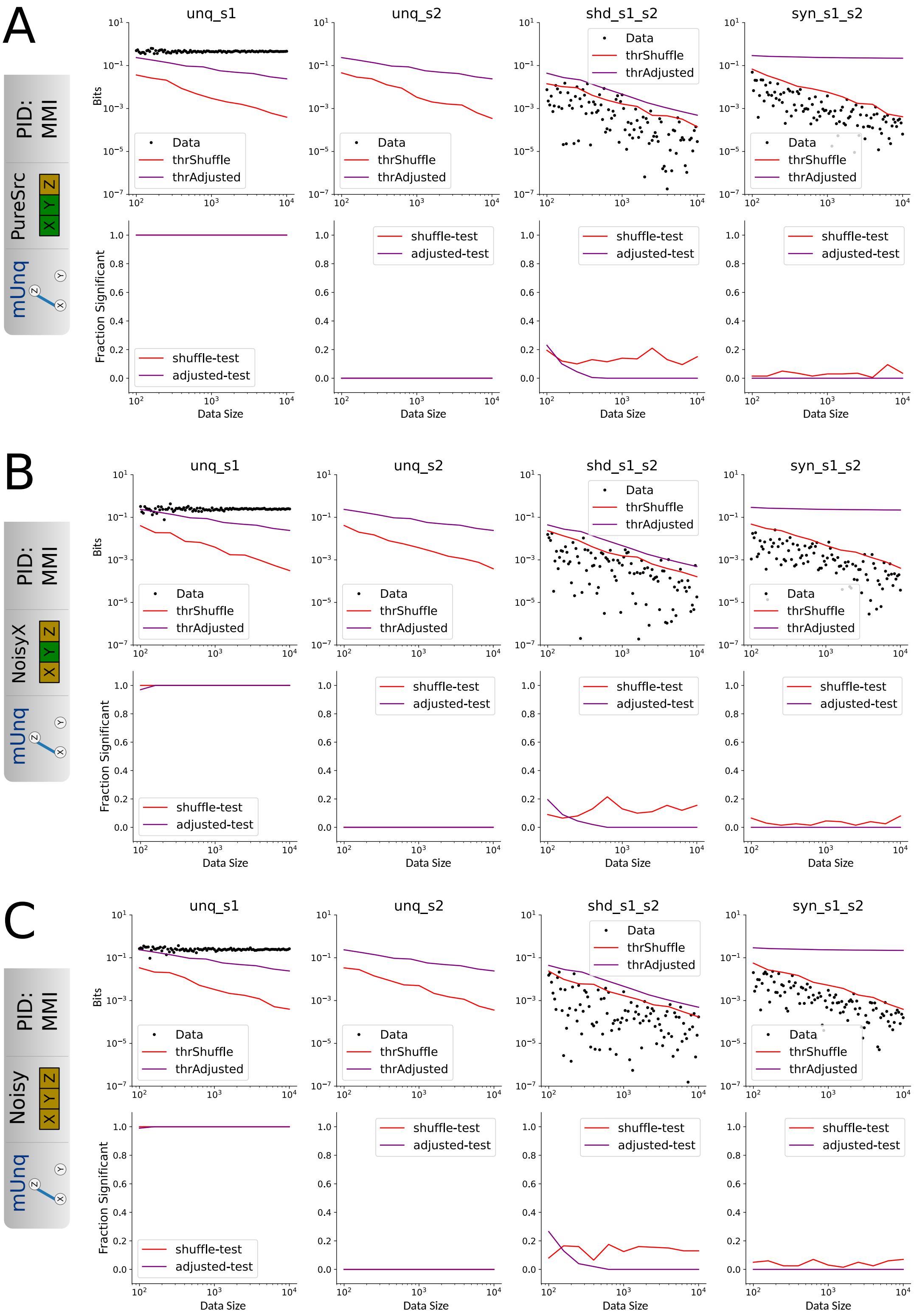

Figure 23: MMI PID magnitude (top) and fraction of significant values (bottom) for **discrete** mUnq and different observable models, as function of **data size** for fixed noise fraction 0.25. Red line denotes permutation testing critical value (top), and corresponding fraction of significant information atoms (bottom). Purple line denotes the same for the adjusted conservative test. Columns in each figure denote information atoms $U(X \to Z|Y)$ and $U(Y \to Z|X)$, $R(X, Y \to Z)$ and $S(X, Y \to Z)$ respectively.

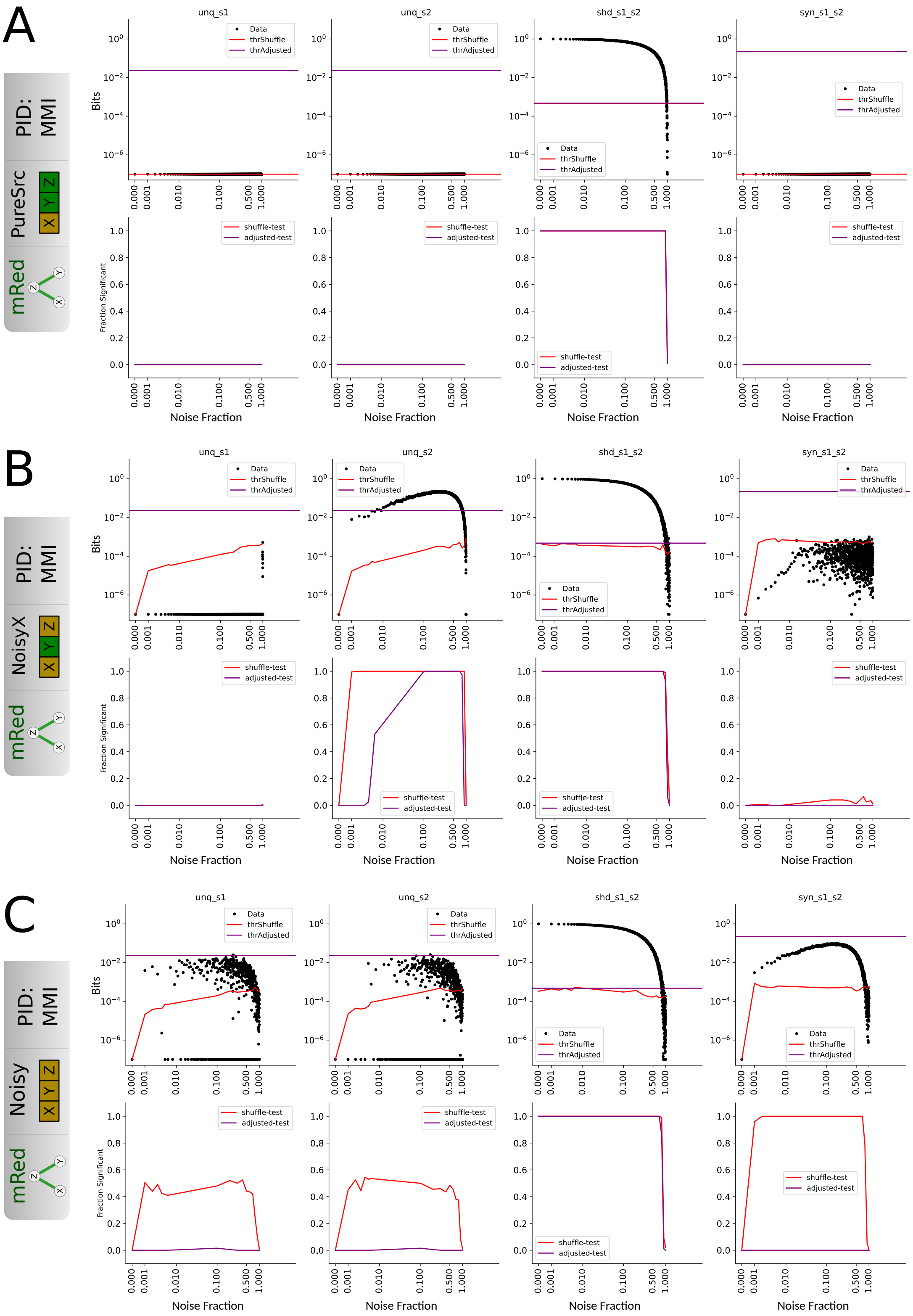

Figure 24: MMI PID magnitude (top) and fraction of significant values (bottom) for **discrete** mRed and different observable models, as function of **noise fraction** for $N_{tr} = 10000$. Red line denotes permutation testing critical value (top), and corresponding fraction of significant information atoms (bottom). Purple line denotes the same for the adjusted conservative test. Columns in each figure denote information atoms $U(X \to Z|Y)$ and $U(Y \to Z|X)$, $R(X, Y \to Z)$ and $S(X, Y \to Z)$ respectively.

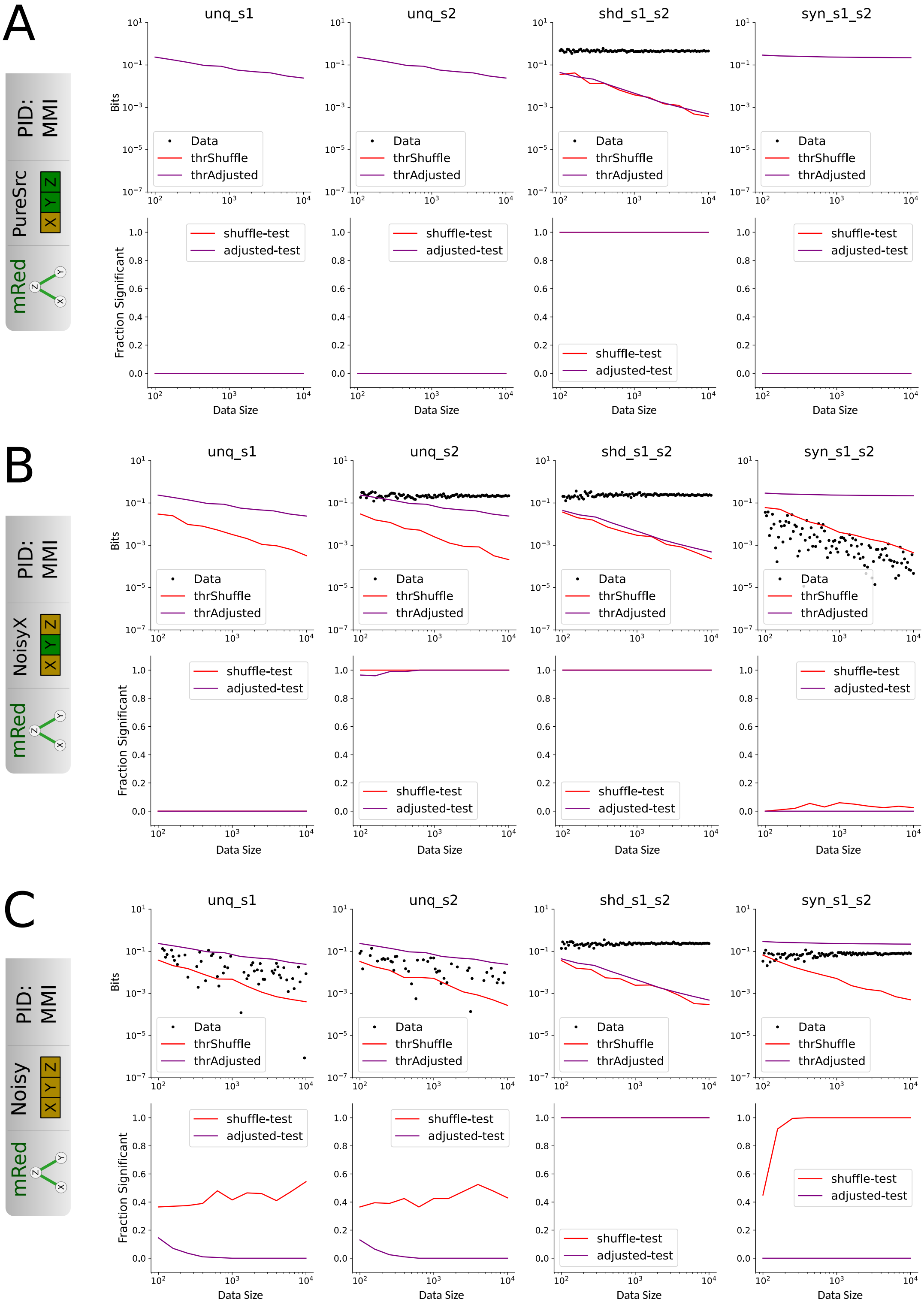

Figure 25: MMI PID magnitude (top) and fraction of significant values (bottom) for **discrete** mRed and different observable models, as function of **data size** for fixed noise fraction 0.25. Red line denotes permutation testing critical value (top), and corresponding fraction of significant information atoms (bottom). Purple line denotes the same for the adjusted conservative test. Columns in each figure denote information atoms $U(X \rightarrow Z|Y)$ and $U(Y \rightarrow Z|X)$, $R(X, Y \rightarrow Z)$ and $S(X, Y \rightarrow Z)$ respectively.

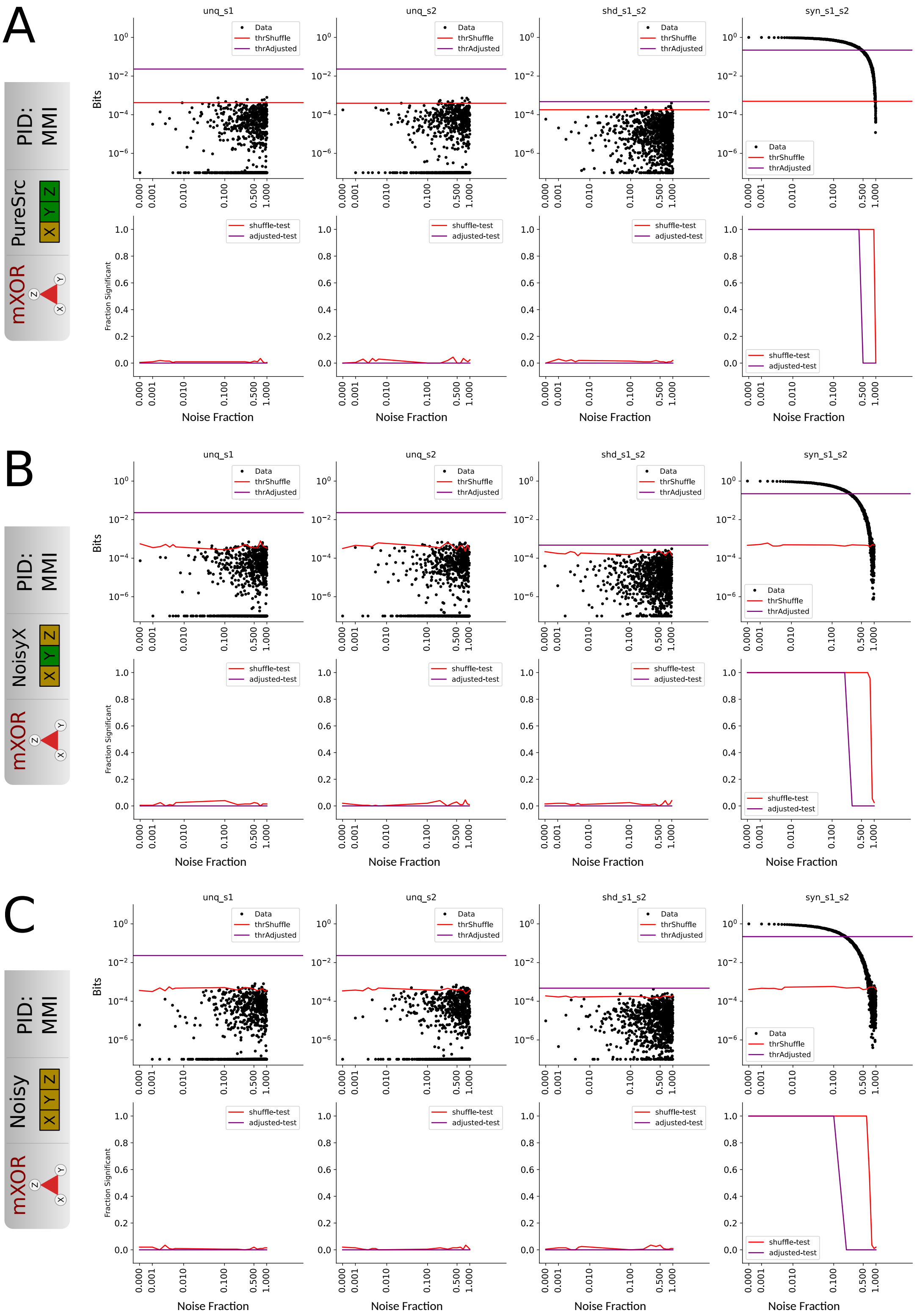

Figure 26: MMI PID magnitude (top) and fraction of significant values (bottom) for **discrete** mXOR and different observable models, as function of **noise fraction** for $N_{tr} = 10000$. Red line denotes permutation testing critical value (top), and corresponding fraction of significant information atoms (bottom). Purple line denotes the same for the adjusted conservative test. Columns in each figure denote information atoms $U(X \to Z|Y)$ and $U(Y \to Z|X)$, $R(X, Y \to Z)$ and $S(X, Y \to Z)$ respectively.

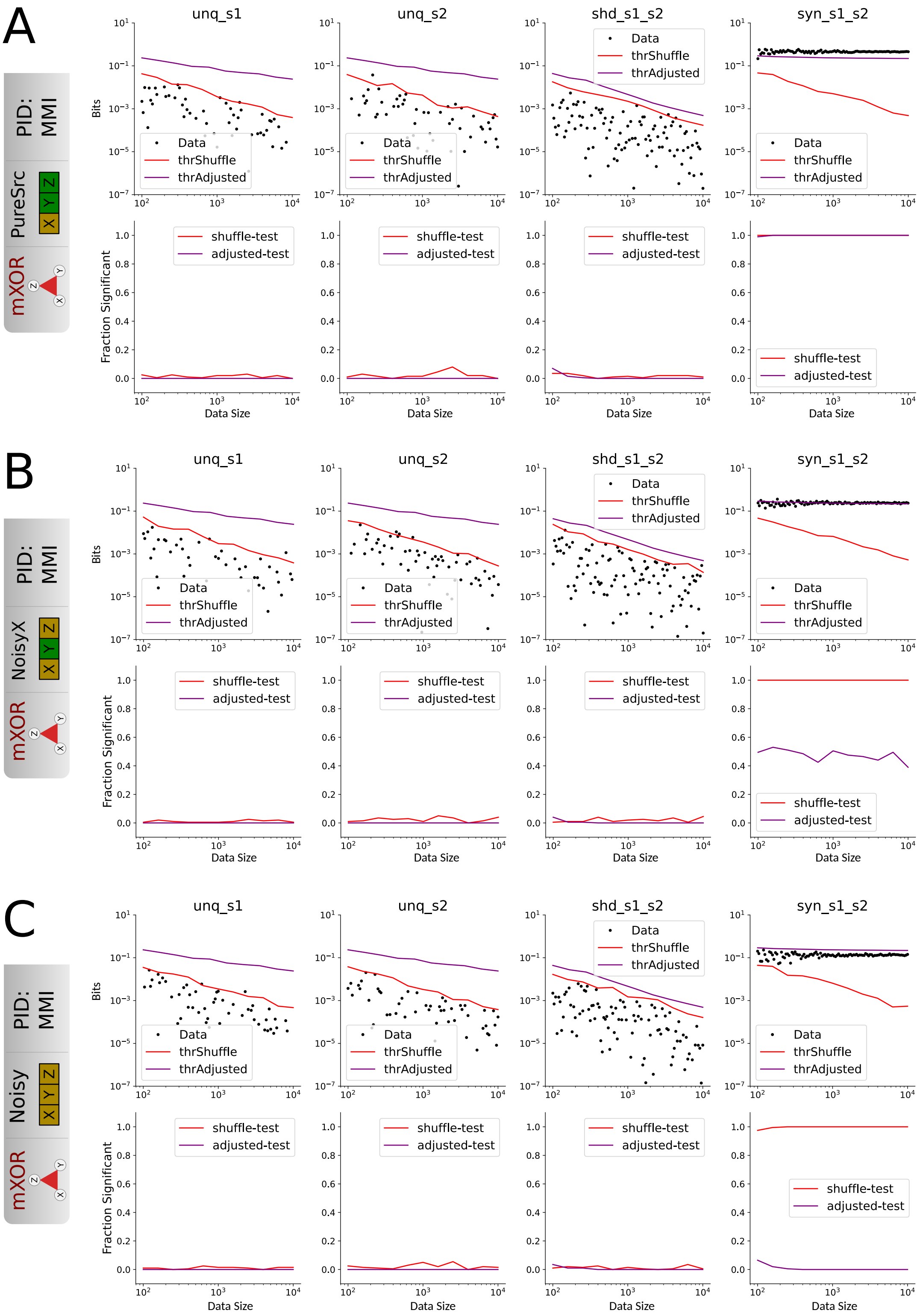

Figure 27: MMI PID magnitude (top) and fraction of significant values (bottom) for **discrete** mXOR and different observable models, as function of **data size** for fixed noise fraction 0.25. Red line denotes permutation testing critical value (top), and corresponding fraction of significant information atoms (bottom). Purple line denotes the same for the adjusted conservative test. Columns in each figure denote information atoms $U(X \to Z|Y)$ and $U(Y \to Z|X)$, $R(X, Y \to Z)$ and $S(X, Y \to Z)$ respectively.

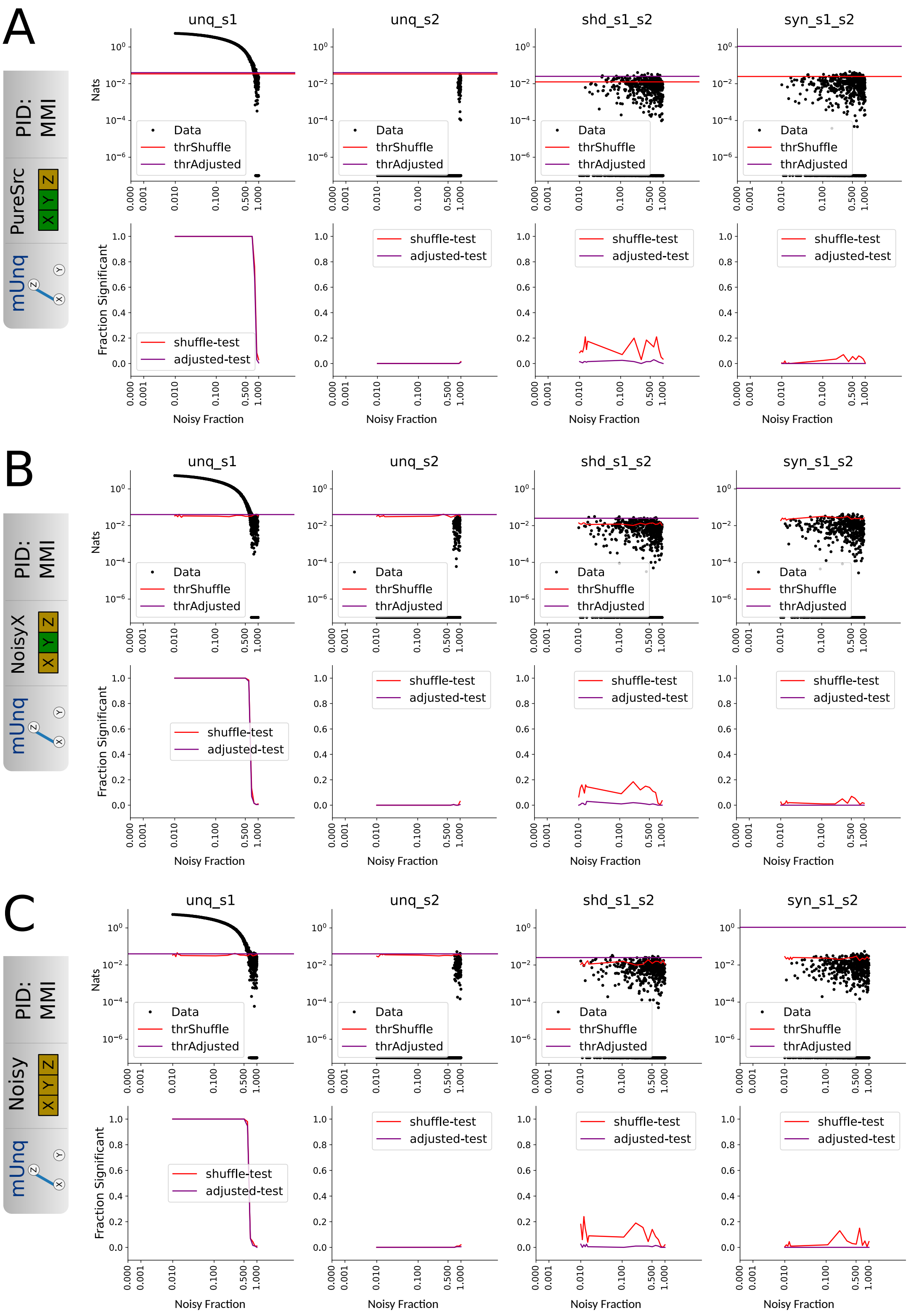

Figure 28: MMI PID magnitude (top) and fraction of significant values (bottom) for **continuous** mUnq and different observable models, as function of **noise fraction** for $N_{tr} = 10000$. Red line denotes permutation testing critical value (top), and corresponding fraction of significant information atoms (bottom). Purple line denotes the same for the adjusted conservative test. Columns in each figure denote information atoms $U(X \rightarrow Z|Y)$ and $U(Y \rightarrow Z|X)$, $R(X, Y \rightarrow Z)$ and $S(X, Y \rightarrow Z)$ respectively.

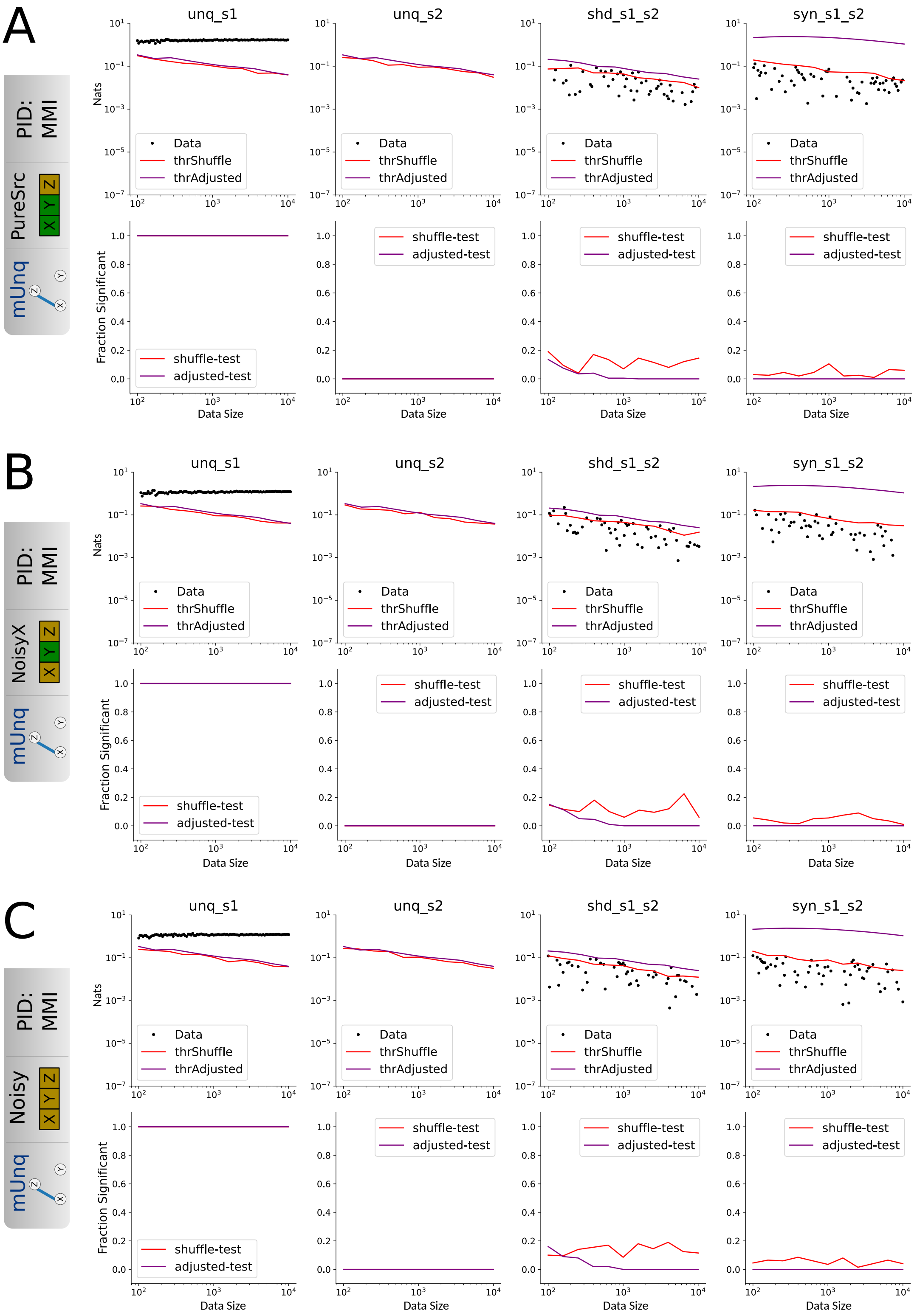

Figure 29: MMI PID magnitude (top) and fraction of significant values (bottom) for **continuous** mUnq and different observable models, as function of **data size** for fixed noise fraction 0.25. Red line denotes permutation testing critical value (top), and corresponding fraction of significant information atoms (bottom). Purple line denotes the same for the adjusted conservative test. Columns in each figure denote information atoms $U(X \to Z|Y)$ and $U(Y \to Z|X)$, $R(X, Y \to Z)$ and $S(X, Y \to Z)$ respectively.

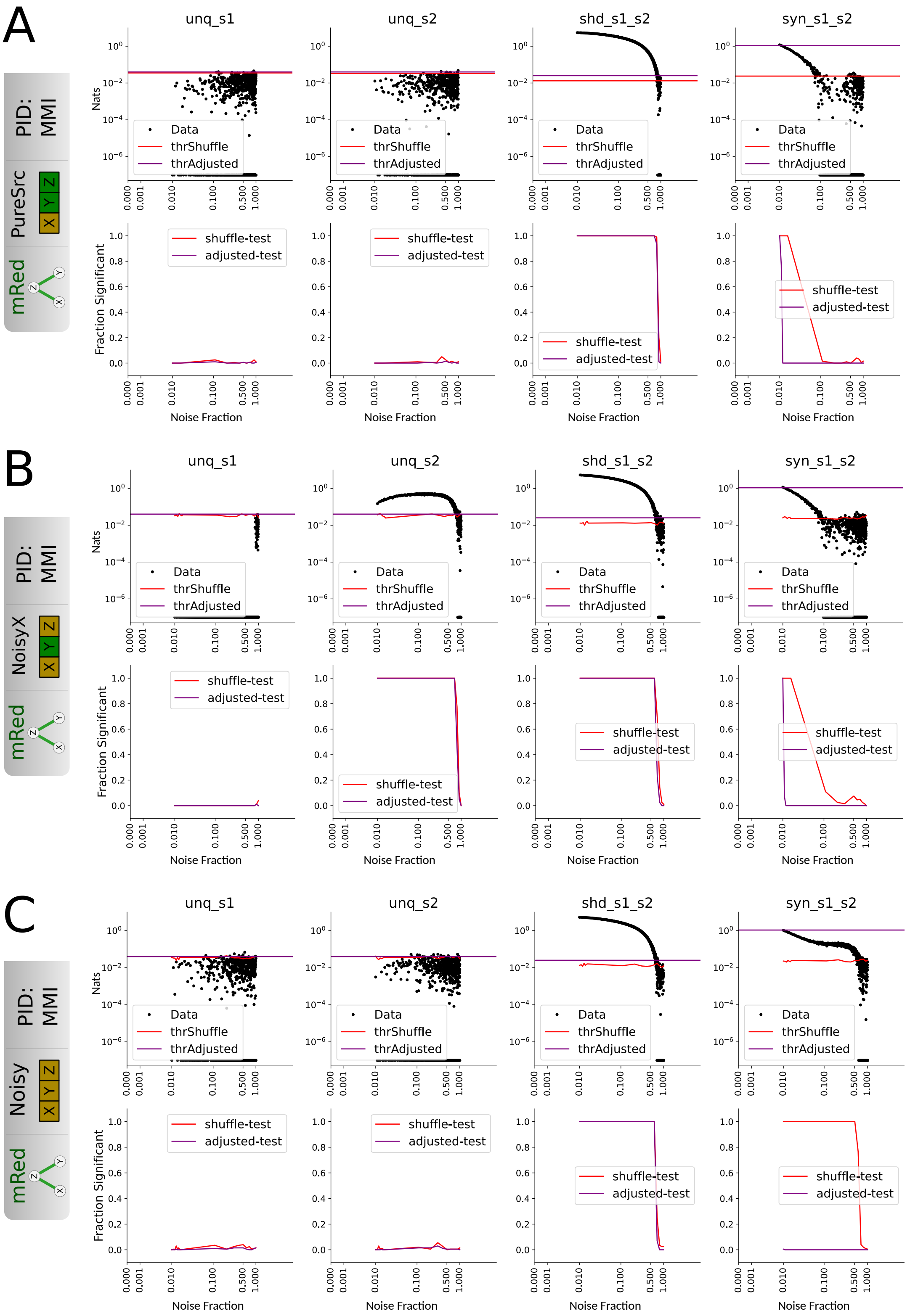

Figure 30: MMI PID magnitude (top) and fraction of significant values (bottom) for **continuous** mRed and different observable models, as function of **noise fraction** for $N_{tr} = 10000$. Red line denotes permutation testing critical value (top), and corresponding fraction of significant information atoms (bottom). Purple line denotes the same for the adjusted conservative test. Columns in each figure denote information atoms $U(X \to Z|Y)$ and $U(Y \to Z|X)$, $R(X, Y \to Z)$ and $S(X, Y \to Z)$ respectively.

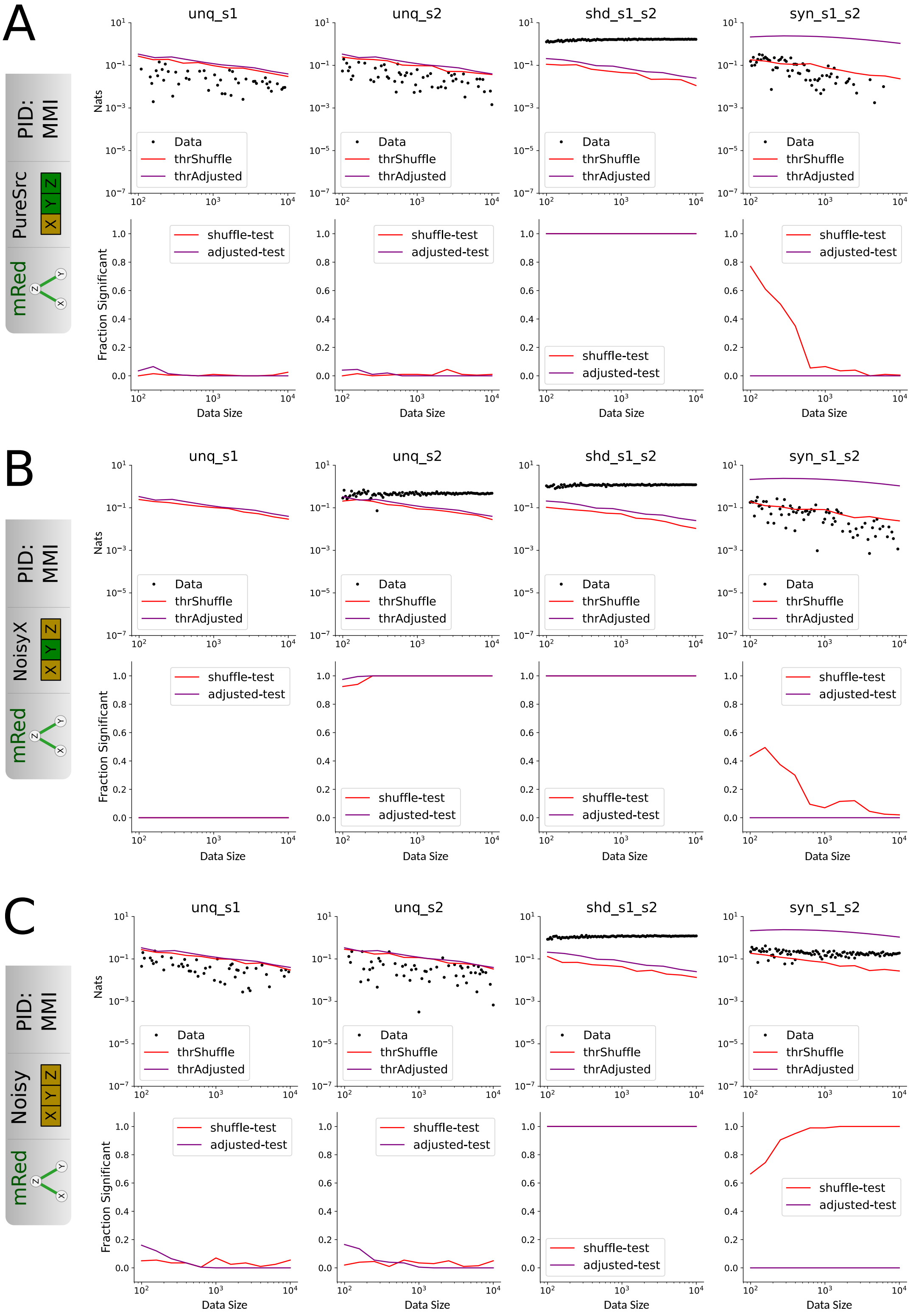

Figure 31: MMI PID magnitude (top) and fraction of significant values (bottom) for **continuous** mRed and different observable models, as function of **data size** for fixed noise fraction 0.25. Red line denotes permutation testing critical value (top), and corresponding fraction of significant information atoms (bottom). Purple line denotes the same for the adjusted conservative test. Columns in each figure denote information atoms $U(X \to Z|Y)$ and $U(Y \to Z|X)$, $R(X,Y \to Z)$ and $S(X,Y \to Z)$ respectively.

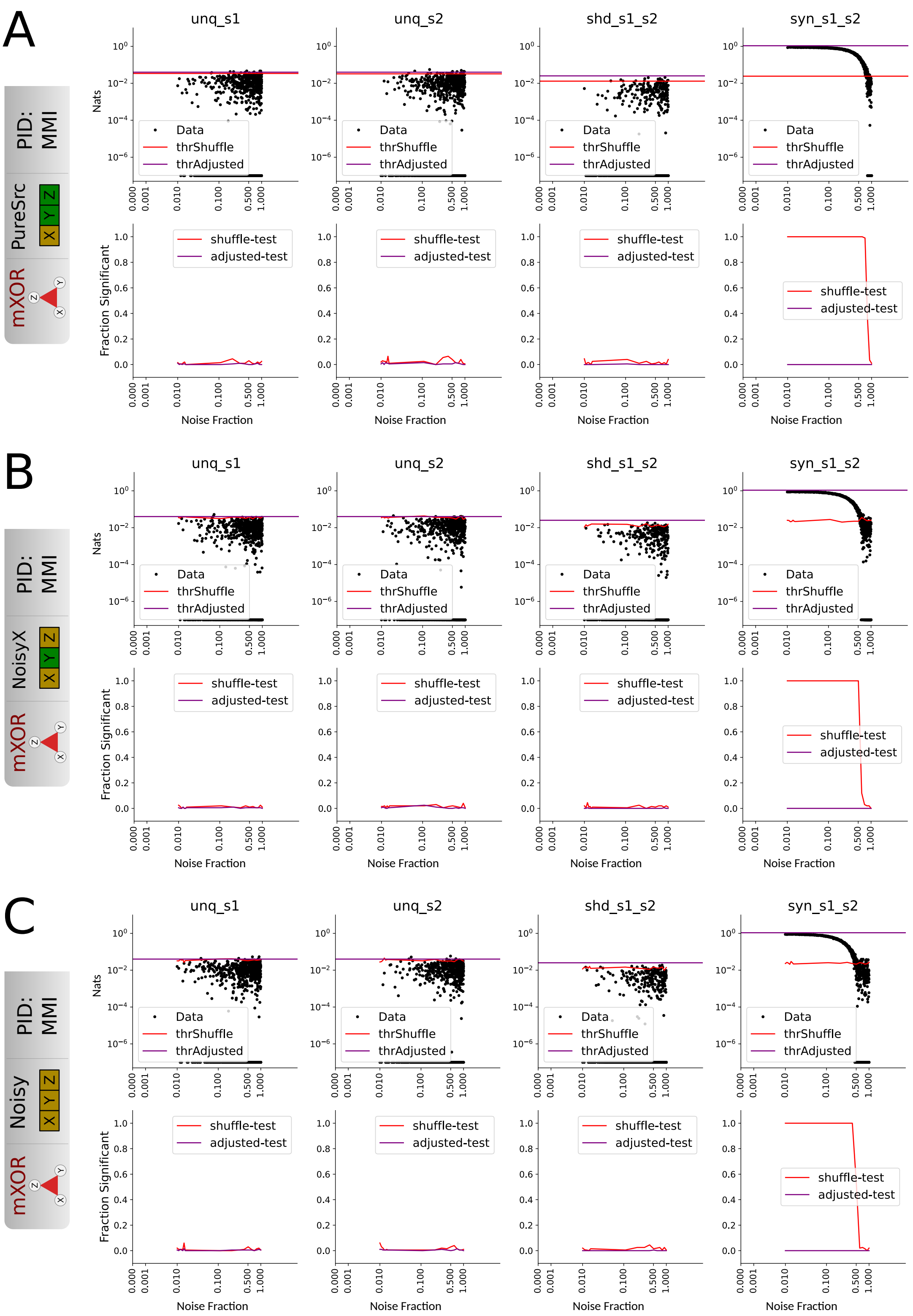

Figure 32: MMI PID magnitude (top) and fraction of significant values (bottom) for **continuous** mXOR and different observable models, as function of **noise fraction** for $N_{tr} = 10000$. Red line denotes permutation testing critical value (top), and corresponding fraction of significant information atoms (bottom). Purple line denotes the same for the adjusted conservative test. Columns in each figure denote information atoms $U(X \to Z|Y)$ and $U(Y \to Z|X)$, $R(X, Y \to Z)$ and $S(X, Y \to Z)$ respectively.

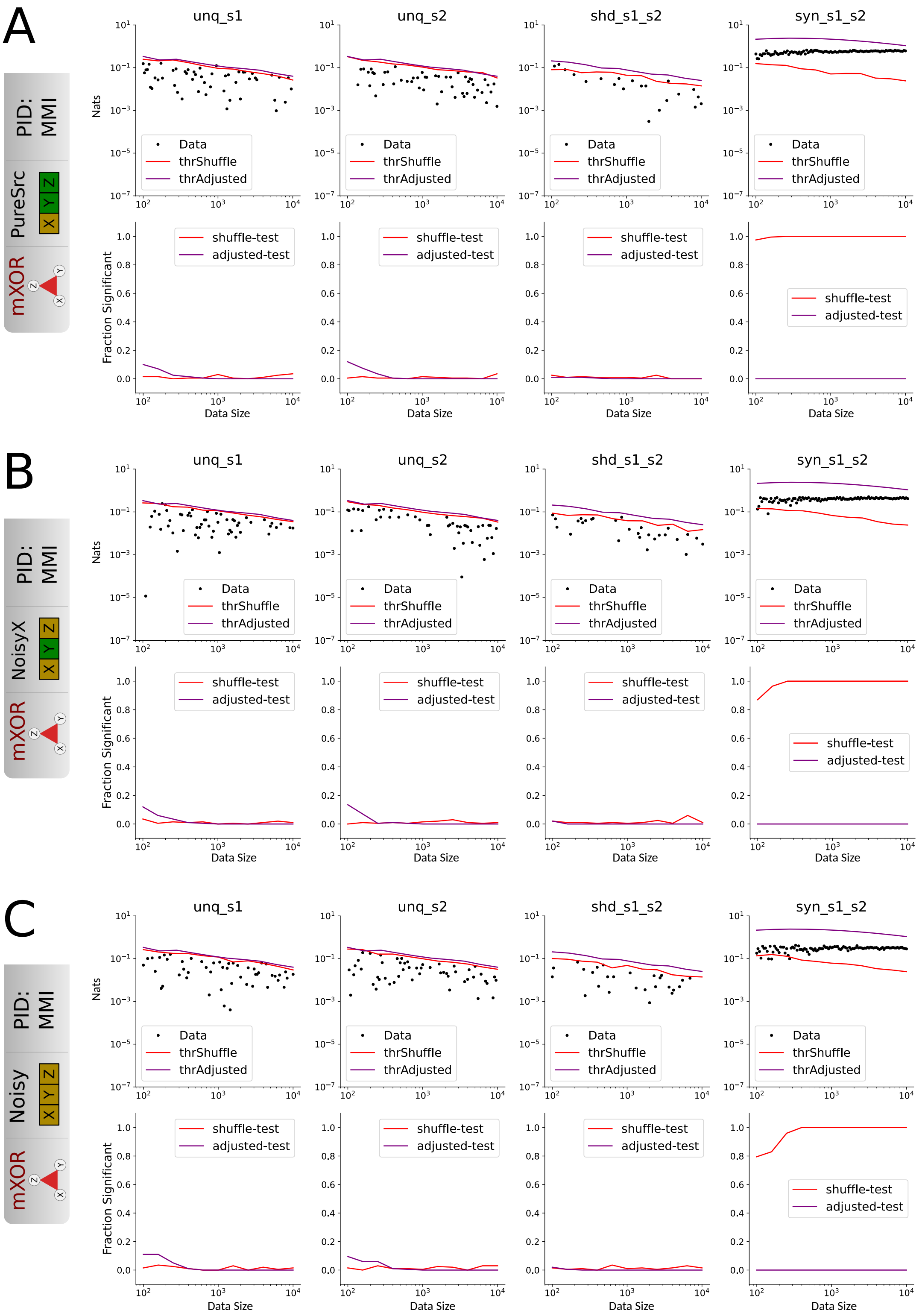

Figure 33: MMI PID magnitude (top) and fraction of significant values (bottom) for **continuous** mXOR and different observable models, as function of **data size** for fixed noise fraction 0.25. Red line denotes permutation testing critical value (top), and corresponding fraction of significant information atoms (bottom). Purple line denotes the same for the adjusted conservative test. Columns in each figure denote information atoms $U(X \to Z|Y)$ and $U(Y \to Z|X)$, $R(X, Y \to Z)$ and $S(X, Y \to Z)$ respectively.

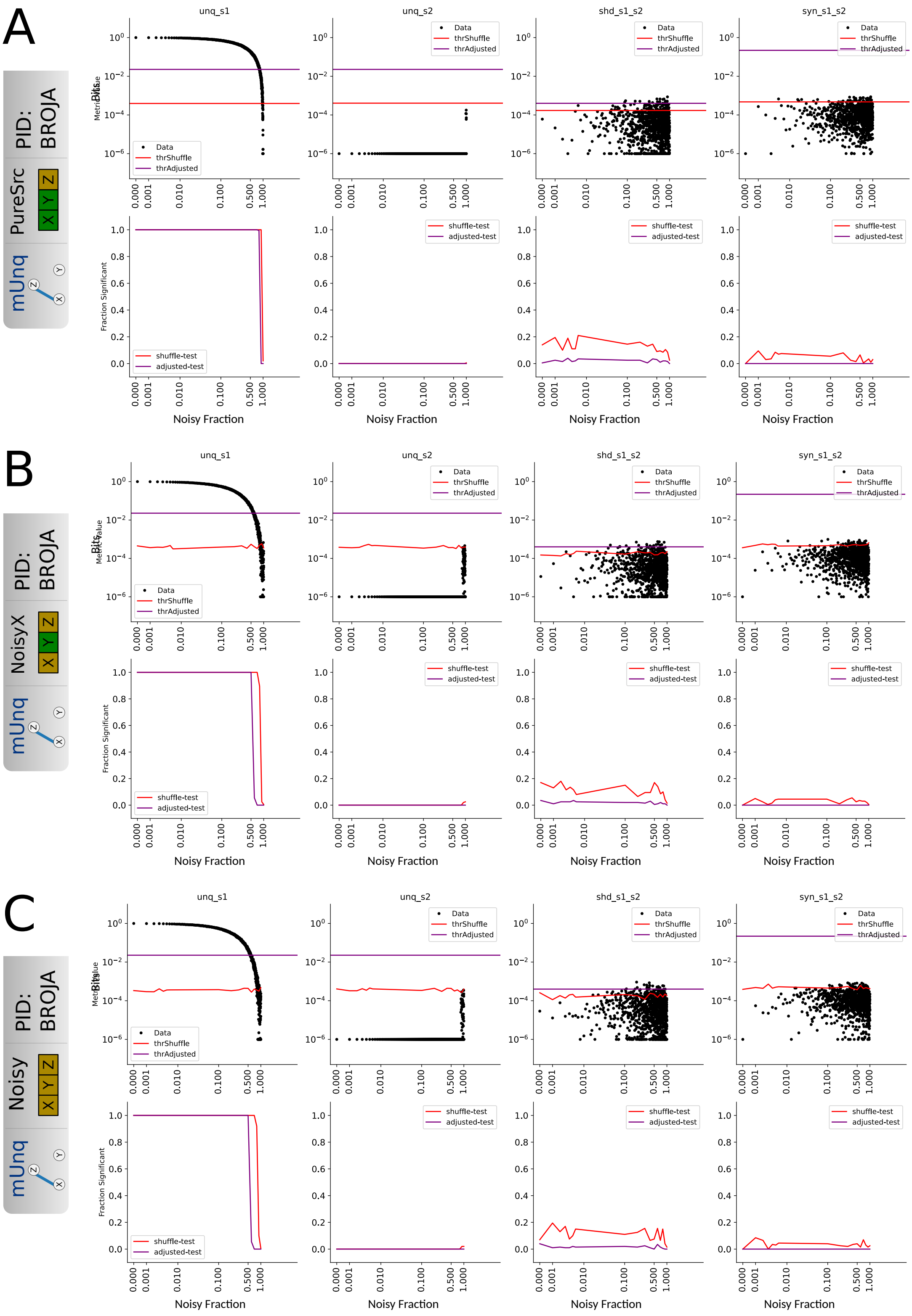

Figure 34: BROJA PID magnitude (top) and fraction of significant values (bottom) for **discrete** mUnq and different observable models, as function of **noise fraction** for $N_{tr} = 10000$. Red line denotes permutation testing critical value (top), and corresponding fraction of significant information atoms (bottom). Purple line denotes the same for the adjusted conservative test. Columns in each figure denote information atoms $U(X \rightarrow Z|Y)$ and $U(Y \rightarrow Z|X)$, $R(X,Y \rightarrow Z)$ and $S(X,Y \rightarrow Z)$ respectively.

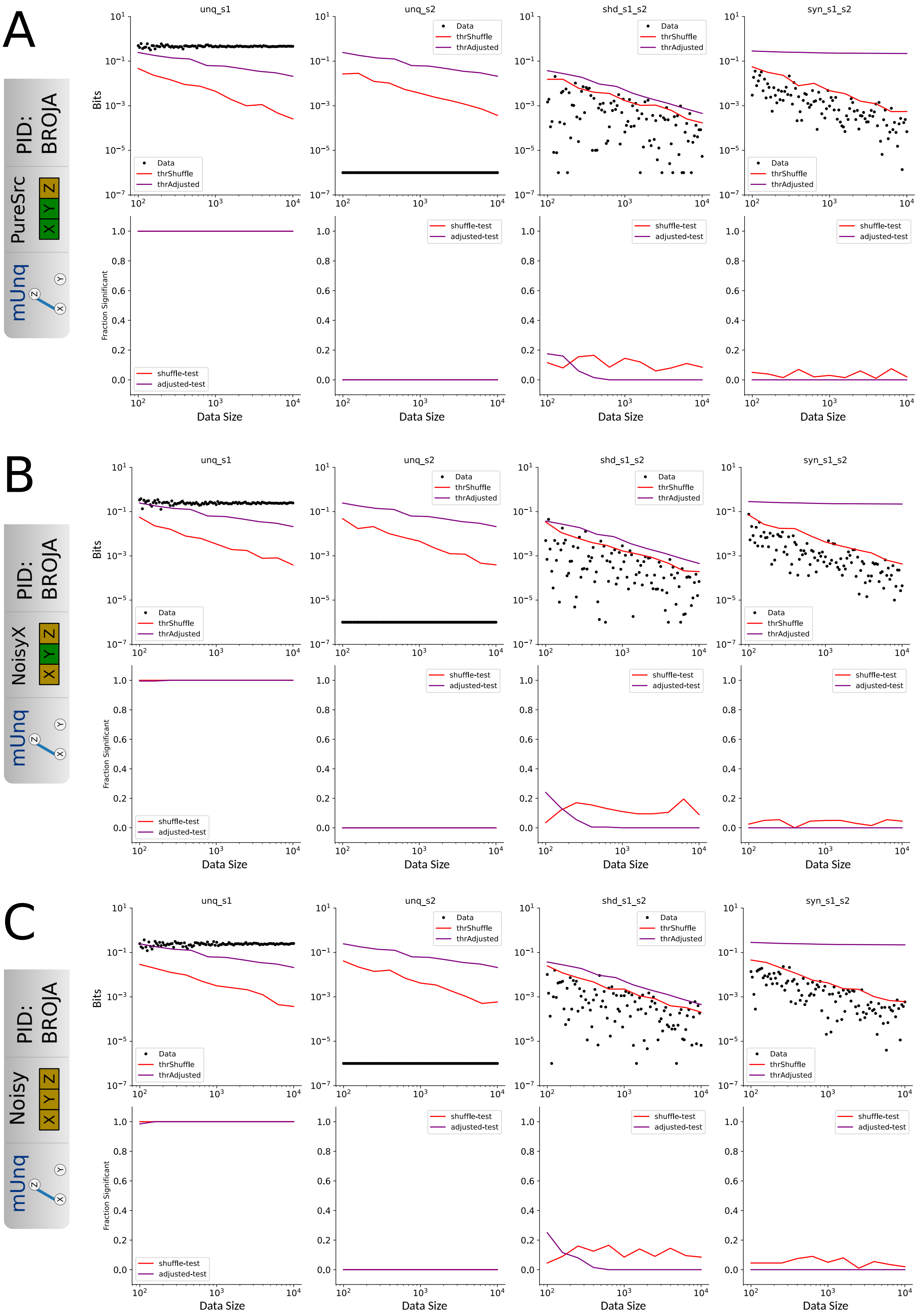

Figure 35: BROJA PID magnitude (top) and fraction of significant values (bottom) for **discrete** mUnq and different observable models, as function of **data size** for fixed noise fraction 0.25. Red line denotes permutation testing critical value (top), and corresponding fraction of significant information atoms (bottom). Purple line denotes the same for the adjusted conservative test. Columns in each figure denote information atoms $U(X \to Z|Y)$ and $U(Y \to Z|X)$, $R(X,Y \to Z)$ and $S(X,Y \to Z)$ respectively.

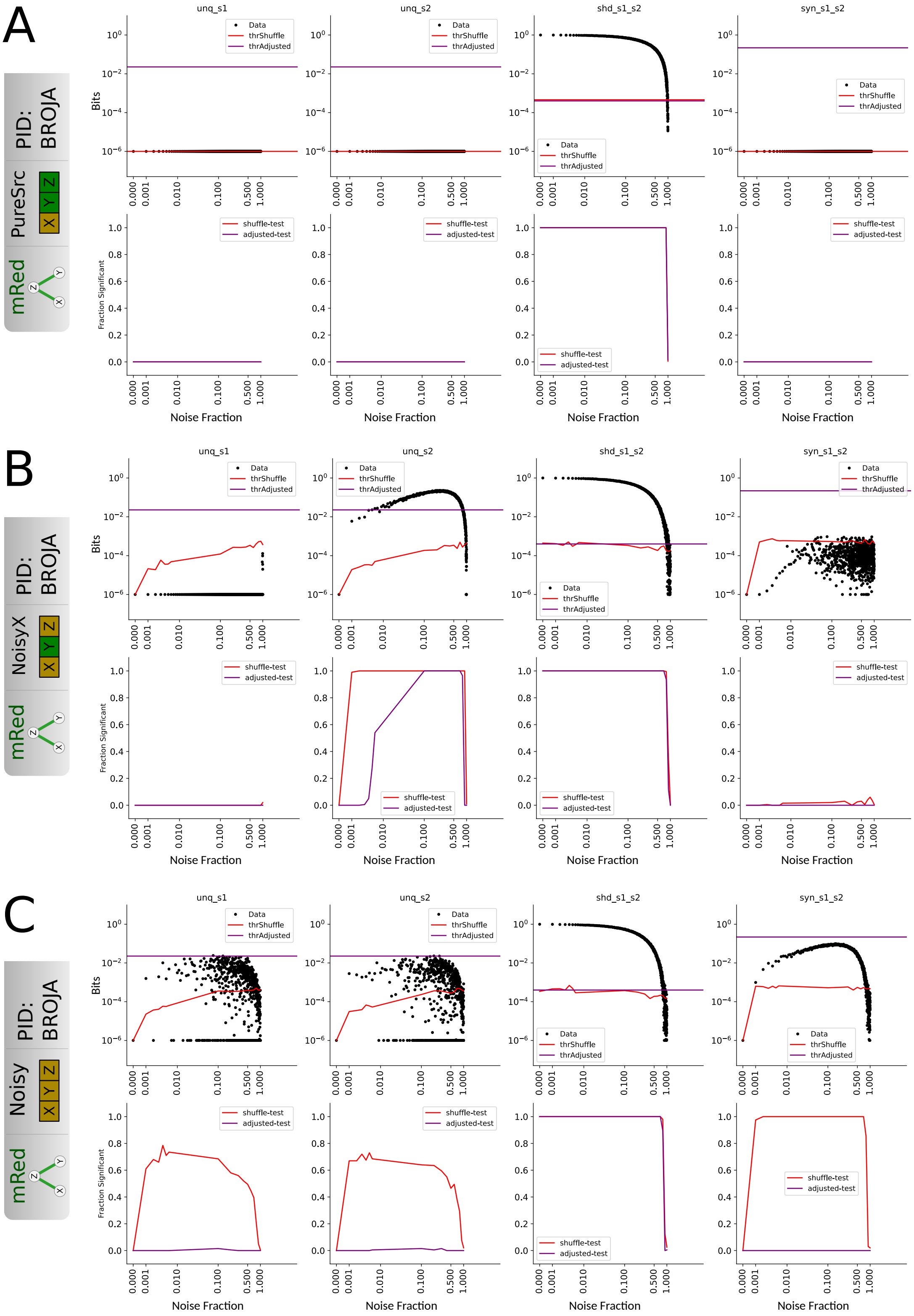

Figure 36: BROJA PID magnitude (top) and fraction of significant values (bottom) for **discrete** mRed and different observable models, as function of **noise fraction** for $N_{tr} = 10000$. Red line denotes permutation testing critical value (top), and corresponding fraction of significant information atoms (bottom). Purple line denotes the same for the adjusted conservative test. Columns in each figure denote information atoms $U(X \rightarrow Z|Y)$ and $U(Y \rightarrow Z|X)$, $R(X, Y \rightarrow Z)$ and $S(X, Y \rightarrow Z)$ respectively.

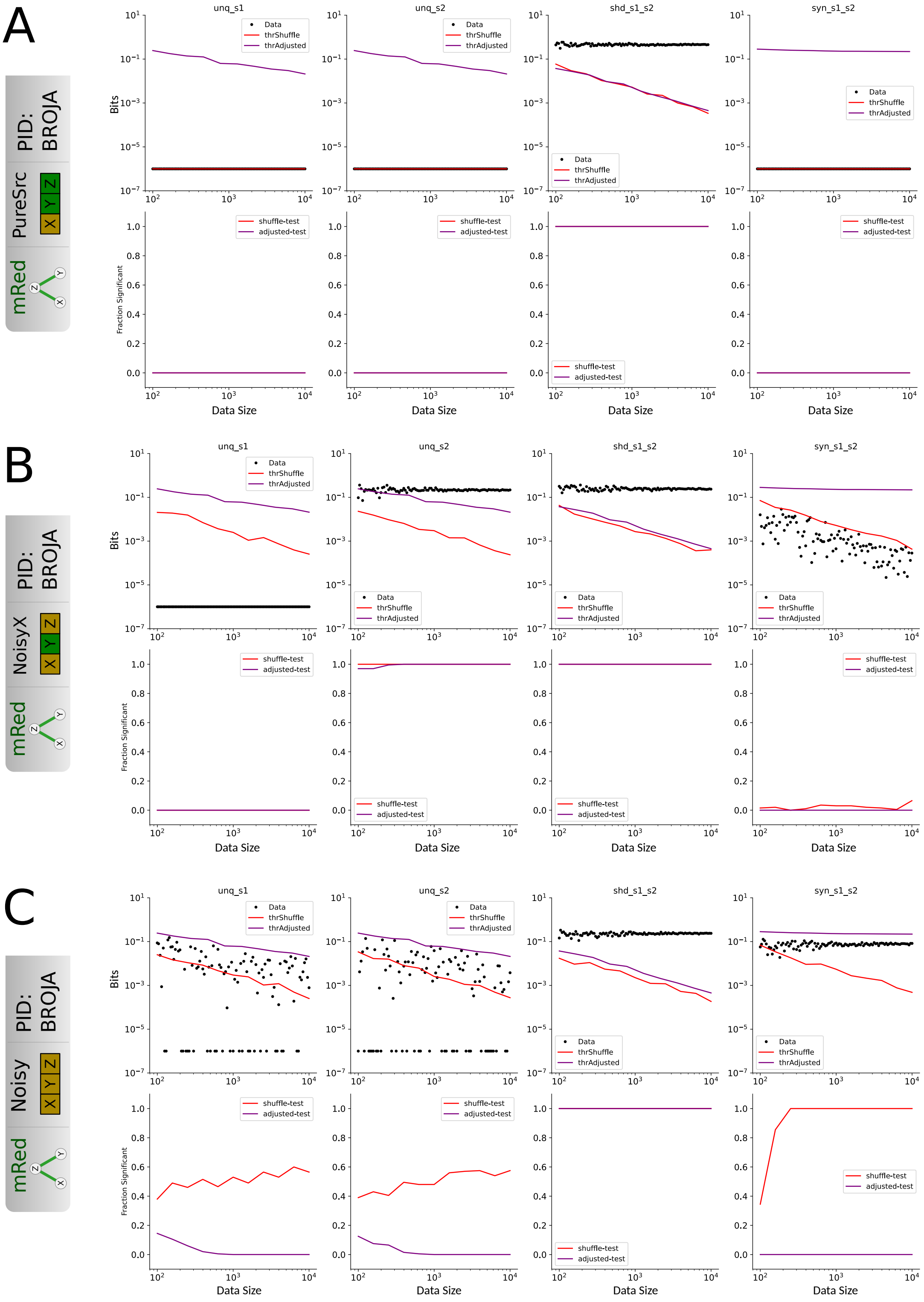

Figure 37: BROJA PID magnitude (top) and fraction of significant values (bottom) for **discrete** mRed and different observable models, as function of **data size** for fixed noise fraction 0.25. Red line denotes permutation testing critical value (top), and corresponding fraction of significant information atoms (bottom). Purple line denotes the same for the adjusted conservative test. Columns in each figure denote information atoms $U(X \to Z|Y)$ and $U(Y \to Z|X)$, $R(X,Y \to Z)$ and $S(X,Y \to Z)$ respectively.

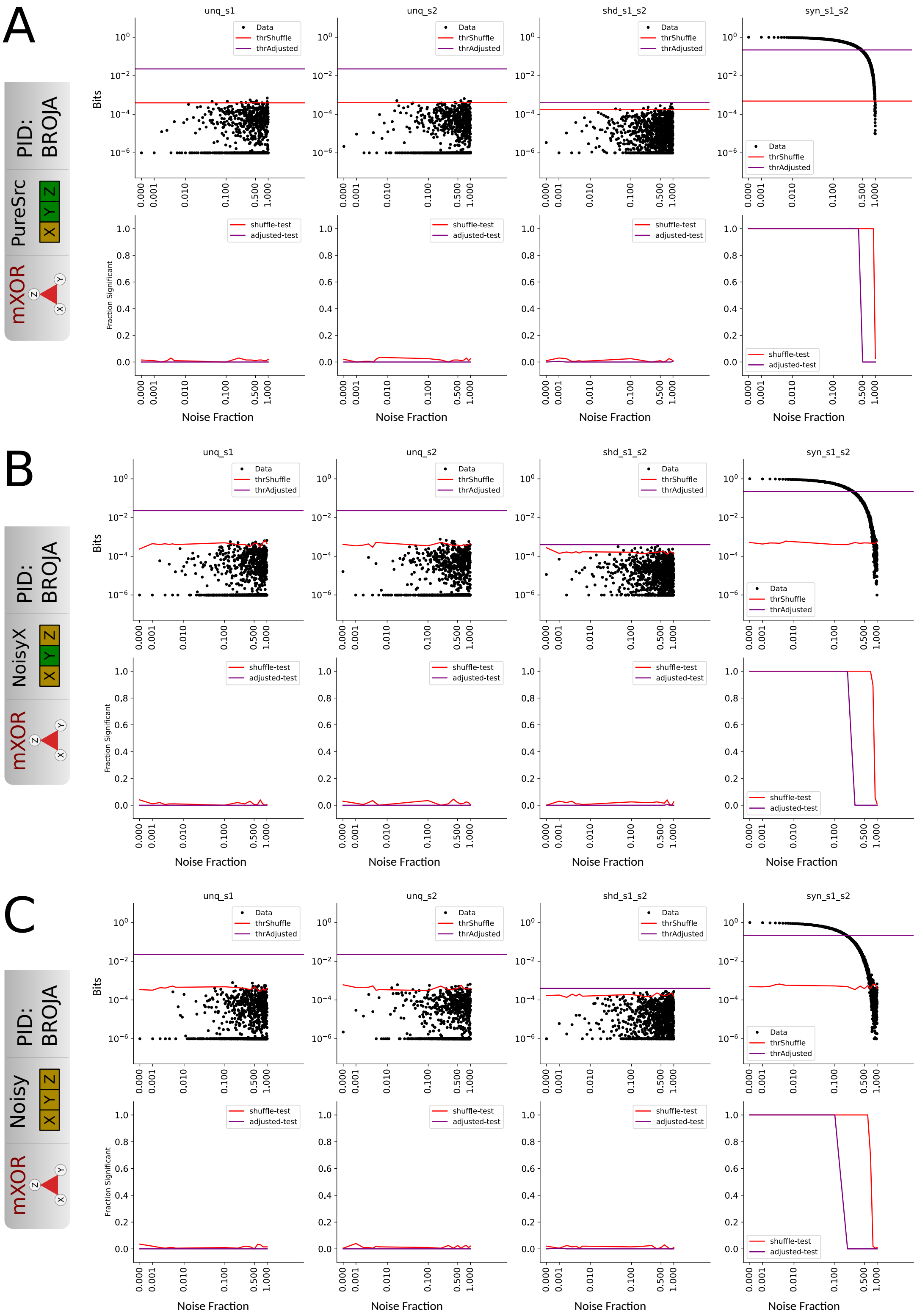

Figure 38: BROJA PID magnitude (top) and fraction of significant values (bottom) for **discrete** mXOR and different observable models, as function of **noise fraction** for $N_{tr} = 10000$. Red line denotes permutation testing critical value (top), and corresponding fraction of significant information atoms (bottom). Purple line denotes the same for the adjusted conservative test. Columns in each figure denote information atoms $U(X \to Z|Y)$ and $U(Y \to Z|X)$, $R(X, Y \to Z)$ and $S(X, Y \to Z)$ respectively.

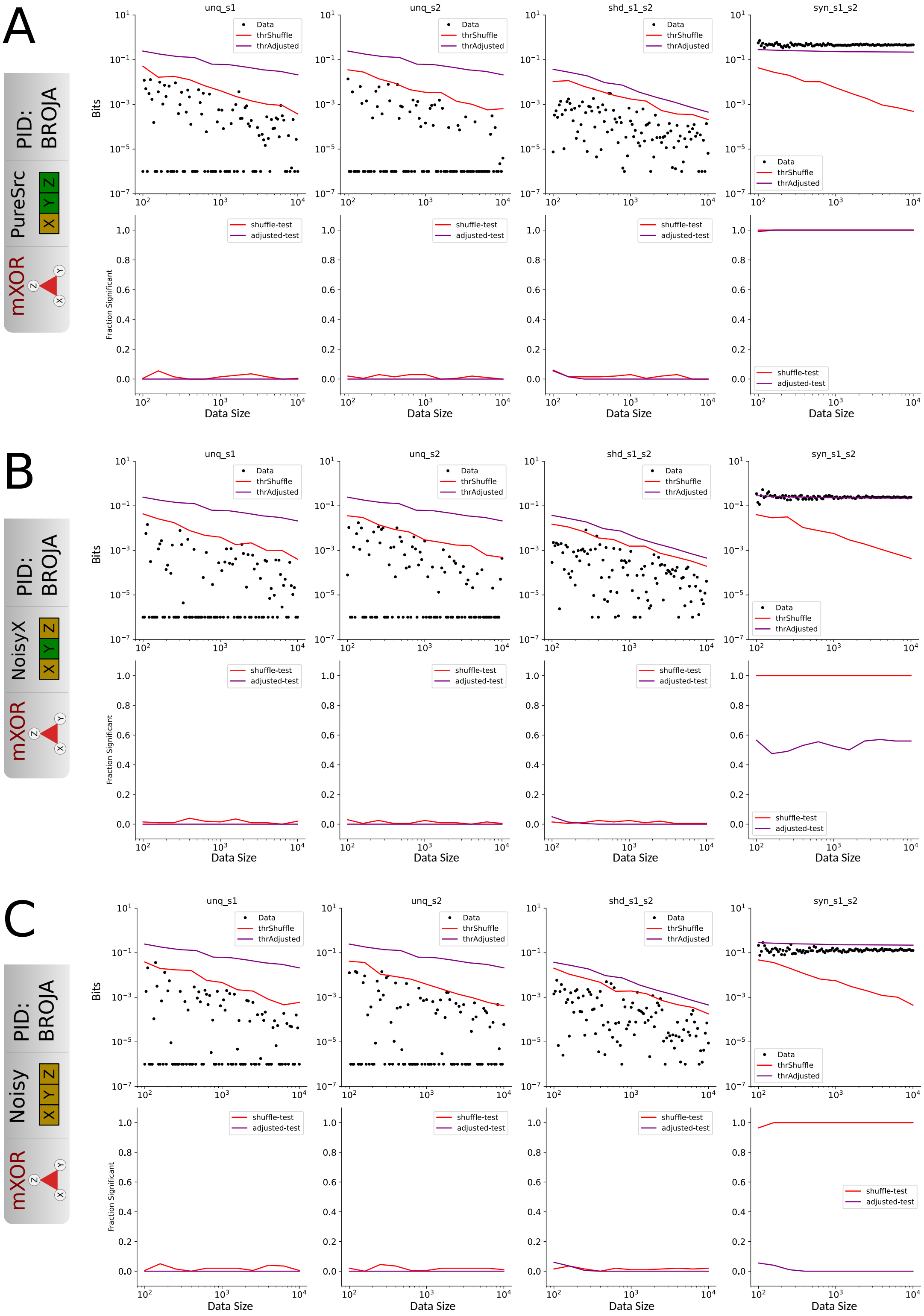

Figure 39: BROJA PID magnitude (top) and fraction of significant values (bottom) for **discrete** mXOR and different observable models, as function of **data size** for fixed noise fraction 0.25. Red line denotes permutation testing critical value (top), and corresponding fraction of significant information atoms (bottom). Purple line denotes the same for the adjusted conservative test. Columns in each figure denote information atoms $U(X \to Z|Y)$ and $U(Y \to Z|X)$, $R(X, Y \to Z)$ and $S(X, Y \to Z)$ respectively.

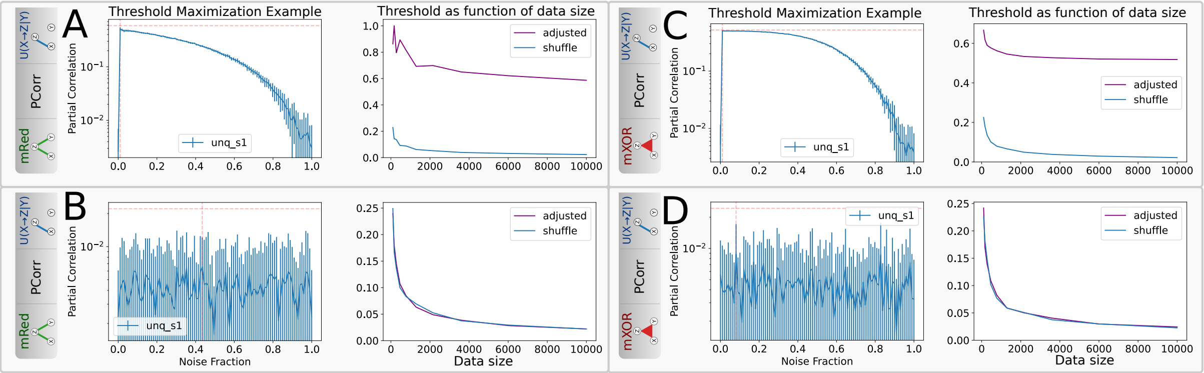

Figure 40: Adjusted maximization of critical value for Partial Correlation for discrete models (A, B) and continuous models (C,D) using redundant adversarial model (A,C) and synergistic adversarial model (B,D). Left) False positive unique information atom distributions are plotted as function of noise fraction for data size $N_{tr} = 10000$. Vertical dashed line denotes the noise fraction corresponding to the highest critical value. Horizontal dashed line indicates the critical value (upper 1% quantile) at that noise fraction. Right) Estimated critical values are plotted as function of data size. Blue denotes the critical value due to permutation-testing, purple denotes the adjusted critical value. For the redundant model the adjusted critical value is above the permutation testing critical value. Thus, given redundant model as ground truth, permutation-testing would result in false positives.

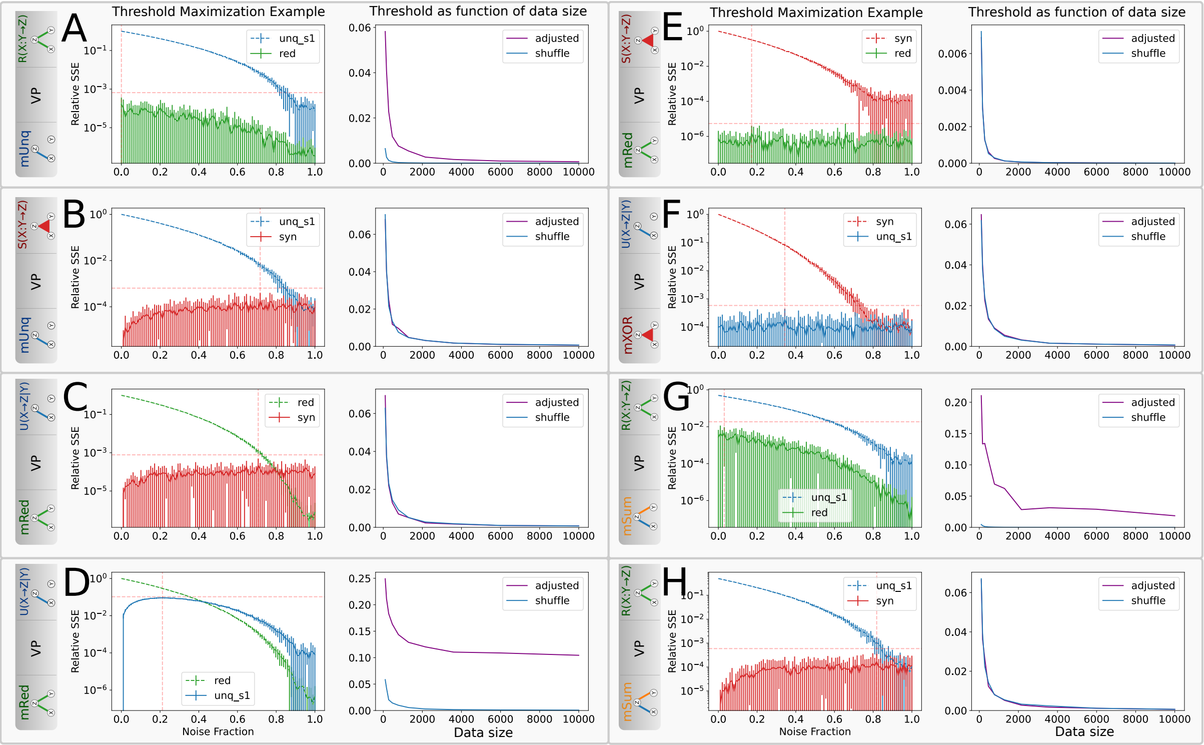

Figure 41: Adjusted maximization of critical value for **VP** for various **discrete** models and information atoms. Figure structure same as in fig. 40. As described in the main text, in many combinations of ground truth model, observable model and the information atom the adjusted critical value does not exceed permutation-testing, suggesting there are no false positives for those combinations. This is mostly true for synergistic neuronal model, but is not true for redundant and unique models.

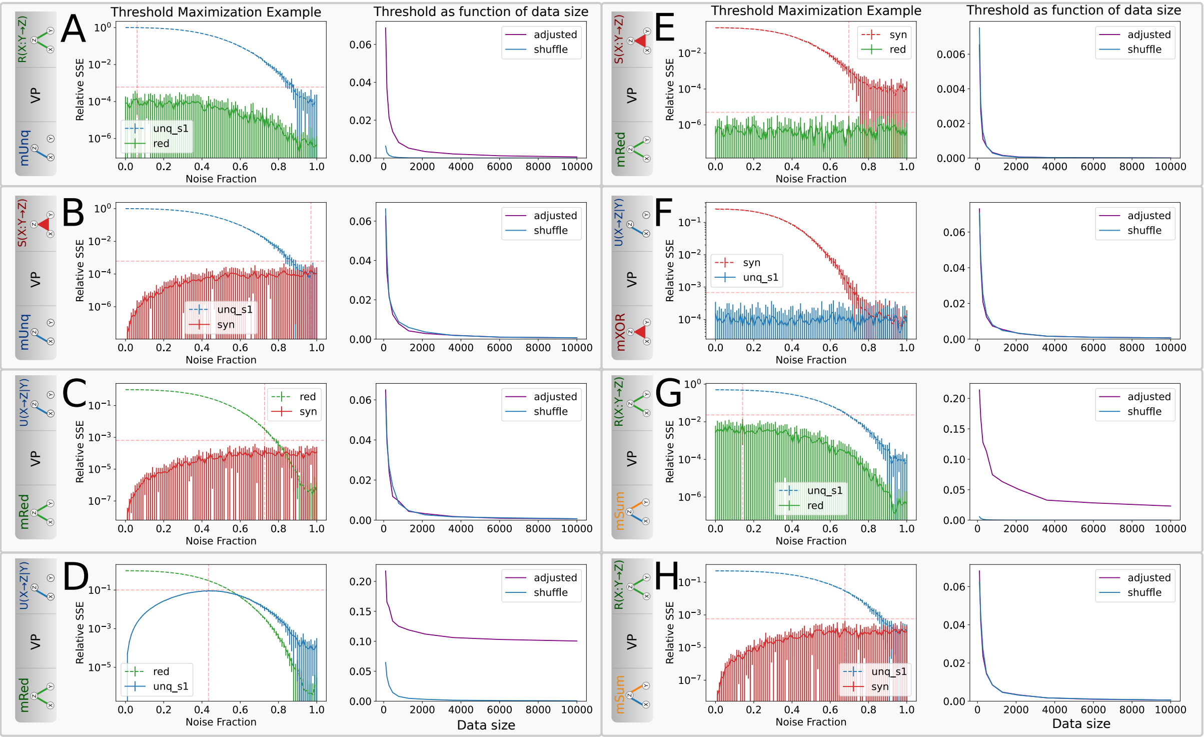

Figure 42: Adjusted maximization of critical value for **VP** for various **continuous** models and information atoms. Figure structure same as in fig. 40.

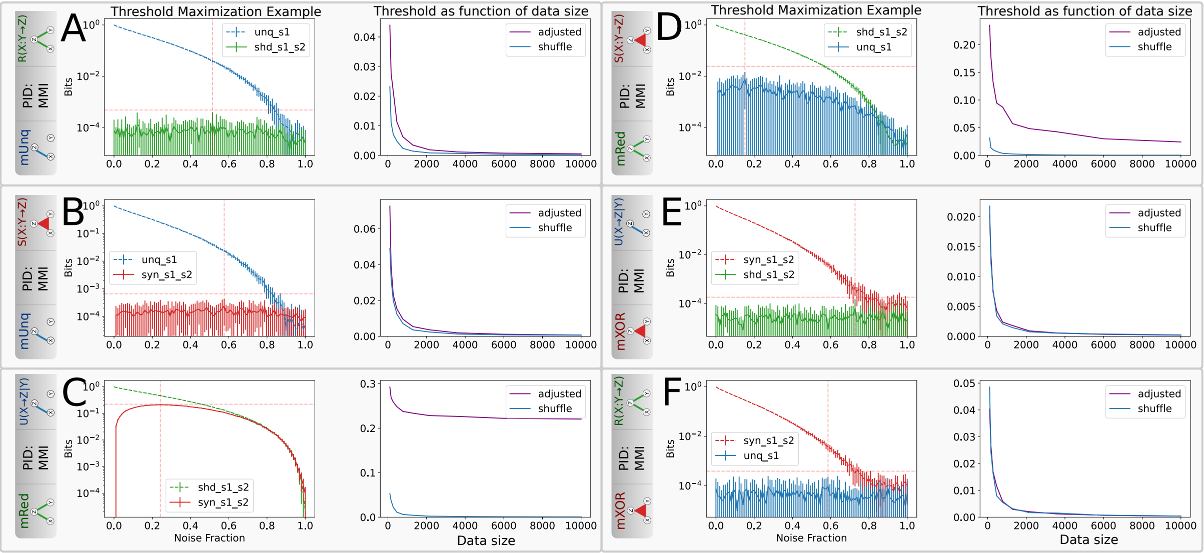

Figure 43: Adjusted maximization of critical value for **MMI PID** for various **discrete** models and information atoms. Figure structure same as in fig. 40.

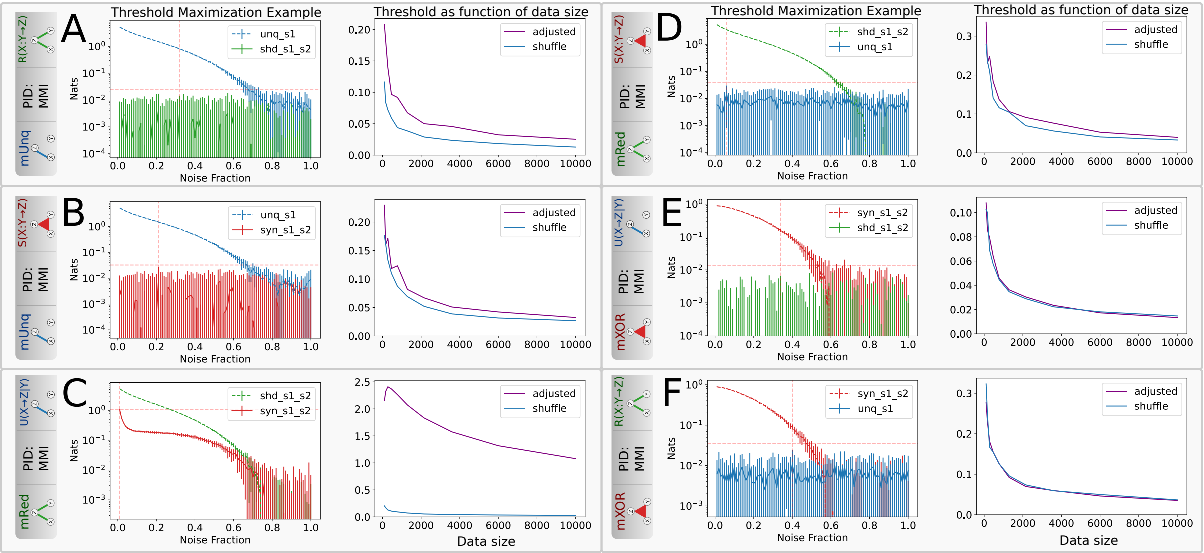

Figure 44: Adjusted maximization of critical value for **MMI PID** for various **continuous** models and information atoms. Figure structure same as in fig. 40.

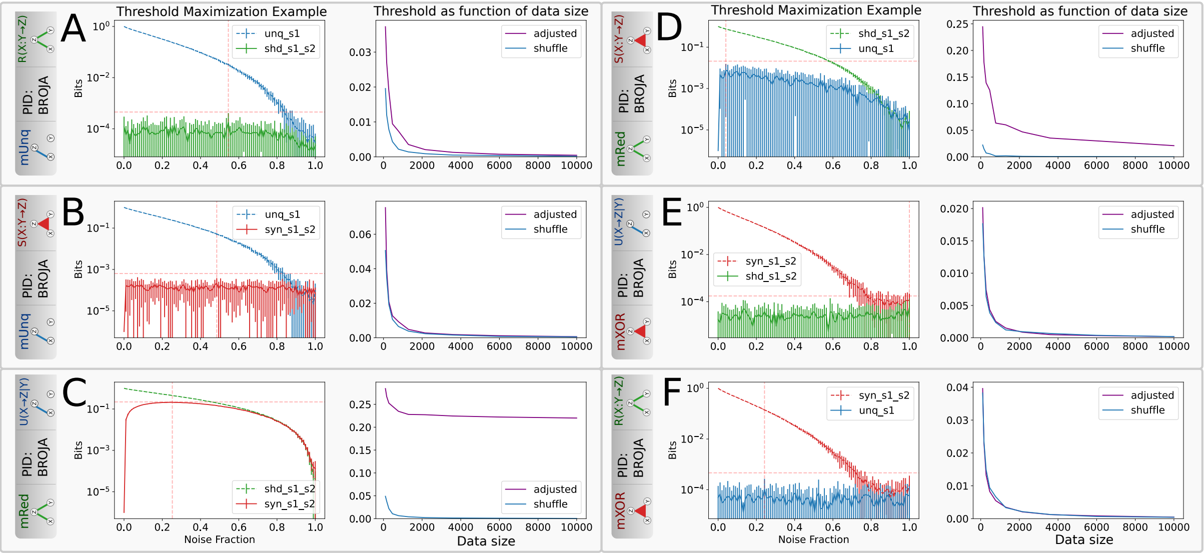

Figure 45: Adjusted maximization of critical value for **BROJA PID** for various **discrete** models and information atoms. Figure structure same as in fig. 40.
